# Multiorgan impairment in low-risk individuals with post-COVID-19 syndrome: a prospective, community-based study

**DOI:** 10.1136/bmjopen-2020-048391

**Published:** 2021-03-30

**Authors:** Andrea Dennis, Malgorzata Wamil, Johann Alberts, Jude Oben, Daniel J Cuthbertson, Dan Wootton, Michael Crooks, Mark Gabbay, Michael Brady, Lyth Hishmeh, Emily Attree, Melissa Heightman, Rajarshi Banerjee, Amitava Banerjee, COVERSCAN study investigators

**Affiliations:** 1 Perspectum, Oxford, UK; 2 Department of Cardiology, Great Western Hospital Foundation NHS Trust, Swindon, UK; 3 Department of Cardiology, Oxford University Hospitals NHS Foundation Trust, Oxford, UK; 4 Alliance Medical, Warwick, UK; 5 Department of Gastroenterology, Guy's and St Thomas' NHS Foundation Trust, London, UK; 6 Institute for Liver and Digestive Health, University College London, London, UK; 7 Institute of Cardiovascular and Metabolic Medicine, University of Liverpool, Liverpool, UK; 8 Institute of Infection and Global Health, University of Liverpool, Liverpool, UK; 9 Department of Respiratory Research, Liverpool University Hospitals NHS Foundation Trust, Liverpool, UK; 10 Department of Respiratory Medicine, Hull and East Yorkshire Hospitals NHS Trust, Hull, UK; 11 Institute of Clinical and Applied Health Research, University of Hull, Hull, UK; 12 Institute of Population Health Sciences, University of Liverpool, Liverpool, UK; 13 Department of Oncology, University of Oxford, Oxford, UK; 14 Long COVID SOS, Oxford, UK; 15 UKDoctors#Longcovid, London, UK; 16 Department of Medicine, University College London Hospitals NHS Foundation Trust, London, UK; 17 Institute of Health Informatics, University College London, London, UK; 18 Department of Cardiology, Barts Health NHS Trust, London, UK

**Keywords:** COVID-19, epidemiology, health policy, public health

## Abstract

**Objective:**

To assess medium-term organ impairment in symptomatic individuals following recovery from acute SARS-CoV-2 infection.

**Design:**

Baseline findings from a prospective, observational cohort study.

**Setting:**

Community-based individuals from two UK centres between 1 April and 14 September 2020.

**Participants:**

Individuals ≥18 years with persistent symptoms following recovery from acute SARS-CoV-2 infection and age-matched healthy controls.

**Intervention:**

Assessment of symptoms by standardised questionnaires (EQ-5D-5L, Dyspnoea-12) and organ-specific metrics by biochemical assessment and quantitative MRI.

**Main outcome measures:**

Severe post-COVID-19 syndrome defined as ongoing respiratory symptoms and/or moderate functional impairment in activities of daily living; single-organ and multiorgan impairment (heart, lungs, kidneys, liver, pancreas, spleen) by consensus definitions at baseline investigation.

**Results:**

201 individuals (mean age 45, range 21–71 years, 71% female, 88% white, 32% healthcare workers) completed the baseline assessment (median of 141 days following SARS-CoV-2 infection, IQR 110–162). The study population was at low risk of COVID-19 mortality (obesity 20%, hypertension 7%, type 2 diabetes 2%, heart disease 5%), with only 19% hospitalised with COVID-19. 42% of individuals had 10 or more symptoms and 60% had severe post-COVID-19 syndrome. Fatigue (98%), muscle aches (87%), breathlessness (88%) and headaches (83%) were most frequently reported. Mild organ impairment was present in the heart (26%), lungs (11%), kidneys (4%), liver (28%), pancreas (40%) and spleen (4%), with single-organ and multiorgan impairment in 70% and 29%, respectively. Hospitalisation was associated with older age (p=0.001), non-white ethnicity (p=0.016), increased liver volume (p<0.0001), pancreatic inflammation (p<0.01), and fat accumulation in the liver (p<0.05) and pancreas (p<0.01). Severe post-COVID-19 syndrome was associated with radiological evidence of cardiac damage (myocarditis) (p<0.05).

**Conclusions:**

In individuals at low risk of COVID-19 mortality with ongoing symptoms, 70% have impairment in one or more organs 4 months after initial COVID-19 symptoms, with implications for healthcare and public health, which have assumed low risk in young people with no comorbidities.

**Trial registration number:**

NCT04369807; Pre-results.

**Strengths and limitations of this study**

- This is an ongoing, prospective, longitudinal COVID-19 recovery study with biochemical and imaging characterisation of organ function, starting in April 2020 before recognition of ‘long-COVID’, proper testing availability and prospective COVID-19-related research.
- By recruiting ambulatory patients with broad inclusion criteria, we focused on a real-world population at lower risk of COVID-19 severity and mortality.
- Healthy controls were included for comparison, not individuals with postinfluenza symptoms, COVID-19 without symptoms or from general clinics, which further studies may explore.
- The study population was not ethnically diverse despite disproportionate COVID-19 impact in non-white individuals.
- To limit interaction and exposure between the trial team and the patients, pulse oximetry, spirometry, MRI assessment of the brain and muscle function were not included from the outset.

## Introduction

Early in the COVID-19 pandemic, research and clinical practice focused on pulmonary manifestations.^1^ There is increasing evidence for direct multiorgan effects,^2–7^ as well as indirect effects on other organ systems and disease processes, such as cardiovascular diseases and cancers, through changes in healthcare delivery and patient behaviours.^8–10^ The clear long-term impact on individuals and health systems underlines the urgent need for a whole body approach with assessment of all major organ systems following SARS-CoV-2 infection. Quantitative MRI has recently been used to show multiorgan impairment in individuals post-COVID-19 hospitalisation,^11^ but has not been used in non-hospitalised individuals.

COVID-19 is the convergence of an infectious disease, undertreated non-communicable diseases and social determinants of health, described as a ‘syndemic’.^12^ Pre-existing non-communicable diseases and risk factors predict poor COVID-19 outcomes, whether intensive care admission or mortality.^10^ Research has emphasised acute SARS-CoV-2 infection, hospitalised individuals and COVID-19 mortality,^13–15^ which is likely to underestimate the true burden of COVID-19-related disease. Among those surviving acute infection, 10% report persistent symptoms for 12 weeks or longer after initial infection (‘long-COVID’, or ‘post COVID-19 syndrome’, PCS).^16^ However, PCS is yet to be fully defined.^17–20^ Neither severity of symptoms, nor medium-term and long-term pathophysiology across organ systems, nor the appropriate control populations are understood.

UK government policies have emphasised excess mortality risk in moderate-risk and high-risk conditions, including ‘shielding’^10^ and commissioning of a risk calculator to identify those at highest risk of COVID-19 severity and mortality.^21^ These policies assume that younger individuals without apparent underlying conditions are at low risk. However, unlike symptoms following critical illness^22^ or acute phase of other coronavirus infections,^23^ symptoms in PCS are commonly reported in individuals with low COVID-19 mortality risk, for example, female, young and no chronic comorbidities.^14^ The potential scale of PCS in ‘lower-risk’ individuals, representing up to 80% of the population,^3^ necessitates urgent policies across countries to monitor,^24^ treat^19^ and pay^25^ for long-term implications of COVID-19 and to mitigate impact on healthcare utilisation and economies.

Therefore, in a pragmatic, prospective cohort study of individuals with persistent symptoms at least 4 weeks following recovery from acute SARS-CoV-2 infection and at low risk of COVID-19 mortality, we investigated (1) the prevalence of multiorgan impairment, compared with healthy, age-matched controls; (2) the associations between typical COVID-19 symptoms and multiorgan impairment; and (3) the associations between hospitalisation, severity of symptoms and multiorgan impairment.

## Methods

### Patient population and study design

**Figure 1:**
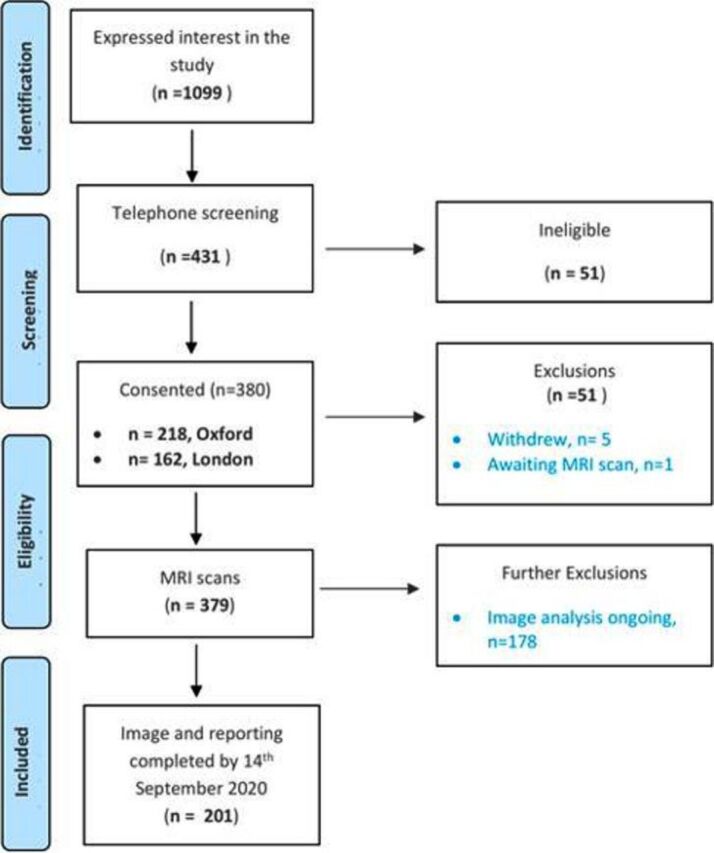
Flow from recruitment to enrolment of 201 patients with post-COVID-19 syndrome.

In an ongoing, prospective study, participants were recruited to the study following expression of interest on the study registration website. Participants learnt about the study through advertisement on social media or via recommendations from clinicians from four participant identification centres, the latter usually applied to patients who had been hospitalised. Assessment took place at two UK research imaging sites (Perspectum, Oxford; and Mayo Clinic Healthcare, London) between 1 April 2020 and 14 September 2020, completing baseline assessment by 14 September 2020 (figure 1). Participants with laboratory-confirmed SARS-CoV-2 infection (tested SARS-CoV-2-positive by oropharyngeal/nasopharyngeal swab by reverse-transcriptase PCR (n=62), a positive antibody test (n=63), or with strong clinical suspicion of infection with typical symptoms/signs and assessed as highly likely to have COVID-19 by two independent clinicians (n=73)) were eligible for enrolment. Exclusion criteria were symptoms of active respiratory viral infection (temperature >37.8°C or three or more episodes of coughing in 24 hours), hospital discharge in the last 7 days, and contraindications to MRI, including implanted pacemakers, defibrillators, other metallic implanted devices and claustrophobia. All participants gave written informed consent.

### Assessment of PCS

Assessment included patient-reported validated questionnaires (quality of life, EQ-5D-5L,^26^ and Dyspnoea-12^27^) and fasting biochemical investigations (listed in online supplemental methods). PCS was classified as ‘severe’ (defined as persistent breathlessness, score of ≥10 on Dyspnoea-12, or reported moderate or greater problems with usual activities on EQ-5D-5L) or ‘moderate’. These thresholds were selected as the Dyspnoea-12 has been correlated with the Medical Research Council (MRC) dyspnoea grade, where level 3 warrants referral to rehabilitation services,^27^ and with EQ-5D-5L, less than 8% of the general population report moderate or greater problems with usual activities.^28^

10.1136/bmjopen-2020-048391.supp3 Supplementary data

### Multiorgan impairment in PCS compared with healthy controls

We selected MRI as the imaging modality (as in UK Biobank) due to (1) safety (no radiation exposure, no need for intravenous contrast and minimal contact with the radiographer); (2) quantitative reproducibility (>95% acquisition and image processing success rate); (3) capacity for information sharing (digital data repository for independent analysis and research); and (4) rapid scalability (35 min scan to phenotype lung, heart, kidney, liver, pancreas and spleen). Multiorgan MRI data were collected at both study sites (Oxford: MAGNETOM Aera 1.5T; Mayo Healthcare London: MAGNETOM Vida 3T; both from Siemens Healthcare, Erlangen, Germany). The COVERSCAN multiparametric MRI assessment typically required 35 min per patient, including the lungs, heart, liver, pancreas, kidneys and spleen, by standardised methodology (online supplemental file 1). In brief, we assessed inflammation of the heart, kidneys, liver and pancreas with quantitative T1 relaxation mapping; lung function was characterised with a dynamic structural T2-weighted lung scan estimating lung capacity; ectopic fat accumulation in the liver and pancreas from proton density fat fraction; and volume of the liver and spleen measured from T1-weighted structural scan.

To determine impairment in each organ, we compared MRI-derived measurements from the heart, lungs, kidneys, liver, pancreas and spleen with reference ranges (online supplemental table 1), which were established as mean±2 SD from the healthy, age-matched control subjects (n=36) and validated by scoping literature review.^11^ We defined organ impairment if quantitative T1 mapping was outside the reference ranges for the heart, kidney, liver and pancreas, reduced estimated lung capacity from dynamic measurements in the lungs, or there was evidence of hepatomegaly, splenomegaly or ectopic fat accumulation.

10.1136/bmjopen-2020-048391.supp2 Supplementary data

### Symptoms and multiorgan impairment

Associations between organ impairment and symptoms were visually assessed using a heat map, dividing those with impairments to an organ into columns and colouring the rows by percentage of reported symptoms.

### Hospitalisation, severity and multiorgan impairment

We compared mean differences in quantitative organ metrics for hospitalised versus not hospitalised and moderate versus severe PCS using Kruskal-Wallis test (Fisher’s exact test for differences in binary outcomes). We defined multiorgan impairment as ≥2 organs with metrics outside the reference range. We investigated the associations between multiorgan impairment and (1) being hospitalised and (2) severe PCS with multivariate logistic regression models, adjusting for age, sex and body mass index (BMI).

### Patient and public involvement and engagement

Patients and the public have directly and indirectly informed our research, from design to dissemination, with regular updates and webinars, including question and answer sessions with patients. Several clinician coauthors were indirectly informed by their patients in the COVERSCAN study (RB, AB) or PCS clinics (DW, MH, MC), who are members of organisations such as Long Covid SOS (eg, LH) and UKDoctors#Longcovid (eg, EA). LH and EA have been involved in the research, interpretation of results, understanding implications of our results and providing critical feedback to the manuscript.

### Statistical analysis

We performed all analyses using R V.3.6.1, using descriptive statistics to summarise baseline characteristics and considering a p value less than 0.05 as statistically significant. Mean and SD were used for normally distributed continuous variables, median with IQR for non-normally distributed variables, and frequency and percentage for categorical variables. For group-wise comparison for absolute values between cases and healthy controls, we used Kruskal-Wallis test.

## Results

### Overall study population

#### Baseline characteristics

The study included 201 individuals (full details regarding hospitalisation: n=199; full questionnaire data to assign PCS severity: n=193). The mean age was 44.0 (range 21–71) years and the median BMI was 25.7 (IQR 23–28). Of the individuals, 71% were female, 88% were white, 32% were healthcare workers and 19% had been hospitalised with COVID-19. Assessments (symptoms, blood and MRI) had a median of 141 (IQR 110–162) days after initial symptoms. Medical history included smoking (3%), asthma (19%), obesity (20%), hypertension (7%), diabetes (2%) and prior heart disease (5%). The healthy control group had a mean age of 39 years (range 20–70), 40% were female, with a median BMI of 23 (IQR: 21–25) (table 1).

**Table 1:** Baseline demographics and symptoms of 201 low-risk individuals with post-COVID-19 syndrome

	All patients (N=201)	Healthy controls (n=36)	P value	Not hospitalised (n=163)	Hospitalised (n=37)	P value	Moderate PCS (n=77)	Severe PCS (n=116)	P value
Age (years), mean (SD)	44 (11.0)	39 (12.4)	0.013	43 (10.9)	50 (10.0)	0.001	45 (12.2)	44 (10.0)	0.419
Female, n (%)	142 (70.6)	14 (38.9)	0.032	118 (72.4)	23 (62.2)	0.302	51 (66.2)	85 (73.3)	0.374
BMI (kg/m^2^), median (IQR)	25.7 (22.7–28.1)	23.2 (21.4–23.1)	<0.001	25.3 (22.7–27.7)	27.2 (23.1–31.0)	0.063	25.8 (22.7–27.9)	25.4 (22.5–28.2)	0.639
Ethnicity									
White	176 (87.6)	33 (91.7)		148 (90.8)	28 (75.7)		67 (87.0)	106 (91.4)	0.178
Mixed	3 (1.5)	0 (0)	0.904	3 (1.8)	0 (0)	0.016	1 (1.3)	2 (1.7)	
South Asian	7 (3.5)	3 (8.3)		4 (2.5)	3 (8.1)		5 (6.5)	0 (0)	
Black	4 (2.0)	0 (0)		1 (0.6)	2 (5.4)		2 (2.6)	2 (1.7)	
Comorbidities and risks									
Smoking									0.244
Never	133 (66.2)	20 (60.6)		108 (66.3)	24 (64.9)		55 (71.4)	72 (61.7)	
Current	6 (3.0)	8 (24.2)	<0.001	6 (3.7)	0 (0)	0.641	3 (3.9)	3 (2.6)	
Ex-smoker	62 (30.8)	5 (15.2)		49 (30.1)	13 (35.1)		19 (24.7)	41 (35.3)	
Healthcare worker	64 (31.8)	4 (12.1)	0.009	50 (30.7)	13 (35.1)	0.695	33 (42.9)	28 (24.1)	0.007
Asthma	37 (18.4)	0 (0)	0.002	34 (20.9)	3 (8.1)	0.099	13 (16.9)	22 (19.0)	0.849
BMI									
≥25 kg/m^2^	113 (56.5)	7 (20)		87 (53.7)	25 (67.6)	0.144	46 (60.5)	62 (53.4)	0.374
≥30 kg/m^2^	40 (20.0)	0 (0)		28 (17.3)	12 (32.4)	0.066	16 (21.1)	24 (20.7)	1.000
Hypertension	13 (6.5)	0 (0)	0.001	11 (6.7)	2 (5.4)	1.000	6 (7.8)	7 (6.0)	0.771
Diabetes	4 (2.0)	0 (0)	0.104	4 (2.5)	0 (0.0)	1.000	4 (5.2)	0 (0.0)	0.024
Previous heart disease	9 (4.5)	0 (0)	0.001	8 (4.9)	1 (2.7)	1.000	3 (3.9)	5 (4.3)	1.000
Symptoms									
Fatigue	196 (98.0)			159 (97.5)	37 (100.0)	1.000	73 (96.1)	115 (99.1)	0.302
Shortness of breath	176 (88.0)			141 (86.5)	35 (94.6)	0.262	58 (76.3)	112 (96.6)	<0.0001
Muscle ache	173 (86.5)			142 (87.1)	31 (83.8)	0.597	66 (86.8)	101 (87.1)	1.000
Headache	165 (82.5)			138 (84.7)	27 (73.0)	0.098	56 (73.7)	102 (87.9)	0.019
Joint pain	156 (78.0)			127 (77.9)	29 (78.4)	1.000	56 (73.7)	94 (81.0)	0.284
Chest pain	152 (76.0)			128 (78.5)	24 (64.9)	0.090	47 (61.8)	98 (84.5)	0.001
Cough	146 (73.0)			117 (71.8)	29 (78.4)	0.539	55 (72.4)	84 (72.4)	1.000
Fever	144 (72.0)			113 (69.3)	31 (83.8)	0.104	51 (67.1)	86 (74.1)	0.329
Sore throat	143 (71.5)			120 (73.6)	23 (62.2)	0.165	50 (65.8)	86 (74.1)	0.256
Diarrhoea	118 (59.0)			91 (55.8)	27 (73.0)	0.065	40 (52.6)	76 (65.5)	0.097
Abnormal pain	108 (54.0)			91 (55.8)	17 (45.9)	0.361	30 (39.5)	75 (64.7)	0.001
Wheezing	98 (49.0)			75 (46.0)	23 (62.2)	0.101	30 (39.5)	64 (55.2)	0.039
Inability to walk	80 (40.0)			58 (35.6)	22 (59.5)	0.009	24 (31.6)	50 (43.1)	0.130
Runny nose	68 (34.0)			55 (33.7)	13 (35.1)	0.85	24 (31.6)	41 (35.3)	0.642
Time interval									
Initial symptoms to assessment (days), median (IQR)	141 (110–162)			141 (112–163)	138 (97–150)	0.106	121 (89–158)	145 (121–163)	0.001
COVID-19-positive to assessment (days), median (IQR)	71 (41–114)			68 (35–112)	105 (59–126)	0.012	60 (43–98)	78 (34–119)	0.305

Data are presented as count (%).

Comparisons between managed at home versus hospitalised and between moderate versus PCS were conducted using Fisher’s exact test

BMI, body mass index; PCS, post-COVID-19 syndrome.

Regardless of hospitalisation, the most frequently reported symptoms were fatigue (98%), shortness of breath (88%), muscle ache (87%) and headache (83%) (table 1). Of the individuals, 99% had four or more and 42% had ten or more symptoms. Of individuals 70% reported ≥13 weeks off paid employment. Of the incidental structural findings observed on MRI (n=56), three were cardiac (atrial septal defect, bicuspid aortic valve and right atrial mass), one renal (hydronephrosis) and the rest were benign cysts.

Haematological investigations, including mean corpuscular haemoglobin concentration (24%), and renal, liver and lipid biochemistry, including potassium (38%), alanine transferase (14%), lactate dehydrogenase (17%), triglycerides (11%) and cholesterol (42%), were abnormally high in ≥10% of individuals. Bicarbonate (10%), phosphate (11%), uric acid (11%) and transferrin saturation (19%) were abnormally low in ≥10% of individuals (online supplemental table 1).

### Single-organ and multiorgan impairment in PCS compared with healthy controls

**Figure 2:**
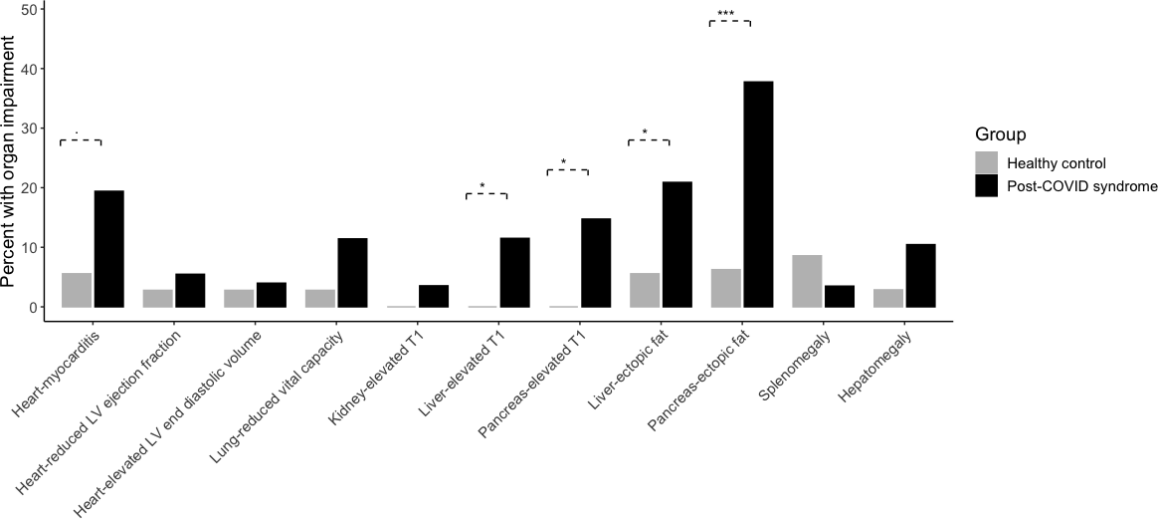
Percentage of patients (black) and controls (grey) with individual organ measures outside of the predefined normal range. Lines represent significant difference in the proportions between the two groups, with *p<0.05, **p<0.01, ***p<0.001. LV, left ventricular.

**Figure 3:**
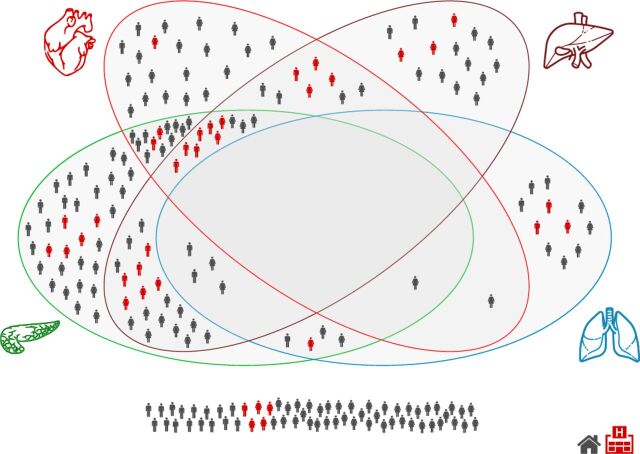
Multiorgan impairment in low-risk individuals with post-COVID-19 syndrome by gender and hospitalisation.

Organ impairment was more common in PCS than healthy controls (figure 2 and online supplemental figure 1). Impairment was present in the heart in 26% (myocarditis 19%, systolic dysfunction 9%), lung in 11% (reduced vital capacity), kidney in 4% (inflammation), liver in 28% (12% inflammation, 21% ectopic fat, 10% hepatomegaly), pancreas in 40% (15% inflammation, 38% ectopic fat) and spleen in 4% (splenomegaly) (figure 2 and table 2). Of the individuals, 70% had impairment in at least one organ and 29% had multiorgan impairment, with overlap across multiple organs (figure 3). Impairment in the liver, heart or lungs was associated with further organ impairment in 63%, 62% and 48% of individuals, respectively (figure 3).

10.1136/bmjopen-2020-048391.supp1 Supplementary data

**Table 2:** Evidence of organ impairment in 201 low-risk individuals with post-COVID-19 syndrome

Measurement	All patients (N=201)	Healthy controls (n=36)	P value	Not hospitalised (n=163)	Hospitalised (n=37)	P value	Moderate PCS (n=77)	Severe PCS (n=116)	P value
Heart									
Left ventricular ejection fraction (%)									
Normal (>51%)	190 (95.0)	35 (97.2)	0.699	155 (95.7)	33 (89.1)	0.124	72 (93.5)	111 (95.7)	0.353
Impaired (≤51%)	11 (5.0)	1 (2.8)		7 (4.3)	4 (10.1)		5 (6.4)	5 (4.3)	
Left ventricular end diastolic volume (mL)									
>264 mL in Men; >206 mL in Women	8 (4.0)	1 (2.8)	1.00	4 (2.5)	4 (10.8)	0.040	4 (5.2)	4 (3.4)	0.715
Evidence of myocarditis									
≥3 segments with high T1 (≥1229 ms at 3T; ≥1015 ms at 1.5T)	39 (19.4)	2 (5.6)	0.053	30 (18.4)	8 (21.6)	0.647	9 (11.7)	29 (25.0)	0.027
Lungs									
Deep breathing fractional area change	(n=17 missing)			(n=13 missing)	(n=3 missing)		(n=8 missing)	(n=7 missing)	
<31%	21 (11.4)	1 (2.8)	0.138	17 (11.3)	4 (11.8)	1	7 (10.1)	13 (11.9)	0.811
Kidneys									
Kidney cortex T1	(n=3 missing)			(n=3 missing)			(n=2 missing)		
Normal (<1652 ms at 3T; <1227 ms at 1.5T)	191 (96.5)	36 (100.0)	0.599	155 (96.9)	35 (94.6)	0.618	74 (98.7)	112 (96.6)	0.65
Impaired (≥1652 ms at 3T; ≥1227 ms at 1.5T)	7 (3.5)	0 (0.0)		5 (3.1)	2 (5.4)		1 (1.3)	4 (3.4)	
Pancreas									
Pancreatic inflammation (T1 in ms)	(n=11 missing)	(n=13 missing)		(n=7 missing)	(n=4 missing)		(n=4 missing)	(n=6 missing)	
Normal <803 ms	162 (85.3)	23 (100.0)	0.049	139 (89.1)	22 (66.7)	0.002	60 (82.2)	95 (86.4)	0.530
Impaired ≥803 ms	28 (14.7)	0 (0)		17 (10.9)	11 (33.3)		13 (17.8)	15 (13.6)	
Pancreatic fat		(n=4 missing)							
Normal <4.6%	122 (62.2)	30 (93.8)	<0.001	107 (66.9)	14 (40.0)	0.004	44 (57.9)	72 (63.7)	0.449
Impaired ≥4.6%	74 (37.8)	2 (6.2)		53 (33.1)	21 (60.0)		32 (42.1)	41 (36.3)	
Liver									
Liver inflammation (cT1 in ms)	(n=1 missing)			(n=1 missing)			(n=1 missing)		
Normal <784 ms	177 (88.5)	36 (100)	0.030	148 (91.4)	28 (75.7)	0.018	69 (90.8)	101 (87.1)	0.494
Impaired ≥784 ms	23 (11.5)	0 (0)		14 (8.6)	9 (24.3)		7 (9.2)	15 (12.9)	
Liver fat									
Normal <4.8%	159 (79.1)	34 (94.4)	0.034	134 (82.2)	24 (64.9)	0.026	61 (79.2)	91 (78.4)	1
Impaired ≥4.8%	42 (20.9)	2 (5.4)		29 (17.8)	13 (35.1)		16 (20.8)	25 (21.6)	
Liver volume		(n=1 missing)							
Normal <1935 mL	180 (89.6)	34 (97.1)	0.214	154 (94.5)	25 (67.6)	<0.0001	68 (88.3)	104 (89.7)	0.816
Impaired ≥1935 mL	21 (10.4)	1 (2.9)		9 (5.5)	12 (32.4)		9 (11.7)	12 (10.3)	
Spleen									
Splenic volume (mL)		(n=1 missing)							
Normal <350 mL	194 (96.5)	32 (91.4)	0.172	160 (98.2)	33 (89.2)	0.023	74 (96.1)	112 (96.6)	1
Impaired ≥350 mL	7 (3.5)	3 (8.6)		3 (1.8)	4 (10.8)		3 (3.9)	4 (3.4)	

Data are presented as count (%).

Comparisons between managed at home versus hospitalised and between moderate versus PCS were conducted using Fisher’s exact test.

PCS, post-COVID-19 syndrome.

### Symptoms and multiorgan impairment

**Figure 4:**
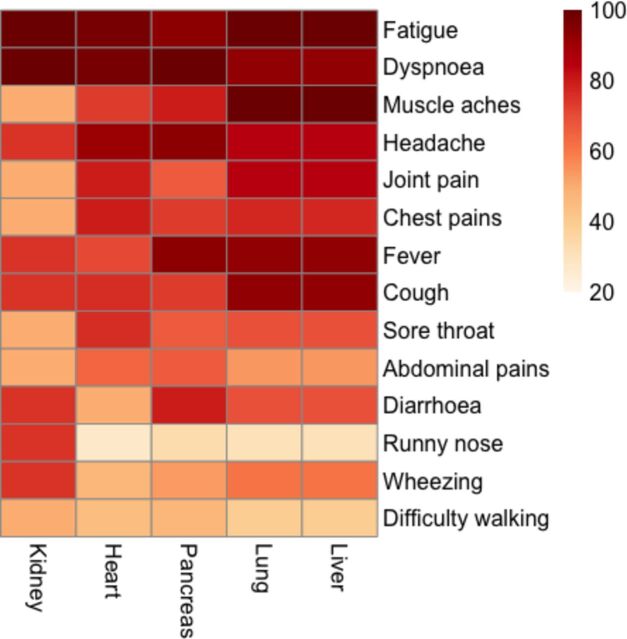
Percentage of reported symptoms during the acute phases of the illness within those with evidence of organ impairment for each organ separately. Darker red indicates higher percentage of reported symptoms per impaired organ. There are no distinct patterns of symptoms relating to each impaired organ, but a high burden of symptoms in individuals is highlighted.

Hepatic and pulmonary impairment frequently clustered together, with fatigue, muscle aches, fever and cough commonly reported. Impairment in particular organs was associated with particular symptoms—pancreas: diarrhoea, fever, headache and dyspnoea; heart: headache, dyspnoea and fatigue; and kidney: wheezing, runny nose, diarrhoea, cough, fever, headache, dyspnoea and fatigue (figure 4).

### Hospitalisation, severity and multiorgan impairment

The hospitalised group were older (p=0.001), had higher BMI (p=0.063), and were more likely to be non-white (p=0.016) and to report ‘inability to walk’ (p=0.009) than non-hospitalised individuals. There were no other statistically significant differences between risk factors or symptoms between the groups. Impairment of the liver, pancreas (eg, ectopic fat in the pancreas and liver, hepatomegaly) and ≥2 organs was higher in hospitalised individuals (all p<0.05) (figure 3 and table 2). In multivariate analyses, adjusting for age, sex and BMI, liver volume remained significantly associated with hospitalisation (p=0.001). Hospitalised individuals had high triglycerides (30% vs 7.2%, p=0.002), cholesterol (60% vs 38%, p=0.04) and low-density lipoprotein-cholesterol (57% vs 31%, p=0.01), and low transferrin saturation (38% vs 15%, p=0.01), compared with non-hospitalised individuals. erythrocyte sedimentation rate (ESR) (13%), bicarbonate (12%), uric acid (16%), platelet count (13%) and high-sensitivity C-reactive protein (CRP) (15%) were high in ≥10% of hospitalised individuals.

Of the individuals, 60% (n=120) had severe PCS, with 52% reporting persistent, moderate problems undertaking usual activities (level 3 or greater in the relevant EQ-5D-5L question; 34% reported Dyspnoea-12 score ≥10). Of those with severe PCS, 84% were not hospitalised and 73% were female. There were no differences in age, BMI or ethnicity between the groups. Individuals with severe PCS were more likely to report shortness of breath (p<0.001), headache (p=0.019), chest pain (p=0.001), abdominal pain (p=0.001) and wheezing (p=0.039). Of those with ‘severe’ PCS, 25% had myocarditis compared with 12% with moderate PCS (unadjusted: 0.023; adjustment for age, sex and BMI: p=0.04; online supplemental figure 2). Severe PCS was associated with higher mean cell haemoglobin concentration (28% vs 17%), cholesterol (46.2% vs 32.8%), CRP (10% vs 3.8%) and ESR (10% vs 6%) than moderate PCS, but these differences were not statistically significant (online supplemental table 3). Muscle aches, fever and coughing were common in severe PCS, and headache was common in individuals with inflammation of the pancreas (figure 4).

## Discussion

We report three findings in the first COVID-19 recovery study to evaluate medium-term, multiorgan impairment. First, in low-risk individuals, there were chronic symptoms and mild impairment in the heart, lung, liver, kidney and pancreas 4 months post-COVID-19, compared with healthy controls. Second, cardiac impairment was more common in severe PCS. Third, we demonstrate feasibility and potential utility of community-based multiorgan assessment for PCS.

### Comparison with other studies

Common symptoms were fatigue, dyspnoea, myalgia, headache and arthralgia, despite low risk of COVID-19 mortality or hospitalisation. COVID-19 impact models have included age, underlying conditions and mortality, but not morbidity, multiorgan impairment and chronic diseases.^29 30^ Even in non-hospitalised individuals, up to 10% of those infected have PCS,^15 31^ but studies of extrapulmonary manifestations emphasise acute illness.^32^ We describe mild rather than severe organ impairment, but the pandemic’s scale and high infection rates in lower risk individuals signal medium-term and longer-term COVID-19 impact, which cannot be ignored in healthcare or policy spheres.

Acute myocarditis and cardiogenic shock^33^ are documented in hospitalised patients with COVID-19.^6^ In American athletes, recent COVID-19 was associated with myocarditis.^34^ Although causality cannot be attributed and postviral syndromes have included similar findings,^21^ we show that a quarter of low-risk individuals with PCS have mild systolic dysfunction or myocarditis. The significance of these findings and the associations with contemporaneous abnormal echocardiography findings and long-term myocardial fibrosis and impairment are unknown. Cardiac impairment, a risk factor for severe COVID-19, may have a role in PCS. Two further findings that deserve investigation are pancreatic abnormalities, given the excess diabetes risk reported in PCS,^15^ and the preponderance of healthcare workers at increased PCS risk (as observed for COVID-19 mortality), possibly due to higher viral burden.

PCS is likely to be a syndrome rather than a single condition. Despite an immunological basis for individual variations in COVID-19 progression and severity,^35^ prediction models have high rates of bias, perform poorly,^36^ and focus on respiratory dysfunction and decisions for ventilation in acutely unwell patients, rather than multiorgan function. Ongoing long-term studies^37^ exclude non-hospitalised, low-risk individuals. During a pandemic, we studied subclinical organ impairment in PCS, showing low rates of incidental findings. As specialist PCS services are rolled out,^38 39^ multiorgan assessment, monitoring and community pathways have potential roles during and beyond COVID-19, but need to be evaluated.

### Implications for research, clinical practice and public health

**Figure 5:**
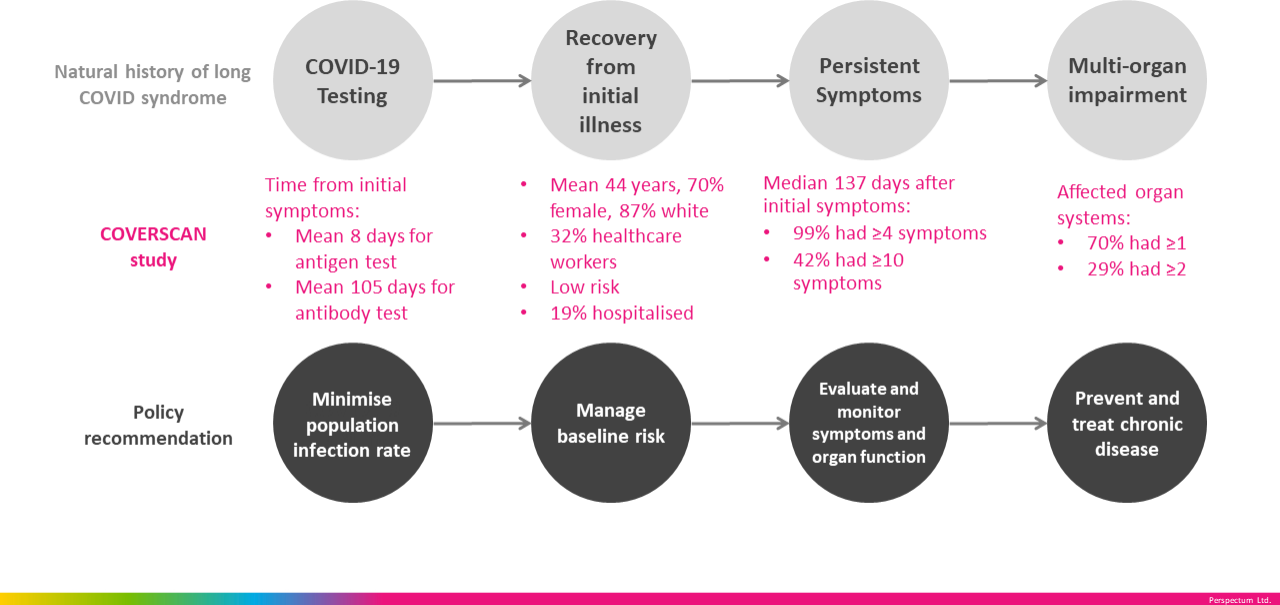
Natural history of post-COVID-19 syndrome, the COVERSCAN study in low-risk individuals (N=201) and policy recommendations.

Our findings have three research implications. First, as countries face second waves, COVID-19 impact models should include PCS, whether quality of life, healthcare utilisation or economic effects. Second, there is urgent need for multiorgan assessment, including blood and imaging, as well as primary and secondary care data linkage, to define PCS. Third, longitudinal studies of clustering of symptoms and organ impairment will inform health services research to plan multidisciplinary care pathways. There are three management implications. First, we signal the need for multiorgan monitoring in at least the medium term, especially extrapulmonary sequelae. Care pathways involving MRI (with limited access in many clinical settings) need evaluation versus other modalities to detect organ impairment (eg, spirometry, N-terminal pro B-type natriuretic peptide (NT-pro-BNP), ECG, echocardiography, ultrasound and blood investigations). Second, until effective vaccines and treatments are widely available, ‘infection suppression’ (eg, social distancing, masks, physical isolation) is the prevention strategy. Third, whether understanding baseline risk or multiorgan complications, PCS requires management across specialties (eg, cardiology, gastroenterology) and disciplines (eg, epidemiology, diagnostics, laboratory science) (figure 5).

### Limitations

There are some limitations. First, our cardiac MRI protocol excluded gadolinium contrast due to concerns regarding COVID-19-related renal complications, relying on native T1 mapping to characterise myocardial inflammation non-invasively (previously validated for acute myocarditis).^40^ Second, for organ impairment, we show association, not causation, and incidental findings are possible in asymptomatic individuals^41^; however, our findings are strengthened by comparison with healthy, age-matched controls, although not matched for sex or baseline comorbidities. Third, for pragmatic reasons, our controls were scanned using 1.5T, but we used 3T ranges as described in an analogous study with similar acquisition protocols. Therefore, we may be under-representing the true proportion of impairment in those individuals with PCS scanned at 3T. Fourth, further studies may explore different controls, for example, individuals with postinfluenza symptoms, COVID-19 without symptoms or from general clinics. We will investigate duration, trajectory, complications and recovery for specific symptoms and organ impairment in the follow-up phase. Fifth, our study population was not ethnically diverse, despite disproportionate COVID-19 impact in non-white individuals. Sixth, to limit interaction and exposure between the trial team and the patients, pulse oximetry, spirometry, MRI assessment of the brain and muscle function were not included from the outset.

## Conclusions

Our study suggests PCS has a physiological basis, with measurable patient-reported outcomes and organ impairment. Future research should address longer-term follow-up of organ function beyond symptoms and blood investigations, even in lower risk individuals; prioritisation for imaging, investigation and referral; and optimal care pathways. Health system responses should emphasise infection suppression and management of pre-COVID-19 and post-COVID-19 risk factors and chronic diseases.

## Supplementary Material

Author's manuscript

## Data Availability

Data are available upon reasonable request from the corresponding author.
