# Supplementary material for: Multiorgan impairment in low-risk individuals with post-COVID-19 syndrome: a prospective, community-based study
Source: BMJ Open. 2021 Mar 30;11(3):e048391. doi: 10.1136/bmjopen-2020-048391 (PMC8727683; doi:10.1136/bmjopen-2020-048391)
Supplement: Supplementary data [file bmjopen-2020-048391supp002.pdf]

Web Supplementary Materials

Supplementary methods 2

Supplementary references 4

Supplementary results 6

Figure S1: Comparison of patients to control quantitative image derived measures in a subset of those scanned at 1.5T 6

Figure S2: Organ impairment in severe versus moderate post COVID syndrome (n=201) 7

Table S1: Reference ranges to define organ impairment 7

Table S2: Blood investigations in 201 low-risk individuals with post-COVID syndrome, sub-divided by hospitalisation or managed at home 8

Table S3: Blood investigations in 201 low-risk individuals, sub-divided by those with severe of moderate post-COVID syndrome 13

Supplementary methods

Blood investigations

Blood investigations included: full blood count, serum biochemistry (sodium, chloride, bicarbonate, urea, creatinine, bilirubin, alkaline phosphatase, aspartate transferase, alanine transferase, lactate dehydrogenase, creatinine kinase, gamma-glutamyl transpeptidase, total protein, albumin, globulin, calcium, magnesium, phosphate, uric acid, fasting triglycerides, cholesterol (total, HDL, LDL), iron, iron-binding capacity (unsaturated and total) and inflammatory markers (erythrocyte sedimentation rate, ESR; high sensitivity-C-Reactive Protein, CRP) (TDL laboratories, London).

Imaging

All the imaging methods can be deployed on standard clinical MRI scanners and are generally expedited approaches of methods previously demonstrated in the scientific literature that unless stated each utilise a short (<14seconds) breath-hold.

Cardiac imaging involved complete coverage of the heart with a short-axis stack (to the valve plane) of cine images acquired using cardiac gating, this acquisition mirrors that in UK Biobank and is a standardized approach(S1). Three short-axis cardiac T1 maps are acquired using the MOLLI-T1 approach at the basal, mid and apical levels of the left ventricle.

Liver and pancreas imaging used the LiverMultiScan acquisition protocol (Perspectum, Oxford, UK), which involves 3 single 2D axial slice breath-held acquisitions that separately are sensitive to the fat content (proton density fat fraction, or PDFF), to T2* (which is representative of liver iron content) and a MOLLI-T1 measurement (providing a measurement of tissue water), additionally a volumetric scan was used that covers the entire liver(S2).

Two dynamic cine MR acquisitions of the lung were acquired in the coronal plane with a 306.91 ms temporal resolution: one 40 s acquisition with the patient instructed to breathe normally and a second 30 s acquisition with the patient instructed to breathe deeply.

Kidney imaging used a single coronal view that was able to image both kidneys, imaging contrasts were MOLLI-T1, T2* (for blood oxygen level assessment), and diffusion imaging that was acquired during free-breathing in 2minutes.

Image Analysis

Cardiac MRI Analysis: Experienced cardiac MRI analysts used CVI42 (Cardiovascular Imaging Inc, Canada) to manually trace the end-diastolic and end-systolic phases in each of the short-axis views, following the standard UK BioBank evaluation approach as previously described(S3). This analysis yielded: For both the left and the right ventricle; End diastolic volume, End systolic volume, Stroke volume and Ejection Fraction. Additionally left ventricular muscle mass and wall thickness are determined from the function data. Cardiac T1 was determined for each of the 16 cardiac segments (of the AHA 17 segment model)(S4).

Liver Images were analysed by data analysts experienced at using the LiverMultiScan (Perspectum, Oxford, UK) software. This yielded global metrics in each liver of PDFF (proton density fat fraction), T2*, and cT1 (cT1 is a measurement of T1 that has been corrected for the confounding effects of iron and standardised to 3 Tesla; it is elevated with disease).

Pancreas images were analysed in a similar manner to the above except the software used was not FDA-cleared and iron correction was not performed. The output T1 was standardized to 3 Tesla.

Lung cine imaging allowed the measurement of the area of the left and right lungs through the breathing cycle in the coronal plane, which used automated methods that were reviewed by image analysts. The periodicity of the area fluctuations was used to determine the respiratory rate. All analysis was performed in-house using MATLAB based tools. The method was validated by measuring the correlation between the change in area and the forced vital capacity, the latter being measured using spirometry.

Patient respiration was assessed by imaging a single 2D coronal slice of the lungs over 30 seconds using a dynamic cine MRI acquisition, during which the patient instructed to breathe deeply.

Kidney images were assessed using in-house tools to fit the parametric maps and allow trained analysts to make measurements. The T2* maps were analysed by the Twelve Layer Concentric Object (TLCO) approach that generates a gradient of relaxation values, in the other evaluations the cortex and medulla were manually segmented using the MOLLI-T1 map or the b=0 (in the case of diffusion) to guide the boundary.

In all cases the volumetric assessments utilised an initial in-house developed machine-learning driven segmentation, and then a manual step that may be used to fine tune boundaries. This approach was also used in the body composition analysis, which for reasons of speed was performed only in a single slice (an axial view that passes through L3 of the spine) in this work.

Dennis A, *et al. BMJ Open* 2021; 11:e048391. doi: 10.1136/bmjopen-2020-048391

Supplementary references

S1. Petersen SE, Matthews PM, Francis JM, Robson MD, Zemrak F, Boubertakh R, Young AA, Hudson S, Weale P, Garratt S, Collins R, Piechnik S, Neubauer S. UK Biobank's cardiovascular magnetic resonance protocol. J Cardiovasc Magn Reson. 2016 Feb 1;18:8.

S2. Banerjee R, Pavlides M, Tunnicliffe EM, Piechnik SK, Sarania N, Philips R, Collier JD, Booth JC, Schneider JE, Wang LM, Delaney DW, Fleming KA, Robson MD, Barnes E, Neubauer S. Multiparametric magnetic resonance for the non-invasive diagnosis of liver disease. J Hepatol. 2014 Jan;60(1):69-77. doi: 10.1016/j.jhep.2013.09.002.

S3. Petersen SE, Aung N, Sanghvi MM, Zemrak F, Fung K, Paiva JM, Francis JM, Khanji MY, Lukaschuk E, Lee AM, Carapella V, Kim YJ, Leeson P, Piechnik SK, Neubauer S. Reference ranges for cardiac structure and function using cardiovascular magnetic resonance (CMR) in Caucasians from the UK Biobank population cohort. J Cardiovasc Magn Reson. 2017 Feb 3;19(1):18.

S4. Cerqueira MD, Weissman NJ, Dilsizian V, Jacobs AK, Kaul S, Laskey WK, Pennell DJ, Rumberger JA, Ryan T, Verani MS; American Heart Association Writing Group on Myocardial Segmentation and Registration for Cardiac Imaging. Standardized myocardial segmentation and nomenclature for tomographic imaging of the heart. A statement for healthcare professionals from the Cardiac Imaging Committee of the Council on Clinical Cardiology of the American Heart Association. Circulation. 2002 Jan 29;105(4):539-42.

S5. Kawel-Boehm N, Maceira A, Valsangiacomo-Buechel ER, Vogel-Claussen J, Turkbey EB, Williams R, Plein S, Tee M, Eng J, Bluemke DA. Normal values for cardiovascular magnetic resonance in adults and children. J Cardiovasc Magn Reson. 2015 Apr 18;17(1):29.).

S6. Ponikowski P, Voors AA, Anker SD, Bueno H, Cleland JGF, Coats AJS, Falk V, González-Juanatey JR, Harjola VP, Jankowska EA, Jessup M, Linde C, Nihoyannopoulos P, Parissis JT, Pieske B, Riley JP, Rosano GMC, Ruilope LM, Ruschitzka F, Rutten FH, van der Meer P; ESC Scientific Document Group. 2016 ESC Guidelines for the diagnosis and treatment of acute and chronic heart failure: The Task Force for the diagnosis and treatment of acute and chronic heart failure of the European Society of Cardiology (ESC)Developed with the special contribution of the Heart Failure Association (HFA) of the ESC. Eur Heart J. 2016 Jul 14;37(27):2129-2200.

S7. Tsao CW, Lyass A, Larson MG, Cheng S, Lam CS, Aragam JR, Benjamin EJ, Vasan RS. Prognosis of adults with borderline left ventricular ejection fraction. JACC Heart Fail. 2016 Jun;4(6):502-10.

S8. Chalasani, Naga, et al. The diagnosis and management of nonalcoholic fatty liver disease: practice guidance from the American Association for the Study of Liver Diseases. Hepatology 67.1 (2018): 328-357

S9. Mojtahed A, Kelly C, Herlihy A, et al. Reference range of liver corrected T1 values in a population at low risk for fatty liver disease-a UK Biobank sub-study, with an appendix of interesting cases. Abdomimal Radiol 2019; 44: 72–84.

S10. Jayaswal AN, Levick C, Selvaraj EA, et al. Prognostic value of multiparametric MRI, transient elastography and blood-based fibrosis markers in patients with chronic liver disease. Liver Int 2020; in press. DOI:doi:10.1111/liv.14625.

S11. Jayaswal ANA, Levick C, Selvaraj EA, Dennis A, Booth JC, Collier J, Cobbold J, Tunnicliffe EM, Kelly M, Barnes E, Neubauer S, Banerjee R, Pavlides M. Prognostic value of multiparametric magnetic

resonance imaging, transient elastography and blood-based fibrosis markers in patients with chronic liver disease. Liver Int. 2020 Jul 30. doi: 10.1111/liv.14625

S12. Chouhan MD, Firmin L, Read S, Amin Z, Taylor SA. Quantitative pancreatic MRI: a pathology-based review. Br J Radiol. 2019 Jul;92(1099):20180941.

S13. Harrington KA, Shukla-Dave A, Paudyal R, Do RKG. MRI of the Pancreas. J Magn Reson Imaging. 2020 Apr 17. doi: 10.1002/jmri.27148.

S14. Gillis KA, McComb C, Patel RK, et al. Non-contrast renal magnetic resonance imaging to assess perfusion and corticomedullary differentiation in health and chronic kidney disease. Nephron 2016; 133: 183–92.

S15. Peperhove M, Vo Chieu VD, Jang M-S, et al. Assessment of acute kidney injury with T1 mapping MRI following solid organ transplantation. Eur Radiol 2018; 28: 44–50.

S16. Chow KU, Luxembourg B, Seifried E, Bonig H. Spleen size is significantly influenced by body height and sex: establishment of normal values for spleen size at us with a cohort of 1200 healthy individuals. Radiology 2015; 279: 306–13.

Supplementary results

Sub-group analysis

Data from healthy participants (n=36) scanned on the 1.5T Siemens MRI scanner were compared to the sub-group of patients (N=121) scanned on the same MRI machine. Median global cardiac T1 was elevated in the patient group (979 ms versus 962ms, P=0.001). Lung fractional area difference, a measure of relaxed vital capacity, was significantly lower in the patient group (41% versus 48%, P<.001). Kidney inflammation (1148 vs 1084 ms, p <0.001) was significantly elevated in the patients as were markers of organ fat (liver 2.6% versus 2.1%, p=0.008; pancreas: 4.3% versus 2.5%, p<0.001) (**Figure S1**).

Figure S1: Box plots showing median and interquartile ranges for the healthy control group and the patient group for those scanned at 1.5T. Comparisons between groups were performed using two-sided Kolmogorov-Smirnov (KS) tests. Significance stars are * P<.05; ** P<.01, ***P<.001.

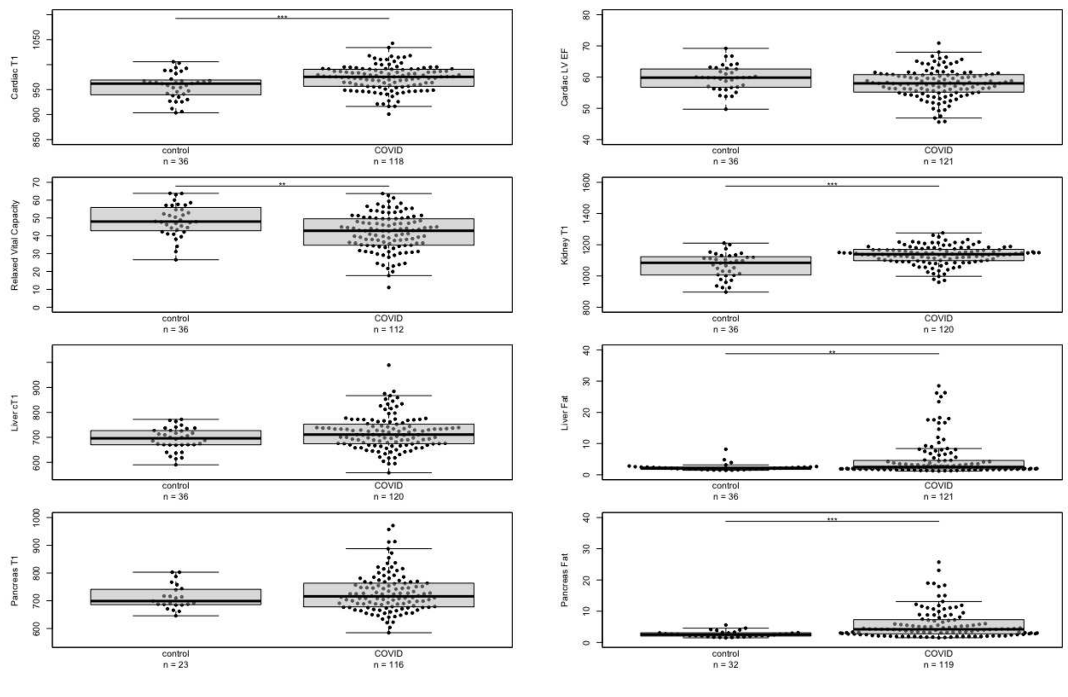

Dennis A, *et al. BMJ Open* 2021; 11:e048391. doi: 10.1136/bmjopen-2020-048391

Figure S2: Organ impairment in severe versus moderate post COVID syndrome (n=201)

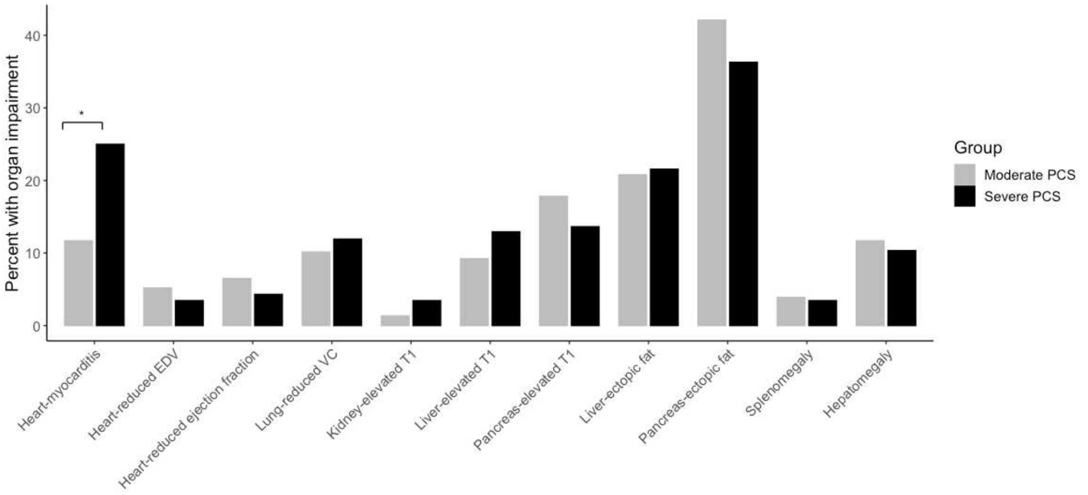

Table S1: Reference ranges for organ impairment, defined as a value that was greater than the mean plus 2 standard deviations of that from the control group for most; mean minus 2 standard deviations for left ventricular ejection fraction and lung fractional area difference for the 1.5T scans. For the 3T scans, this was the value as reported by Raman et al (2020).

	1.5T Reference range	3T reference range
Left ventricular ejection fraction (LVEF) (S4-S7)	≤ 51.5%	----
Increased end-diastolic volume (S4-S7)	≥ 264ml in men<br>≥ 206ml in women	----
Myocarditis (S4-S7)	≥ 1015 ms	≥ 1238ms
Deep breathing fractional area change*	≤ 31%	----
Liver volume (S8-S11)	≤ 1.93L	----
Liver fat (S8-S11)	≥ 4.8%	----
Liver inflammation (S8-S11)	≥ 784 ms	----
Pancreatic fat (S12-S13)	≥ 4.6%	----
Pancreatic inflammation (S12-13)	≥ 803ms	----
Renal Cortical T1(S14-S15)	≥ 1227ms	≥ 1652ms
Spleen volume(S16)	≤ 0.35L	----

** Our lung imaging protocol captured 2D dynamic imaging of the lungs as the patient breathes. We delineated the lungs at maximum inspiration and again at maximum expiration and take the difference to give a proxy of 'vital capacity', which correlates well with forced vital capacity (r = 0.61, P<.001) from spirometry. Given the measure was associated with body size, we divided the difference in maximum inspiration and expiration by maximum inspiration to give a normalised 'lung ejection fraction'. In order to assess whether an individual's 'lung ejection fraction' was abnormal, it was measured in 39 controls, characterising a healthy normal range of the mean +/- 2 standard deviations, with a lower score representing poorer lung health. 31% (0.31) was the lower limit for normal from our controls and therefore selected as the threshold for respiratory impairment.*

Table S2: Blood investigations in 201 low-risk individuals with post-COVID syndrome, sub-divided by those who were hospitalised versus those who were managed at home

Measurement	All	Managed at home	Hospitalised	p-value
Haemoglobin				
• Normal (130 - 170 g/L in men; 115 - 155 g/L in women)	170 (95.5%)	140 (95.9%)	30 (93.8%)	0.575
• Abnormal low (< 130 g/L in men; < 115 g/L in women)	5 (2.8%)	4 (2.7%)	1 (3.1%)	
• Abnormal high (> 170 g/L in men; > 155 g/L in women)	3 (1.7%)	2 (1.4%)	1 (3.1%)	
Haematocrit (HCT)				
• Normal (0.37 - 0.5 in men; 0.33 - 0.45 in women)	173 (97.2%)	142 (97.3%)	31 (96.9%)	0.386
• Abnormal low (< 0.37 in men; < 0.33 in women)	2 (1.1%)	1 (0.7%)	1 (3.1%)	
• Abnormal high (> 0.5 in men; > 0.45 in women)	3 (1.7%)	3 (2.1%)	0 (0%)	
Red cell count				
• Normal (4.4 - 5.8 x10^12/L in men; 3.95 - 5.15 x10^12/L in women)	170 (95.5%)	140 (95.9%)	30 (93.8%)	0.287
• Abnormal low (< 4.4 x10^12/L in men; < 3.95 x10^12/L in women)	5 (2.8%)	3 (2.1%)	2 (6.2%)	
• Abnormal high (> 5.8 x10^12/L in men; > 5.15 x10^12/L in women)	3 (1.7%)	3 (2.1%)	0 (0%)	
Mean cell volume (MCV)				
• Normal (80 - 99 fL)	174 (97.8%)	142 (97.3%)	32 (100%)	1
• Abnormal low (< 80 fL)	4 (2.2%)	4 (2.7%)	0 (0%)	
• Abnormal high (> 99 fL)	0 (0%)	0 (0%)	0 (0%)	
Mean corpuscular haemoglobin (MCH)				
• Normal (26 - 33.5 pg)	174 (97.8%)	143 (97.9%)	31 (96.9%)	0.249
• Abnormal low (< 26 pg)	3 (1.7%)	3 (2.1%)	0 (0%)	
• Abnormal high (> 33.5 pg)	1 (0.6%)	0 (0%)	1 (3.1%)	
Mean corpuscular haemoglobin concentration (MCHC)				
• Normal (300 - 350 g/L)	135 (75.8%)	109 (74.7%)	26 (81.2%)	0.501
• Abnormal low (< 300 g/L)	0 (0%)	0 (0%)	0 (0%)	
• Abnormal high (> 350 g/L)	43 (24.2%)	37 (25.3%)	6 (18.8%)	
Red cell distribution width (RDW)				
• Normal (11.5 - 15)	161 (91%)	129 (89%)	32 (100%)	0.218
• Abnormal low (< 11.5)	10 (5.6%)	10 (6.9%)	0 (0%)	
• Abnormal high (> 15)	6 (3.4%)	6 (4.1%)	0 (0%)	
Platelet count				
• Normal (150 - 400 x10^9/L)	166 (93.3%)	138 (94.5%)	28 (87.5%)	0.152
• Abnormal low (< 150 x10^9/L)	2 (1.1%)	2 (1.4%)	0 (0%)	
• Abnormal high (> 400 x10^9/L)	10 (5.6%)	6 (4.1%)	4 (12.5%)	
Mean platelet volume (MPV)				
• Normal (7 - 13 fL)	177 (99.4%)	145 (99.3%)	32 (100%)	1
• Abnormal low (< 7 fL)	0 (0%)	0 (0%)	0 (0%)	
• Abnormal high (> 13 fL)	1 (0.6%)	1 (0.7%)	0 (0%)	
White cell count				

• Normal (3 - 10 x10^9/L)	172 (96.6%)	140 (95.9%)	32 (100%)	0.593
• Abnormal low (< 3 x10^9/L)	0 (0%)	0 (0%)	0 (0%)	
• Abnormal high (> 10 x10^9/L)	6 (3.4%)	6 (4.1%)	0 (0%)	
Neutrophils				
• Normal (2 - 7.5 x10^9/L)	163 (91.6%)	133 (91.1%)	30 (93.8%)	1
• Abnormal low (< 2 x10^9/L)	12 (6.7%)	10 (6.8%)	2 (6.2%)	
• Abnormal high (> 7.5 x10^9/L)	3 (1.7%)	3 (2.1%)	0 (0%)	
Lymphocytes				
• Normal (1.2 - 3.65 x10^9/L)	161 (90.4%)	130 (89%)	31 (96.9%)	0.316
• Abnormal low (< 1.2 x10^9/L)	17 (9.6%)	16 (11%)	1 (3.1%)	
• Abnormal high (> 3.65 x10^9/L)	0 (0%)	0 (0%)	0 (0%)	
Monocytes				
• Normal (0.2 - 1 x10^9/L)	176 (98.9%)	144 (98.6%)	32 (100%)	1
• Abnormal low (< 0.2 x10^9/L)	1 (0.6%)	1 (0.7%)	0 (0%)	
• Abnormal high (> 1 x10^9/L)	1 (0.6%)	1 (0.7%)	0 (0%)	
Eosinophils				
• Normal (0 - 0.4 x10^9/L)	172 (96.6%)	141 (96.6%)	31 (96.9%)	1
• Abnormal low (< 0 x10^9/L)	0 (0%)	0 (0%)	0 (0%)	
• Abnormal high (> 0.4 x10^9/L)	6 (3.4%)	5 (3.4%)	1 (3.1%)	
Basophils				
• Normal (0 - 0.1 x10^9/L)	178 (100%)	146 (100%)	32 (100%)	N/A
• Abnormal low (< 0 x10^9/L)	0 (0%)	0 (0%)	0 (0%)	
• Abnormal high (> 0.1 x10^9/L)	0 (0%)	0 (0%)	0 (0%)	
Erythrocyte sedimentation rate (ESR)				
• Normal (1 - 20 mm/hr)	164 (91.1%)	136 (91.9%)	28 (87.5%)	0.491
• Abnormal low (< 1 mm/hr)	0 (0%)	0 (0%)	0 (0%)	
• Abnormal high (> 20 mm/hr)	16 (8.9%)	12 (8.1%)	4 (12.5%)	
Sodium				
• Normal (135 - 145 mmol/L)	173 (97.2%)	141 (96.6%)	32 (100%)	1
• Abnormal low (< 135 mmol/L)	4 (2.2%)	4 (2.7%)	0 (0%)	
• Abnormal high (> 145 mmol/L)	1 (0.6%)	1 (0.7%)	0 (0%)	
Potassium				
• Normal (3.5 - 5.1 mmol/L)	108 (62.1%)	87 (61.3%)	21 (65.6%)	0.692
• Abnormal low (< 3.5 mmol/L)	0 (0%)	0 (0%)	0 (0%)	
• Abnormal high (> 5.1 mmol/L)	66 (37.9%)	55 (38.7%)	11 (34.4%)	
Chloride				
• Normal (98 - 107 mmol/L)	171 (96.1%)	139 (95.2%)	32 (100%)	1
• Abnormal low (< 98 mmol/L)	4 (2.2%)	4 (2.7%)	0 (0%)	
• Abnormal high (> 107 mmol/L)	3 (1.7%)	3 (2.1%)	0 (0%)	
Bicarbonate				
• Normal (22 - 29 mmol/L)	150 (84.3%)	125 (85.6%)	25 (78.1%)	0.169
• Abnormal low (< 22 mmol/L)	18 (10.1%)	15 (10.3%)	3 (9.4%)	
• Abnormal high (> 29 mmol/L)	10 (5.6%)	6 (4.1%)	4 (12.5%)	
Urea				

• Normal (1.7 - 8.3 mmol/L)	178 (100%)	146 (100%)	32 (100%)	N/A
• Abnormal low (< 1.7 mmol/L)	0 (0%)	0 (0%)	0 (0%)	
• Abnormal high (> 8.3 mmol/L)	0 (0%)	0 (0%)	0 (0%)	
Creatinine				
• Normal (66 - 112 umol/L in men; 49 - 92 umol/L in women)	161 (90.4%)	134 (91.8%)	27 (84.4%)	0.219
• Abnormal low (< 66 umol/L in men; < 49 umol/L in women)	12 (6.7%)	9 (6.2%)	3 (9.4%)	
• Abnormal high (> 112 umol/L in men; > 92 umol/L in women)	5 (2.8%)	3 (2.1%)	2 (6.2%)	
Bilirubin				
• Normal (0 - 20 umol/L)	175 (98.3%)	144 (98.6%)	31 (96.9%)	0.45
• Abnormal low (< 0 umol/L)	0 (0%)	0 (0%)	0 (0%)	
• Abnormal high (> 20 umol/L)	3 (1.7%)	2 (1.4%)	1 (3.1%)	
Alkaline phosphatase				
• Normal (40 - 129 IU/L in men; 35 - 104 IU/L in women)	168 (94.4%)	137 (93.8%)	31 (96.9%)	0.161
• Abnormal low (< 40 IU/L in men; < 35 IU/L in women)	8 (4.5%)	8 (5.5%)	0 (0%)	
• Abnormal high (> 129 IU/L in men; > 104 IU/L in women)	2 (1.1%)	1 (0.7%)	1 (3.1%)	
Aspartate transferase				
• Normal (0 - 37 IU/L in men; 0 - 31 IU/L in women)	162 (93.1%)	133 (93.7%)	29 (90.6%)	0.464
• Abnormal low (< 0 IU/L in men; < 0 IU/L in women)	0 (0%)	0 (0%)	0 (0%)	
• Abnormal high (> 37 IU/L in men; > 31 IU/L in women)	12 (6.9%)	9 (6.3%)	3 (9.4%)	
Alanine transferase				
• Normal (10 - 50 IU/L in men; 10 - 35 IU/L in women)	151 (84.8%)	125 (85.6%)	26 (81.2%)	0.603
• Abnormal low (< 10 IU/L in men; < 10 IU/L in women)	2 (1.1%)	2 (1.4%)	0 (0%)	
• Abnormal high (> 50 IU/L in men; > 35 IU/L in women)	25 (14%)	19 (13%)	6 (18.8%)	
Lactate dehydrogenase (LDH)				
• Normal (135 - 225 IU/L in men; 135 - 214 IU/L in women)	142 (80.7%)	118 (81.9%)	24 (75%)	0.236
• Abnormal low (< 135 IU/L in men; < 135 IU/L in women)	5 (2.8%)	5 (3.5%)	0 (0%)	
• Abnormal high (> 225 IU/L in men; > 214 IU/L in women)	29 (16.5%)	21 (14.6%)	8 (25%)	
Creatinine kinase (CK)				
• Normal (38 - 204 IU/L in men; 26 - 140 IU/L in women)	163 (91.6%)	132 (90.4%)	31 (96.9%)	0.642
• Abnormal low (< 38 IU/L in men; < 26 IU/L in women)	2 (1.1%)	2 (1.4%)	0 (0%)	
• Abnormal high (> 204 IU/L in men; > 140 IU/L in women)	13 (7.3%)	12 (8.2%)	1 (3.1%)	
Gamma glutamyl transferase				
• Normal (10 - 71 IU/L in men; 6 - 42 IU/L in women)	165 (92.7%)	136 (93.2%)	29 (90.6%)	0.461
• Abnormal low (< 10 IU/L in men; < 6 IU/L in women)	4 (2.2%)	4 (2.7%)	0 (0%)	
• Abnormal high (> 71 IU/L in men; > 42 IU/L in women)	9 (5.1%)	6 (4.1%)	3 (9.4%)	
Total protein				
• Normal (63 - 83 g/L)	173 (97.2%)	143 (97.9%)	30 (93.8%)	0.22
• Abnormal low (< 63 g/L)	3 (1.7%)	2 (1.4%)	1 (3.1%)	
• Abnormal high (> 83 g/L)	2 (1.1%)	1 (0.7%)	1 (3.1%)	
Albumin				
• Normal (34 - 50 g/L)	167 (93.8%)	136 (93.2%)	31 (96.9%)	0.692
• Abnormal low (< 34 g/L)	0 (0%)	0 (0%)	0 (0%)	
• Abnormal high (> 50 g/L)	11 (6.2%)	10 (6.8%)	1 (3.1%)	
Globulin				

• Normal (19 - 35 g/L)	173 (97.2%)	142 (97.3%)	31 (96.9%)	0.386
• Abnormal low (< 19 g/L)	3 (1.7%)	3 (2.1%)	0 (0%)	
• Abnormal high (> 35 g/L)	2 (1.1%)	1 (0.7%)	1 (3.1%)	
Calcium				
• Normal (2.2 - 2.6 mmol/L)	172 (96.6%)	141 (96.6%)	31 (96.9%)	0.43
• Abnormal low (< 2.2 mmol/L)	2 (1.1%)	1 (0.7%)	1 (3.1%)	
• Abnormal high (> 2.6 mmol/L)	4 (2.2%)	4 (2.7%)	0 (0%)	
Magnesium				
• Normal (0.6 - 1 mmol/L)	176 (98.9%)	144 (98.6%)	32 (100%)	1
• Abnormal low (< 0.6 mmol/L)	1 (0.6%)	1 (0.7%)	0 (0%)	
• Abnormal high (> 1 mmol/L)	1 (0.6%)	1 (0.7%)	0 (0%)	
Phosphate				
• Normal (0.87 - 1.45 mmol/L)	150 (84.3%)	121 (82.9%)	29 (90.6%)	0.518
• Abnormal low (< 0.87 mmol/L)	23 (12.9%)	21 (14.4%)	2 (6.2%)	
• Abnormal high (> 1.45 mmol/L)	5 (2.8%)	4 (2.7%)	1 (3.1%)	
Uric acid				
• Normal (266 - 474 umol/L in men; 175 - 363 umol/L in women)	148 (83.1%)	124 (84.9%)	24 (75%)	0.067
• Abnormal low (< 266 umol/L in men; < 175 umol/L in women)	19 (10.7%)	16 (11%)	3 (9.4%)	
• Abnormal high (> 474 umol/L in men; > 363 umol/L in women)	11 (6.2%)	6 (4.1%)	5 (15.6%)	
Triglycerides				
• Normal (< 2.3 mmol/L)	10 (100%)	8 (100%)	2 (100%)	N/A
• Abnormal high (> 2.3 mmol/L)	0 (0%)	0 (0%)	0 (0%)	
Fasting triglycerides				
• Normal (< 2.3 mmol/L)	149 (88.7%)	128 (92.8%)	21 (70%)	0.002
• Abnormal high (> 2.3 mmol/L)	19 (11.3%)	10 (7.2%)	9 (30%)	
Cholesterol				
• Normal (< 5 mmol/L)	4 (40%)	3 (37.5%)	1 (50%)	1
• Abnormal high (> 5 mmol/L)	6 (60%)	5 (62.5%)	1 (50%)	
Fasting cholesterol				
• Normal (< 5 mmol/L)	98 (58.3%)	86 (62.3%)	12 (40%)	0.04
• Abnormal high (> 5 mmol/L)	70 (41.7%)	52 (37.7%)	18 (60%)	
HDL cholesterol				
• Normal (0.9 - 1.5 mmol/L in men; 1.2 - 1.7 mmol/L in women)	106 (59.6%)	87 (59.6%)	19 (59.4%)	0.075
• Abnormal low (< 0.9 mmol/L in men; < 1.2 mmol/L in women)	16 (9%)	10 (6.8%)	6 (18.8%)	
• Abnormal high (> 1.5 mmol/L in men; > 1.7 mmol/L in women)	56 (31.5%)	49 (33.6%)	7 (21.9%)	
LDL cholesterol				
• Normal (< 3 mmol/L)	113 (64.9%)	100 (69.4%)	13 (43.3%)	0.011
• Abnormal high (> 3 mmol/L)	61 (35.1%)	44 (30.6%)	17 (56.7%)	
Iron				
• Normal (10.6 - 28.3 umol/L in men; 6.6 - 26 umol/L in women)	164 (92.1%)	135 (92.5%)	29 (90.6%)	0.22
• Abnormal low (< 10.6 umol/L in men; < 6.6 umol/L in women)	4 (2.2%)	2 (1.4%)	2 (6.2%)	

Dennis A, *et al. BMJ Open* 2021; 11:e048391. doi: 10.1136/bmjopen-2020-048391

• Abnormal high (> 28.3 umol/L in men; > 26 umol/L in women)	10 (5.6%)	9 (6.2%)	1 (3.1%)	
Total iron binding capacity (TIBC)				
• Normal (41 - 77 umol/L)	172 (97.2%)	141 (97.2%)	31 (96.9%)	1
• Abnormal low (< 41 umol/L)	0 (0%)	0 (0%)	0 (0%)	
• Abnormal high (> 77 umol/L)	5 (2.8%)	4 (2.8%)	1 (3.1%)	
Transferrin saturation				
• Normal (20 - 55 %)	139 (78.5%)	120 (82.8%)	19 (59.4%)	0.011
• Abnormal low (< 20 %)	34 (19.2%)	22 (15.2%)	12 (37.5%)	
• Abnormal high (> 55 %)	4 (2.3%)	3 (2.1%)	1 (3.1%)	
High sensitivity CRP				
• Normal (0 - 5 mg/L)	146 (92.4%)	124 (93.9%)	22 (84.6%)	0.112
• Abnormal low (< 0 mg/L)	0 (0%)	0 (0%)	0 (0%)	
• Abnormal high (> 5 mg/L)	12 (7.6%)	8 (6.1%)	4 (15.4%)	

Table S3: Blood investigations in 201 low-risk individuals sub-divided by those with severe or moderate post-COVID syndrome (PCS)

Measurement	All	Moderate PCS	Severe PCS	p-value
Haemoglobin				
• Normal (130 - 170 g/L in men; 115 - 155 g/L in women)	166 (96%)	62 (96.9%)	104 (95.4%)	1
• Abnormal low (< 130 g/L in men; < 115 g/L in women)	4 (2.3%)	1 (1.6%)	3 (2.8%)	
• Abnormal high (> 170 g/L in men; > 155 g/L in women)	3 (1.7%)	1 (1.6%)	2 (1.8%)	
Haematocrit (HCT)				
• Normal (0.37 - 0.5 in men; 0.33 - 0.45 in women)	168 (97.1%)	64 (100%)	104 (95.4%)	0.274
• Abnormal low (< 0.37 in men; < 0.33 in women)	2 (1.2%)	0 (0%)	2 (1.8%)	
• Abnormal high (> 0.5 in men; > 0.45 in women)	3 (1.7%)	0 (0%)	3 (2.8%)	
Red cell count				
• Normal (4.4 - 5.8 x10^12/L in men; 3.95 - 5.15 x10^12/L in women)	167 (96.5%)	61 (95.3%)	106 (97.2%)	0.825
• Abnormal low (< 4.4 x10^12/L in men; < 3.95 x10^12/L in women)	4 (2.3%)	2 (3.1%)	2 (1.8%)	
• Abnormal high (> 5.8 x10^12/L in men; > 5.15 x10^12/L in women)	2 (1.2%)	1 (1.6%)	1 (0.9%)	
Mean cell volume (MCV)				
• Normal (80 - 99 fL)	170 (98.3%)	62 (96.9%)	108 (99.1%)	0.556
• Abnormal low (< 80 fL)	3 (1.7%)	2 (3.1%)	1 (0.9%)	
• Abnormal high (> 99 fL)	0 (0%)	0 (0%)	0 (0%)	
Mean corpuscular haemoglobin (MCH)				
• Normal (26 - 33.5 pg)	170 (98.3%)	61 (95.3%)	109 (100%)	0.049
• Abnormal low (< 26 pg)	2 (1.2%)	2 (3.1%)	0 (0%)	
• Abnormal high (> 33.5 pg)	1 (0.6%)	1 (1.6%)	0 (0%)	
Mean corpuscular haemoglobin concentration (MCHC)				
• Normal (300 - 350 g/L)	131 (75.7%)	53 (82.8%)	78 (71.6%)	0.103
• Abnormal low (< 300 g/L)	0 (0%)	0 (0%)	0 (0%)	
• Abnormal high (> 350 g/L)	42 (24.3%)	11 (17.2%)	31 (28.4%)	
Red cell distribution width (RDW)				
• Normal (11.5 - 15)	157 (91.3%)	59 (92.2%)	98 (90.7%)	0.339
• Abnormal low (< 11.5)	10 (5.8%)	2 (3.1%)	8 (7.4%)	
• Abnormal high (> 15)	5 (2.9%)	3 (4.7%)	2 (1.9%)	
Platelet count				
• Normal (150 - 400 x10^9/L)	161 (93.1%)	59 (92.2%)	102 (93.6%)	0.417
• Abnormal low (< 150 x10^9/L)	2 (1.2%)	0 (0%)	2 (1.8%)	
• Abnormal high (> 400 x10^9/L)	10 (5.8%)	5 (7.8%)	5 (4.6%)	
Mean platelet volume (MPV)				
• Normal (7 - 13 fL)	172 (99.4%)	64 (100%)	108 (99.1%)	1
• Abnormal low (< 7 fL)	0 (0%)	0 (0%)	0 (0%)	

Dennis A, *et al. BMJ Open* 2021; 11:e048391. doi: 10.1136/bmjopen-2020-048391

• Abnormal high (> 13 fL)	1 (0.6%)	0 (0%)	1 (0.9%)	
White cell count				
• Normal (3 - 10 x10^9/L)	167 (96.5%)	61 (95.3%)	106 (97.2%)	0.671
• Abnormal low (< 3 x10^9/L)	0 (0%)	0 (0%)	0 (0%)	
• Abnormal high (> 10 x10^9/L)	6 (3.5%)	3 (4.7%)	3 (2.8%)	
Neutrophils				
• Normal (2 - 7.5 x10^9/L)	159 (91.9%)	57 (89.1%)	102 (93.6%)	0.468
• Abnormal low (< 2 x10^9/L)	11 (6.4%)	5 (7.8%)	6 (5.5%)	
• Abnormal high (> 7.5 x10^9/L)	3 (1.7%)	2 (3.1%)	1 (0.9%)	
Lymphocytes				
• Normal (1.2 - 3.65 x10^9/L)	156 (90.2%)	56 (87.5%)	100 (91.7%)	0.43
• Abnormal low (< 1.2 x10^9/L)	17 (9.8%)	8 (12.5%)	9 (8.3%)	
• Abnormal high (> 3.65 x10^9/L)	0 (0%)	0 (0%)	0 (0%)	
Monocytes				
• Normal (0.2 - 1 x10^9/L)	171 (98.8%)	63 (98.4%)	108 (99.1%)	0.604
• Abnormal low (< 0.2 x10^9/L)	1 (0.6%)	0 (0%)	1 (0.9%)	
• Abnormal high (> 1 x10^9/L)	1 (0.6%)	1 (1.6%)	0 (0%)	
Eosinophils				
• Normal (0 - 0.4 x10^9/L)	167 (96.5%)	63 (98.4%)	104 (95.4%)	0.415
• Abnormal low (< 0 x10^9/L)	0 (0%)	0 (0%)	0 (0%)	
• Abnormal high (> 0.4 x10^9/L)	6 (3.5%)	1 (1.6%)	5 (4.6%)	
Basophils				
• Normal (0 - 0.1 x10^9/L)	173 (100%)	64 (100%)	109 (100%)	N/A
• Abnormal low (< 0 x10^9/L)	0 (0%)	0 (0%)	0 (0%)	
• Abnormal high (> 0.1 x10^9/L)	0 (0%)	0 (0%)	0 (0%)	
Erythrocyte sedimentation rate (ESR)				
• Normal (1 - 20 mm/hr)	160 (91.4%)	62 (93.9%)	98 (89.9%)	0.416
• Abnormal low (< 1 mm/hr)	0 (0%)	0 (0%)	0 (0%)	
• Abnormal high (> 20 mm/hr)	15 (8.6%)	4 (6.1%)	11 (10.1%)	
Sodium				
• Normal (135 - 145 mmol/L)	168 (97.1%)	63 (98.4%)	105 (96.3%)	1
• Abnormal low (< 135 mmol/L)	4 (2.3%)	1 (1.6%)	3 (2.8%)	
• Abnormal high (> 145 mmol/L)	1 (0.6%)	0 (0%)	1 (0.9%)	
Potassium				
• Normal (3.5 - 5.1 mmol/L)	105 (62.1%)	35 (56.5%)	70 (65.4%)	0.255
• Abnormal low (< 3.5 mmol/L)	0 (0%)	0 (0%)	0 (0%)	
• Abnormal high (> 5.1 mmol/L)	64 (37.9%)	27 (43.5%)	37 (34.6%)	
Chloride				
• Normal (98 - 107 mmol/L)	166 (96%)	62 (96.9%)	104 (95.4%)	1
• Abnormal low (< 98 mmol/L)	4 (2.3%)	1 (1.6%)	3 (2.8%)	
• Abnormal high (> 107 mmol/L)	3 (1.7%)	1 (1.6%)	2 (1.8%)	

Bicarbonate				
• Normal (22 - 29 mmol/L)	147 (85%)	55 (85.9%)	92 (84.4%)	0.946
• Abnormal low (< 22 mmol/L)	16 (9.2%)	6 (9.4%)	10 (9.2%)	
• Abnormal high (> 29 mmol/L)	10 (5.8%)	3 (4.7%)	7 (6.4%)	
Urea				
• Normal (1.7 - 8.3 mmol/L)	173 (100%)	64 (100%)	109 (100%)	N/A
• Abnormal low (< 1.7 mmol/L)	0 (0%)	0 (0%)	0 (0%)	
• Abnormal high (> 8.3 mmol/L)	0 (0%)	0 (0%)	0 (0%)	
Creatinine				
• Normal (66 - 112 umol/L in men; 49 - 92 umol/L in women)	156 (90.2%)	59 (92.2%)	97 (89%)	0.705
• Abnormal low (< 66 umol/L in men; < 49 umol/L in women)	12 (6.9%)	3 (4.7%)	9 (8.3%)	
• Abnormal high (> 112 umol/L in men; > 92 umol/L in women)	5 (2.9%)	2 (3.1%)	3 (2.8%)	
Bilirubin				
• Normal (0 - 20 umol/L)	170 (98.3%)	63 (98.4%)	107 (98.2%)	1
• Abnormal low (< 0 umol/L)	0 (0%)	0 (0%)	0 (0%)	
• Abnormal high (> 20 umol/L)	3 (1.7%)	1 (1.6%)	2 (1.8%)	
Alkaline phosphatase				
• Normal (40 - 129 IU/L in men; 35 - 104 IU/L in women)	164 (94.8%)	59 (92.2%)	105 (96.3%)	0.185
• Abnormal low (< 40 IU/L in men; < 35 IU/L in women)	7 (4%)	3 (4.7%)	4 (3.7%)	
• Abnormal high (> 129 IU/L in men; > 104 IU/L in women)	2 (1.2%)	2 (3.1%)	0 (0%)	
Aspartate transferase				
• Normal (0 - 37 IU/L in men; 0 - 31 IU/L in women)	157 (92.9%)	59 (93.7%)	98 (92.5%)	1
• Abnormal low (< 0 IU/L in men; < 0 IU/L in women)	0 (0%)	0 (0%)	0 (0%)	
• Abnormal high (> 37 IU/L in men; > 31 IU/L in women)	12 (7.1%)	4 (6.3%)	8 (7.5%)	
Alanine transferase				
• Normal (10 - 50 IU/L in men; 10 - 35 IU/L in women)	146 (84.4%)	56 (87.5%)	90 (82.6%)	0.512
• Abnormal low (< 10 IU/L in men; < 10 IU/L in women)	2 (1.2%)	1 (1.6%)	1 (0.9%)	
• Abnormal high (> 50 IU/L in men; > 35 IU/L in women)	25 (14.5%)	7 (10.9%)	18 (16.5%)	
Lactate dehydrogenase (LDH)				
• Normal (135 - 225 IU/L in men; 135 - 214 IU/L in women)	137 (80.1%)	51 (81%)	86 (79.6%)	0.24
• Abnormal low (< 135 IU/L in men; < 135 IU/L in women)	5 (2.9%)	0 (0%)	5 (4.6%)	
• Abnormal high (> 225 IU/L in men; > 214 IU/L in women)	29 (17%)	12 (19%)	17 (15.7%)	
Creatinine kinase (CK)				
• Normal (38 - 204 IU/L in men; 26 - 140 IU/L in women)	159 (91.9%)	56 (87.5%)	103 (94.5%)	0.28
• Abnormal low (< 38 IU/L in men; < 26 IU/L in women)	2 (1.2%)	1 (1.6%)	1 (0.9%)	
• Abnormal high (> 204 IU/L in men; > 140 IU/L in women)	12 (6.9%)	7 (10.9%)	5 (4.6%)	
Gamma glutamyl transferase				
• Normal (10 - 71 IU/L in men; 6 - 42 IU/L in women)	161 (93.1%)	60 (93.8%)	101 (92.7%)	0.426
• Abnormal low (< 10 IU/L in men; < 6 IU/L in women)	3 (1.7%)	0 (0%)	3 (2.8%)	
• Abnormal high (> 71 IU/L in men; > 42 IU/L in women)	9 (5.2%)	4 (6.2%)	5 (4.6%)	
Total protein				
• Normal (63 - 83 g/L)	168 (97.1%)	63 (98.4%)	105 (96.3%)	0.792

• Abnormal low (< 63 g/L)	3 (1.7%)	1 (1.6%)	2 (1.8%)	
• Abnormal high (> 83 g/L)	2 (1.2%)	0 (0%)	2 (1.8%)	
Albumin				
• Normal (34 - 50 g/L)	162 (93.6%)	59 (92.2%)	103 (94.5%)	0.538
• Abnormal low (< 34 g/L)	0 (0%)	0 (0%)	0 (0%)	
• Abnormal high (> 50 g/L)	11 (6.4%)	5 (7.8%)	6 (5.5%)	
Globulin				
• Normal (19 - 35 g/L)	168 (97.1%)	61 (95.3%)	107 (98.2%)	0.616
• Abnormal low (< 19 g/L)	3 (1.7%)	2 (3.1%)	1 (0.9%)	
• Abnormal high (> 35 g/L)	2 (1.2%)	1 (1.6%)	1 (0.9%)	
Calcium				
• Normal (2.2 - 2.6 mmol/L)	167 (96.5%)	62 (96.9%)	105 (96.3%)	0.525
• Abnormal low (< 2.2 mmol/L)	2 (1.2%)	0 (0%)	2 (1.8%)	
• Abnormal high (> 2.6 mmol/L)	4 (2.3%)	2 (3.1%)	2 (1.8%)	
Magnesium				
• Normal (0.6 - 1 mmol/L)	171 (98.8%)	63 (98.4%)	108 (99.1%)	0.604
• Abnormal low (< 0.6 mmol/L)	1 (0.6%)	1 (1.6%)	0 (0%)	
• Abnormal high (> 1 mmol/L)	1 (0.6%)	0 (0%)	1 (0.9%)	
Phosphate				
• Normal (0.87 - 1.45 mmol/L)	145 (83.8%)	55 (85.9%)	90 (82.6%)	0.824
• Abnormal low (< 0.87 mmol/L)	23 (13.3%)	8 (12.5%)	15 (13.8%)	
• Abnormal high (> 1.45 mmol/L)	5 (2.9%)	1 (1.6%)	4 (3.7%)	
Uric acid				
• Normal (266 - 474 umol/L in men; 175 - 363 umol/L in women)	145 (83.8%)	53 (82.8%)	92 (84.4%)	0.804
• Abnormal low (< 266 umol/L in men; < 175 umol/L in women)	18 (10.4%)	8 (12.5%)	10 (9.2%)	
• Abnormal high (> 474 umol/L in men; > 363 umol/L in women)	10 (5.8%)	3 (4.7%)	7 (6.4%)	
Triglycerides				
• Normal (< 2.3 mmol/L)	10 (100%)	6 (100%)	4 (100%)	N/A
• Abnormal high (> 2.3 mmol/L)	0 (0%)	0 (0%)	0 (0%)	
Fasting triglycerides				
• Normal (< 2.3 mmol/L)	144 (88.3%)	52 (89.7%)	92 (87.6%)	0.802
• Abnormal high (> 2.3 mmol/L)	19 (11.7%)	6 (10.3%)	13 (12.4%)	
Cholesterol				
• Normal (< 5 mmol/L)	4 (40%)	3 (50%)	1 (25%)	0.571
• Abnormal high (> 5 mmol/L)	6 (60%)	3 (50%)	3 (75%)	
Fasting cholesterol				
• Normal (< 5 mmol/L)	96 (58.9%)	39 (67.2%)	57 (54.3%)	0.135
• Abnormal high (> 5 mmol/L)	67 (41.1%)	19 (32.8%)	48 (45.7%)	
HDL cholesterol				
• Normal (0.9 - 1.5 mmol/L in men; 1.2 - 1.7 mmol/L in women)	103 (59.5%)	38 (59.4%)	65 (59.6%)	0.539
• Abnormal low (< 0.9 mmol/L in men; < 1.2 mmol/L in women)	16 (9.2%)	4 (6.2%)	12 (11%)	

• Abnormal high (> 1.5 mmol/L in men; > 1.7 mmol/L in women)	54 (31.2%)	22 (34.4%)	32 (29.4%)	
LDL cholesterol				
• Normal (< 3 mmol/L)	111 (65.7%)	45 (72.6%)	66 (61.7%)	0.18
• Abnormal high (> 3 mmol/L)	58 (34.3%)	17 (27.4%)	41 (38.3%)	
Iron				
• Normal (10.6 - 28.3 umol/L in men; 6.6 - 26 umol/L in women)	160 (92.5%)	57 (89.1%)	103 (94.5%)	0.337
• Abnormal low (< 10.6 umol/L in men; < 6.6 umol/L in women)	3 (1.7%)	2 (3.1%)	1 (0.9%)	
• Abnormal high (> 28.3 umol/L in men; > 26 umol/L in women)	10 (5.8%)	5 (7.8%)	5 (4.6%)	
Total iron binding capacity (TIBC)				
• Normal (41 - 77 umol/L)	167 (97.1%)	60 (93.8%)	107 (99.1%)	0.064
• Abnormal low (< 41 umol/L)	0 (0%)	0 (0%)	0 (0%)	
• Abnormal high (> 77 umol/L)	5 (2.9%)	4 (6.2%)	1 (0.9%)	
Transferrin saturation				
• Normal (20 - 55 %)	135 (78.5%)	50 (78.1%)	85 (78.7%)	0.283
• Abnormal low (< 20 %)	33 (19.2%)	11 (17.2%)	22 (20.4%)	
• Abnormal high (> 55 %)	4 (2.3%)	3 (4.7%)	1 (0.9%)	
High sensitivity CRP				
• Normal (0 - 5 mg/L)	141 (92.2%)	50 (96.2%)	91 (90.1%)	0.223
• Abnormal low (< 0 mg/L)	0 (0%)	0 (0%)	0 (0%)	
• Abnormal high (> 5 mg/L)	12 (7.8%)	2 (3.8%)	10 (9.9%)	

Dennis A, *et al. BMJ Open* 2021; 11:e048391. doi: 10.1136/bmjopen-2020-048391
