# Supplementary material for: Multiorgan impairment in low-risk individuals with post-COVID-19 syndrome: a prospective, community-based study
Source: BMJ Open. 2021 Mar 30;11(3):e048391. doi: 10.1136/bmjopen-2020-048391 (PMC8727683; doi:10.1136/bmjopen-2020-048391)
Supplement: Author's manuscript [file bmjopen-2020-048391.draft_revisions.pdf]

BMJ Open

BMJ Open is committed to open peer review. As part of this commitment we make the peer review history of every article we publish publicly available.

When an article is published we post the peer reviewers' comments and the authors' responses online. We also post the versions of the paper that were used during peer review. These are the versions that the peer review comments apply to.

The versions of the paper that follow are the versions that were submitted during the peer review process. They are not the versions of record or the final published versions. They should not be cited or distributed as the published version of this manuscript.

BMJ Open is an open access journal and the full, final, typeset and author-corrected version of record of the manuscript is available on our site with no access controls, subscription charges or pay-per-view fees (http://bmjopen.bmj.com).

If you have any questions on BMJ Open's open peer review process please email info.bmjopen@bmj.com

BMJ Open

Multi-organ impairment in low-risk individuals with post-COVID syndrome

Journal:	*BMJ Open*
Manuscript ID	bmjopen-2020-048391
Article Type:	Original research
Date Submitted by the Author:	26-Dec-2020
Complete List of Authors:	Dennis, Andrea; Perspectum Diagnostics Ltd, Innovation<br>Wamil, Malgorzata ; Great Western Hospital Foundation NHS Trust, Department of Cardiology<br>Alberts, Johann; Alliance Medical Limited<br>Oben, Jude; Guy's and St Thomas' NHS Foundation Trust, Department of Gastroenterology<br>Cuthbertson, Daniel; University of Liverpool, Institute of Cardiovascular and Metabolic Medicine<br>Wootton, Dan; University of Liverpool Institute of Infection and Global Health; Liverpool University Hospitals NHS Foundation Trust, Department of Respiratory Research<br>Crooks, Michael; Hull and East Yorkshire Hospitals NHS Trust, Department of Respiratory Medicine<br>Gabbay, Mark; University of Liverpool, 12Institute of Population Health Sciences<br>Brady, MIchael; Perspectum Diagnostics Ltd<br>Hishmeh, Lyth; London<br>Attree, Emily; London<br>Heightman, Melissa; University College London Hospitals NHS Foundation Trust, Department of Medicine<br>Banerjee, Rajarshi; Perspectum Diagnostics Ltd<br>Banerjee, Amitava; University College London, Institute of Health Informatics
Keywords:	COVID-19, EPIDEMIOLOGY, Health policy < HEALTH SERVICES ADMINISTRATION & MANAGEMENT, Public health < INFECTIOUS DISEASES, PUBLIC HEALTH

SCHOLARONE™
Manuscripts

I, the Submitting Author has the right to grant and does grant on behalf of all authors of the Work (as defined in the below author licence), an exclusive licence and/or a non-exclusive licence for contributions from authors who are: i) UK Crown employees; ii) where BMJ has agreed a CC-BY licence shall apply, and/or iii) in accordance with the terms applicable for US Federal Government officers or employees acting as part of their official duties; on a worldwide, perpetual, irrevocable, royalty-free basis to BMJ Publishing Group Ltd ("BMJ") its licensees and where the relevant Journal is co-owned by BMJ to the co-owners of the Journal, to publish the Work in this journal and any other BMJ products and to exploit all rights, as set out in our licence.

The Submitting Author accepts and understands that any supply made under these terms is made by BMJ to the Submitting Author unless you are acting as an employee on behalf of your employer or a postgraduate student of an affiliated institution which is paying any applicable article publishing charge ("APC") for Open Access articles. Where the Submitting Author wishes to make the Work available on an Open Access basis (and intends to pay the relevant APC), the terms of reuse of such Open Access shall be governed by a Creative Commons licence – details of these licences and which Creative Commons licence will apply to this Work are set out in our licence referred to above.

Other than as permitted in any relevant BMJ Author's Self Archiving Policies, I confirm this Work has not been accepted for publication elsewhere, is not being considered for publication elsewhere and does not duplicate material already published. I confirm all authors consent to publication of this Work and authorise the granting of this licence.

For peer review only

Multi-organ impairment in low-risk individuals with post-COVID syndrome

Dennis A, Wamil M, Alberts J, Oben J, Cuthbertson D, Wootton D, Crooks M, Gabbay M, Brady M, Hishmeh L, Attree E, Heightman M, Banerjee R*, Banerjee A*

Andrea Dennis PhD[1], *Head of Biomarker Science, Perspectum*
andrea.dennis@perspectum.com

Malgorzata Wamil PhD[2, 3] *Consultant Cardiologist* gosia.wamil@googlemail.com

Johann Alberts MBBCh[4], *Medical Director* jalberts@alliance.co.uk

Jude Oben PhD[5, 6], *Consultant Gastroenterologist/Hepatologist and Reader in Experimental Hepatology* jude.1.oben@kcl.ac.uk

Daniel Cuthbertson PhD[7 ,8], *Professor of Diabetes and Consultant Physician*
dan.cuthbertson@liverpool.ac.uk

Daniel Wootton PhD[8, 9], *Senior Fellow and Consultant Respiratory Physician*
D.G.Wootton@liverpool.ac.uk

Michael Crooks PhD [10, 11], *Senior Clinical Lecturer in Respiratory Medicine and Consultant Respiratory Physician* michael.crooks@hey.nhs.uk

Mark Gabbay PhD[12], *Professor of General Practice and General Practititoner*
M.B.Gabbay@liverpool.ac.uk

Michael Brady PhD[1, 13,] Professor of Oncological Imaging and Chair, Perspectum
Michael.Brady@perspectum.com

Lyth Hishmeh BSc[14], Member, Long COVID SOS lythb7@hotmail.com

Emily Attree MBBS[15], *salaried general practitioner and Founder,* UKDoctors#Longcovid
emily.attree@nhs.net

Melissa Heightman PhD[16], *Consultant Respiratory Physician* melissa.heightman1@nhs.net

Rajarshi Banerjee DPhil*[1,3], *Honorary Consultant Physician and Chief Executive, Perspectum* rajarshi.banerjee@perspectum.com

Amitava Banerjee DPhil*[16,17,18], *Associate Professor of Clinical Data Science and Honorary Consultant Cardiologist* ami.banerjee@ucl.ac.uk

On behalf of the COVERSCAN study investigators (listed at the end of manuscript)

[1]*Perspectum, 5520 John Smith Drive, Oxford, OX4 2LL, UK*

[2]*Department of Cardiology, Great Western Hospitals NHS Foundation Trust*

[3]*Nuffield Department of Medicine, Oxford University Hospitals NHS Foundation Trust*

[4]*Alliance Medical Limited, Iceni Centre, Warwick Technology Park, Warwick, CV34 6DA*

[5]*Department of Gastroenterology, Guy's and St Thomas' NHS Foundation Trust, London*

For peer review only

[6] *Institute for Liver and Digestive Health, University College London*
[7]*Institute of Cardiovascular and Metabolic Medicine, University of Liverpool*
[8]*Department of Medicine, Liverpool University Hospitals NHS Foundation Trust, Liverpool*
[9]*Institute of Infection & Global Health, University of Liverpool*
[10]*Institute of Clinical and Applied Health Research, University of Hull*
[11]*Department of Respiratory Medicine, Hull and East Yorkshire Hospitals NHS Trust, Hull.*
[12]*Institute of Population Health Sciences, University of Liverpool*
[13]*Department of Oncology, University of Oxford, Oxford.*
[14]*Long COVID SOS, UK*
[15]*UKDoctors#Longcovid, London*
[16]*Department of Medicine, University College London Hospitals NHS Trust, 235 Euston Road, London, UK*
[17]*Institute of Health Informatics, University College London, 222 Euston Road, London, UK*
[18]*Barts Health NHS Trust, The Royal London Hospital, Whitechapel Rd, London, UK*
*joint senior author
Corresponding authors: ami.banerjee@ucl.ac.uk; rajarshi.banerjee@perspectum.com

Article summary

Strengths and limitations of this study

- This is an ongoing, prospective, longitudinal COVID-19 recovery study with biochemical and imaging characterisation of organ function, starting in April before recognition of "long-COVID", proper testing availability and prospective COVID-19-related research.
- We included comparison with healthy, age-matched controls, although not matched for sex or baseline comorbidities.
- We did not explore different controls, e.g. individuals with post-flu symptoms, COVID-19 without symptoms or from general clinics.
- The study population was not ethnically diverse, despite disproportionate COVID-19 impact in non-white individuals.

For peer review only - http://bmjopen.bmj.com/site/about/guidelines.xhtml

- To limit interaction and exposure between trial team and patients, pulse oximetry, spirometry, MRI assessment of brain and muscle function were not included from the outset.

Key Message

In a cohort of individuals at low-risk of COVID-19 mortality, who contracted low-severity COVID-19, but remained symptomatic, there was evidence of impairment in the pancreas, heart, lung, kidneys and liver four months after the onset of COVID-19. Organ impairment was associated with higher levels of symptoms, in particular cardiac impairment. Our research suggests ‘long-COVID’ symptoms may, in some cases be explained by subclinical organ impairment. Longitudinal studies combining symptom trajectory and assessment (clinical, biochemical, physiological and imaging) will enable us to characterise the basis for full and complete recovery post-COVID, longer-term complications, ongoing symptoms, and effective pathways of care.

For peer review only

Summary box

What is already known on this topic

- There are no prior studies of medium- or long-term multi-organ impairment due to COVID-19.
- Prior studies have focused on acute phase of illness, hospitalised patients and "high-risk" individuals based on age and underlying conditions.
- Without longer-term data including lower risk individuals, full impact of the pandemic cannot be assessed and health system responses cannot be planned.

What this study adds

- Among 201 individuals at low risk of COVID-19 mortality, four months following SARS-CoV-2 infection, 42% had ≥10 symptoms, and 70% had mild impairment in at least one organ.
- Follow-up of multi-organ function may be necessary in individuals with post-COVID syndrome, and management of underlying conditions should be prioritised before and after infection.
- The best prevention strategy for post COVID syndrome is suppression of the infection rate.

review only

For peer review only

Abstract

Objective: To assess medium-term organ impairment in symptomatic individuals following recovery from acute SARS-CoV2 infection.

Design: Baseline findings from a prospective, observational cohort study.

Setting: Community-based individuals from two UK centres between 1 April and 14 September 2020.

For peer review only - http://bmjopen.bmj.com/site/about/guidelines.xhtml

Participants: Individuals ≥18 years with persistent symptoms following recovery from acute SARS-CoV-2 infection; and age-matched healthy controls.

Intervention: Assessment of symptoms by standardised questionnaires (EQ-5D-5L, Dyspnoea-12) and organ-specific metrics by biochemical assessment and quantitative magnetic resonance imaging (MRI).

Main outcome measures: Severe post-COVID syndrome defined as ongoing respiratory symptoms and/or moderate functional impairment in activities of daily living. Single and multi-organ impairment (heart, lungs, kidneys, liver, pancreas, spleen) by consensus definitions at baseline investigation.

Results: 201 individuals (mean age 45, range 21-71) years, 71% female, 88% white, 32% healthcare workers) completed baseline assessment (median 141 days following SARS-CoV-2 infection, IQR 110-162). The study population was low-risk for COVID-19 mortality (obesity: 20%, hypertension: 7%; type 2 diabetes: 2%; heart disease: 5%), with only 19% hospitalised with COVID-19. 42% of individuals had ten or more symptoms, and 60% had severe post-COVID syndrome. Fatigue (98%), muscle aches (87%), breathlessness (88%) and headaches (83%) were most frequently reported. Mild organ impairment was present in heart (26%), lungs (11%), kidneys (4%), liver (28%), pancreas (40%), and spleen (4%), with single and multi-organ impairment in 70% and 29% respectively. Hospitalisation was associated with older age ($p=0.001$), non-white ethnicity ($p=0.016$), increased liver volume ($p<0.0001$), pancreatic inflammation ($p<0.01$), and fat accumulation in the liver ($p<0.05$) and pancreas ($p<0.01$). Severe post-COVID syndrome was associated with radiological evidence of cardiac damage (myocarditis) ($p<0.05$).

For peer review only

Conclusions: In individuals at low risk of COVID-19 mortality with ongoing symptoms, 70% have impairment in one or more organs four months after initial COVID-19 symptoms, with implications for healthcare and public health, which have assumed low risk in young people with no comorbidities.

Study registration: https://clinicaltrials.gov/ct2/show/NCT04369807

For peer review only

Introduction

Early in the COVID-19 pandemic, research and clinical practice focused on pulmonary manifestations[1]. There is increasing evidence for direct multi-organ effects[2-7], as well as indirect effects on other organ systems and disease processes, such as cardiovascular diseases and cancers, through changes in healthcare delivery and patient behaviours[8–10]. The clear long-term impact on individuals and health systems underlines the urgent need for a whole body approach with assessment of all major organ systems following SARS-CoV-2 (Severe acute respiratory syndrome-coronavirus 2) infection.

COVID-19 is the convergence of an infectious disease, under-treated non-communicable diseases and social determinants of health, described as a “syndemic”[11]. Pre-existing non-communicable diseases and risk factors predict poor COVID-19 outcomes, whether intensive care admission or mortality[10]. Research has emphasised acute SARS-CoV-2 infection, hospitalised individuals, and COVID-19 mortality[12–14], which is likely to under-estimate the

For peer review only - http://bmjopen.bmj.com/site/about/guidelines.xhtml

true burden of COVID-19-related disease. Among those surviving acute infection, 10% report persistent symptoms for 12 weeks or longer after initial infection ("long-COVID", or "post COVID syndrome", PCS)[15]. However, PCS is yet to be fully defined[16-19]. Neither severity of symptoms, nor medium- and long-term pathophysiology across organ systems, nor the appropriate control populations are understood.

UK government policies have emphasised excess mortality risk in moderate- and high-risk conditions, including "shielding"[10] and commissioning of a risk calculator to identify those at highest risk of COVID-19 severity and mortality[20]. These policies assume that younger individuals without apparent underlying conditions are at low risk. However, unlike symptoms following critical illness[21] or acute phase of other coronavirus infections[22], symptoms in PCS are commonly reported in individuals with low COVID-19 mortality risk, e.g. female, young and no chronic co-morbidities[13]. The potential scale of PCS in "lower-risk" individuals, representing up to 80% of the population[3], necessitates urgent policies across countries to monitor[23], treat[18] and pay[24] for long-term implications of COVID-19, and to mitigate impact on healthcare utilisation and economies.

Therefore, in a pragmatic, prospective cohort of individuals with persistent symptoms following recovery from acute SARS-CoV2 infection and at low risk of COVID-19 mortality, we investigated: (i) multi-organ impairment compared with healthy, age-matched controls; and (ii) associations between symptoms and multi-organ impairment; and (iii) impact of hospitalisation and severity of symptoms.

For peer review only

Methods

Patient population and study design

In an ongoing, prospective study, participants were recruited either in response to advertising about the study or clinical specialist referral at two UK research imaging sites (Perspectum, Oxford and Mayo Clinic Healthcare, London) between 1 April 2020 and 14 September 2020, completing baseline assessment by 14 September 2020 (**Figure 1**). Participants were eligible for enrolment with laboratory confirmed SAR-COV2 infection (tested SARS-CoV-2-positive by oro/nasopharyngeal swab by reverse-transcriptase-polymerase-chain reaction (n=62), a positive antibody test (n=63), or with strong clinical suspicion of infection with typical symptoms/signs and assessed as highly likely to have COVID-19 by two independent clinicians (n=73)). Exclusion criteria were: symptoms of active respiratory viral infection (temperature >37.8°C or three or more episodes of coughing in 24 hours); hospital discharge in the last 7 days; and contraindications to MRI, including: implanted pacemakers, defibrillators, other metallic implanted devices and claustrophobia. All participants gave written informed consent.

To assess the burden of multi-organ involvement after SARS-CoV2 infection

Assessment included patient-reported validated questionnaires (quality of life, EQ-5D-5L[25], and dyspnoea-12[26]), fasting biochemical investigations (listed in **Supplementary Methods**) and multi-organ MRI. We selected MRI as the imaging modality (as in UK Biobank) due to: (1) safety (no radiation exposure, no need for intravenous contrast, and minimal contact with the radiographer); (2) quantitative reproducibility (>95% acquisition and image processing success rate); (3) capacity for information sharing (digital data repository for independent analysis and research); and (4) rapid scalability (35-minute scan to phenotype lung, heart, kidney, liver, pancreas and spleen). PCS was classified as "severe" (defined as persistent

For peer review only - http://bmjopen.bmj.com/site/about/guidelines.xhtml

breathlessness, ≥10 on the dyspnoea-12 score, or reported moderate or greater problems with usual activities on EQ-5D-5L), or “moderate”. These thresholds were selected as the dyspnoea-12 has been correlated with the MRC dyspnoea grade, where level 3 warrants referral to rehabilitation services[26], and with EQ-5D-5L, less than 8% of the general population report moderate or greater problems with usual activities[27].

Magnetic Resonance Image Analysis

Multi-organ MRI data were collected at both study sites (Oxford: MAGNETOM Aera 1.5T, Mayo Healthcare London: MAGNETOM Vida 3T; both from Siemens Healthcare, Erlangen, Germany). The COVERSCAN multi-parametic MRI assessment typically required 35 minutes per patient, including lungs, heart, liver, pancreas, kidneys and spleen by standardised methodology (**Supplementary methods**). In brief, we assessed inflammation of the heart, kidneys, liver and pancreas with quantitative T1-relaxation mapping, lung function was characterised with a dynamic structural T2-weighted lung scan measuring relaxed vital capacity, ectopic fat accumulation in the liver and pancreas from proton density fat fraction and volume of the liver and spleen measured from T1-weighted structural scan.

Definition of organ impairment

To determine impairment for each organ, we compared MRI-derived measurements from heart, lungs, kidney, liver, pancreas and spleen with reference ranges (**Table S1**), which were established as mean +/- 2 standard deviations from the healthy, age-matched control subjects (n=36)(**Figure S1**), and validated by scoping literature review[28]. We defined organ impairment if quantitative T1 mapping was outside reference ranges for heart, kidney, liver and pancreas, reduced pulmonary dynamic measurements in the lungs or there was evidence of hepatomegaly, splenomegaly or ectopic fat accumulation.

Patient and public involvement and engagement

For peer review only - http://bmjopen.bmj.com/site/about/guidelines.xhtml

Patients and public have directly, and indirectly, informed our research, from design to dissemination, with regular updates and webinars, including Q&A sessions with patients. Several clinician co-authors were indirectly informed by their patients in the COVERSCAN study (RB, AB) or PCS clinics (DW, MH, MC), who are members of organisations, such as Long Covid SOS (e.g. LH) and UKDoctors#Longcovid (e.g. EA). LH and EA have been involved in the research, interpretation of results, understanding implications of our results, and providing critical feedback for the manuscript.

Statistical analysis

We performed all analyses using R version 3.6.1, using descriptive statistics to summarise baseline characteristics, and considering a p-value less than 0.05 as statistically significant. Mean and standard deviation (SD) were used for normally distributed-continuous, median with interquartile range (IQR) for non-normally distributed, and frequency and percentage for categorical variables, respectively. We compared mean differences in quantitative organ metrics for hospitalised versus not hospitalised and moderate versus severe PCS using Kruskal-Wallis test (Fisher's exact test for differences in binary outcomes). We defined multi-organ impairment as ≥2 organs with metrics outside the reference range. We investigated associations between multi-organ impairment and (i) being hospitalised and (ii) severe PCS with multivariate logistic regression models, adjusting for age, sex and BMI. Associations between organ impairment and symptoms were visually assessed using heat map techniques. For group-wise comparison for absolute values between cases and healthy controls, we used Kruskal-Wallis test.

For peer review only

For peer review only - http://bmjopen.bmj.com/site/about/guidelines.xhtml

Results

Overall study population

Baseline characteristics

201 individuals were included (full details regarding hospitalisation: n=199; full questionnaire data to assign PCS severity: n=193). The mean age was 44.0 (range 21-71) years and median BMI 25.7 [IQR 23-28]. 71% of individuals were female, 88% were white, 32% were healthcare workers, 19% had been hospitalised with COVID-19. Assessment (symptoms, blood and MRI) was a median 141 (IQR 110-162) days after initial symptoms. Past medical history included smoking (3%), asthma (19%), obesity (20%), hypertension (7%), diabetes (2%) and prior heart disease (5%). The healthy control group had a mean age of 39 years (range 20-70), 40% were female and had a median BMI of 23 [IQR: 21-25] (**Table 1**).

Symptoms

Regardless of hospitalisation, the most frequently reported symptoms were fatigue (98%), shortness of breath (88%), muscle ache (87%), and headache (83%) (**Table 1).** 99% of individuals had four or more and 42% had ten or more symptoms. 70% of individuals reported ≥13 weeks off paid employment. Of the incidental structural findings observed on MRI (n=56), three were cardiac (atrial septal defect, bicuspid aortic valve and right atrial mass), one renal (hydronephrosis), and the rest were benign cysts.

Biochemical investigations

For peer review only

For peer review only - http://bmjopen.bmj.com/site/about/guidelines.xhtml

Haematological investigations including mean corpuscular haemoglobin concentration (MCHC, 24%), and renal, liver and lipid biochemistry, including potassium (38%), alanine transferase (14%), lactate dehydrogenase (17%), triglycerides (11%) and cholesterol (42%) were abnormally high in ≥10% of individuals. Bicarbonate (10%), phosphate (11%), uric acid (11%), and transferrin saturation (19%) were abnormally low in ≥10% of individuals (**Table S2**).

Single and multi- organ impairment

Organ impairment was more common in PCS than healthy controls (**Figure 2**, **supplementary results**). Impairment was present in the heart in 26% (myocarditis 19%; systolic dysfunction 9%), lung in 11% (reduced vital capacity), kidney in 4% (inflammation), liver in 28% (12% inflammation; 21% ectopic fat, 10% hepatomegaly) and pancreas in 40% (15% inflammation, 38% ectopic fat); and spleen in 4% (splenomegaly). (**Table 2, Figure 2).** 70% of individuals had impairment in at least one organ. 29% of individuals had multi-organ impairment, with overlap across multiple organs (**Figure 3**).

Hospitalised versus non-hospitalised

The hospitalised group were older (p=0.001), had higher BMI (p=0.063), were more likely to be non-white (p=0.016), and to report 'inability to walk' (p=0.009) than non-hospitalised individuals. There were no other statistically significant differences between risk factors or symptoms between the groups. Impairment of liver, pancreas (e.g. ectopic fat in the pancreas and liver, hepatomegaly) and ≥2 organs was higher in hospitalised individuals (all p <0.05)(**Table 2, Figure 3)**. In multivariate analyses, adjusting for age, sex and BMI, liver

For peer review only - http://bmjopen.bmj.com/site/about/guidelines.xhtml

volume remained significantly associated with hospitalisation (p=0.001). Hospitalised individuals had high triglycerides (30% vs 7.2%, p=0.002), cholesterol (60 vs 38%, p=0.04) and LDL-cholesterol (57 vs 31%, p=0.01), and low transferrin saturation (38 vs 15%, p=0.01), compared with non-hospitalised individuals. ESR (13%), bicarbonate (12%), uric acid (16%), platelet count (13%) and high-sensitivity CRP (15%) were high in ≥10% of hospitalised individuals (**Table S2**).

For peer review only

Severe versus moderate post COVID syndrome

60% (n=120) had severe PCS, with 52% reporting persistent, moderate problems undertaking usual activities (level 3 or greater in the relevant EQ-5D-5L question; 34% reported Dyspnoea-12 ≥10). Of those with severe PCS, 84% were not hospitalised, and 73% were female. There was no differences in age, BMI or ethnicity between the groups. Individuals with severe PCS were more likely to report shortness of breath (p<.001), headache (p=0.019), chest pain (p=0.001), abdominal pain (p=0.001) and wheezing (p=0.039). 25% of those with 'severe' PCS had myocarditis compared to 12% with moderate PCS (unadjusted: 0.023; adjustment for age, sex and BMI: p=0.04, **Figure S2**). Severe PCS was associated with higher mean cell haemoglobin concentration (28% versus 17%), cholesterol (46.2 versus 32.8), CRP (10% versus 3.8%) and ESR (10% versus 6%), than moderate PCS, but these differences were not statistically significant (**Table S3**). Muscle aches, fever and coughing were common in severe PCS, and headache was common in individuals with pancreas inflammation (**Figure 4**).

For peer review only - http://bmjopen.bmj.com/site/about/guidelines.xhtml

Discussion

We report three findings in the first COVID-19 recovery study to evaluate medium-term, multi-organ impairment. First, in low-risk individuals, there were chronic symptoms and mild impairment in the heart, lung, liver, kidney and pancreas four months post-COVID-19, compared with healthy controls. Second, cardiac impairment was more common in severe PCS. Third, we demonstrate feasibility and potential utility of community-based multi-organ assessment for PCS.

Strengths and limitations

Our study is an ongoing, prospective, longitudinal COVID-19 recovery study with biochemical and imaging characterisation of organ function, starting in April before recognition of “long-COVID”, proper testing availability and prospective COVID-19-related research. By recruiting ambulatory patients with broad inclusion criteria, we focused on a real world population at lower risk of COVID-19 severity and mortality. Our cardiac MRI protocol excluded gadolinium contrast due to concerns regarding COVID-19-related renal complications, relying on native T1 mapping to characterise myocardial inflammation non-invasively (previously validated for acute myocarditis)[29]. For organ impairment, we show association, not causation, and incidental findings are possible in asymptomatic individuals[30], but our findings are strengthened by comparison with healthy, age-matched controls, although not matched for sex or baseline comorbidities. Further studies may explore different controls, e.g. individuals with post-flu symptoms, COVID-19 without symptoms or from general clinics. We will

For peer review only - http://bmjopen.bmj.com/site/about/guidelines.xhtml

investigate duration, trajectory, complications and recovery for specific symptoms and organ impairment in the follow-up phase. Our study population was not ethnically diverse, despite disproportionate COVID-19 impact in non-white individuals. To limit interaction and exposure between trial team and patients, pulse oximetry, spirometry, MRI assessment of brain and muscle function were not included from the outset.

Comparison with other studies

Common symptoms were fatigue, dyspnoea, myalgia, headache and arthralgia, despite low risk of COVID-19 mortality or hospitalisation. COVID-19 impact models have included age, underlying conditions and mortality, but not morbidity, multi-organ impairment and chronic diseases[31,32]. Even in non-hospitalised individuals, up to 10% of those infected have PCS[15, 33], but studies of extra-pulmonary manifestations emphasise acute illness[34]. We describe mild rather than severe organ impairment, but the pandemic's scale and high infection rates in lower risk individuals signal medium- and longer- term COVID-19 impact, which cannot be ignored in healthcare or policy spheres.

Acute myocarditis and cardiogenic shock[35] are documented in hospitalised COVID-19 patients[6]. In American athletes, recent COVID-19 was associated with myocarditis[36]. Although causality cannot be attributed, and post-viral syndromes have included similar findings[21], we show that one quarter of low-risk individuals with PCS have mild systolic dysfunction or myocarditis. The significance of these findings and associations with contemporaneous abnormal echocardiography findings and long-term myocardial fibrosis and impairment are unknown. Cardiac impairment, a risk factor for severe COVID-19, may have a role in PCS. Two further findings deserving investigation are pancreatic abnormalities, given the excess diabetes risk reported in PCS(15), and the preponderance of healthcare workers

For peer review only - http://bmjopen.bmj.com/site/about/guidelines.xhtml

at increased PCS risk (as observed for COVID-19 mortality), possibly due to higher viral burden.

PCS is likely to be a syndrome rather than a single condition. Despite an immunologic basis for individual variations in COVID-19 progression and severity [37], prediction models have high rates of bias, perform poorly[38], and focus on respiratory dysfunction and decisions for ventilation in acutely unwell patients, rather than multi-organ function. Ongoing long-term studies[39] exclude non-hospitalised, low-risk individuals. During a pandemic, we studied subclinical organ impairment in PCS, showing low rates of incidental findings. As specialist PCS services are rolled out[40,41], multi-organ assessment, monitoring and community pathways have potential roles during and beyond COVID-19, but need to be evaluated.

Implications for research, clinical practice and public health

Our findings have three research implications. First, as countries face second waves, COVID-19 impact models should include PCS, whether quality of life, healthcare utilisation, or economic effects. Second, there is urgent need for multi-organ assessment, including blood and imaging, as well as primary and secondary care data linkage, to define PCS. Third, longitudinal studies of clustering of symptoms and organ impairment will inform health services research to plan multidisciplinary care pathways. There are three management implications. First, we signal the need for multi-organ monitoring in at least the medium-term, especially extra-pulmonary sequelae. Care pathways involving MRI (with limited access in many clinical settings) need evaluation versus other modalities to detect organ impairment (e.g. spirometry, NT-pro-BNP, ECG, echocardiography, ultrasound and blood investigations). Second, until effective vaccines and treatments are widely available, "infection suppression" (e.g. social distancing, masks, physical isolation) is the prevention strategy. Third, whether understanding baseline risk or multi-organ complications, PCS requires management across specialities (e.g.

For peer review only

cardiology, gastroenterology) and disciplines (e.g. epidemiology, diagnostics, laboratory science)(**Figure 5**).

Conclusions

Our study suggests PCS has a physiological basis, with measurable patient-reported outcomes and organ impairment. Future research should address longer-term follow-up of organ function beyond symptoms and blood investigations, even in lower risk individuals; prioritisation for imaging, investigation and referral; and optimal care pathways. Health system responses should emphasise infection suppression, and management of pre- and post-COVID-19 risk factors and chronic diseases.

Contributorship statement:

Study design: AD, SK, RB, JA

Patient recruitment: SK, RB, COVERSCAN team

Data collection: MW, LM, COVERSCAN team

Data analysis: AD, COVERSCAN team, AB

Data interpretation: AB, AD, MW, RB

Initial manuscript drafting: AB, AD, RB

Critical review of early and final versions of manuscript: all authors

Specialist input: cardiology (MW, AB); general medicine (RB, MH, DW, MC, GAD); long COVID (MH, MC, DW); imaging (MB, RB); statistics (AD); epidemiology/public health (AB); primary care (SK, MG); healthcare management (JA); patient and public involvement (LH, EA).

COVERSCAN study investigators

For peer review only - http://bmjopen.bmj.com/site/about/guidelines.xhtml

Perspectum: Mary Xu, Faezah Sanaei-Nezhad, Andrew Parks, Andrea Borghetto, Matthew D Robson, Petrus Jacobs, John Michael Brady, Carla Cascone, Soubera Rymell, Jacky Law, Virginia Woolgar, Velko Tonev, Claire Herlihy, Rob Suriano, Tom Waddell, Henrike Puchta, Alessandra Borlotti, Arun Jandor, Freddie Greatrex, Robin Jones, Georgina Pitts, Ashleigh West, Marion Maguire, Anu Chandra, Naomi Jayaratne, Dali Wu, Stella Kin, Mike Linsley, Valentina Carapella, Isobel Gordon, George Ralli, John McGonigle, Darryl McClymont, Boyan Ivanov, James Owler, Diogo Cunha, Tatiana Lim, Carlos Duncker, Madison Wagner, Marc Goldfinger, Adriana Roca, Charlotte Erpicum, Matthew David Kelly, Rexford D Newbould, Catherine J Kelly, Andrea Dennis, Sofia Mouchti, Arina Kazimianec, Helena Thomaides-Briers, Rajarshi Banerjee

University College London: Amitava Banerjee

Great Western Hospitals NHS Foundation Trust: Malgorzata Wamil

University of Oxford: Yi-Chun Wang, Tom Waddell

Mayo Clinic: Sandeep Kapur, and Louise McLaughlin

Funding:

This work was supported by the UK's National Consortium of Intelligent Medical Imaging (Industry Strategy Challenge Fund), Innovate UK (Grant 104688) and the European Union's Horizon 2020 research and innovation programme (agreement No 719445). The research was designed, conducted, analysed, and interpreted by the authors independently of the funding sources.

Competing interests: All authors have completed the ICMJE uniform disclosure form at www.icmje.org/coi_disclosure.pdf. AD, RB and MB are employees of Perspectum. All other authors declare no financial relationships with any organisations that might have an interest

For peer review only

in the submitted work in the previous three years; no other relationships or activities that could appear to have influenced the submitted work.

Ethical approval

The study (https://clinicaltrials.gov/ct2/show/NCT04369807) had ethical approval (20/SC/0185).

Data sharing statement

Deidentified participant data are available from the corresponding authors on request.

Transparency statement

AB affirms that this manuscript is an honest, accurate, and transparent account of the reported study; that no important study aspects have been omitted; and that any discrepancies from the study as planned (and, if relevant, registered) have been explained.

Dissemination to participants and related patient and public communities: The results of this study will be disseminated to relevant patient organisations (e.g. Long COVID SOS), policymakers and health professionals.

Provenance and peer review: Not commissioned; externally peer reviewed.

For peer review only

References

1. World Health Organization. Clinical management of severe acute respiratory infection (SARI) when COVID-19 disease is suspected. Interim guidance 13 March 2020. https://apps.who.int/iris/handle/10665/331446
2. Pavon AG, Meier D, Samim D, Rotzinger DC, Fournier S, Marquis P, et al. First Documentation of Persistent SARS-Cov-2 Infection Presenting With Late Acute Severe Myocarditis. Can J Cardiol. 2020;36(8):1326.e5-1326.e7.
3. Puntmann VO, Carerj ML, Wieters I, Fahim M, Arendt C, Hoffmann J, et al. Outcomes of Cardiovascular Magnetic Resonance Imaging in Patients Recently Recovered from Coronavirus Disease 2019 (COVID-19). JAMA Cardiol. 2020;2019(11):1265–73.
4. Tabary M, Khanmohammadi S. Pathologic features of COVID-19: A concise review. Pathol - Res Pract. 2020;216(9): 153097.
5. Alqahtani SA, Schattenberg JM. Liver injury in COVID-19: The current evidence. United Eur Gastroenterol J. 2020;8(5):509–19.
6. Somasundaram NP, Ranathunga I, Ratnasamy V, Wijewickrama PSA, Dissanayake HA, Yogendranathan N, et al. The impact of SARS-Cov-2 virus infection on the endocrine system. J Endocr Soc. 2020;4(8):1–22.
7. Farouk SS, Fiaccadori E, Cravedi P, Campbell KN. COVID-19 and the kidney: what we think we know so far and what we don't. J Nephrol. 2020. Jul 20 : 1–6.

For peer review only - http://bmjopen.bmj.com/site/about/guidelines.xhtml

For peer review only

8. Lai A, Pasea L, Banerjee A, Denaxas S, Katsoulis M, Chang W, et al. Estimating excess mortality in people with cancer and multimorbidity in the COVID-19 emergency. BMJ Open. 2020. Nov 17;10(11):e043828.
9. Banerjee A, Pasea L, Harris S, Gonzalez-Izquierdo A, Torralbo A, Shallcross L, et al. Estimating excess 1-year mortality associated with the COVID-19 pandemic according to underlying conditions and age: a population-based cohort study. Lancet. 2020;395(10238):1715–25.
10. Banerjee A, Chen S, Pasea L, Lai A, Katsoulis M, Denaxas S, et al. Excess deaths in people with cardiovascular diseases during the COVID-19 pandemic. Eur J Prev Cardiol. 2020. In press.
11. Horton R. Offline: COVID-19 is not a pandemic. Lancet. 2020;396(10255):874.
12. Shovlin CL, Vizcaychipi MP. Implications for COVID-19 triage from the ICNARC report of 2204 COVID-19 cases managed in UK adult intensive care units. Emerg Med J. 2020;37(6):332–3.
13. Docherty AB, Harrison EM, Green CA, Hardwick HE, Pius R, Norman L, et al. Features of 20 133 UK patients in hospital with covid-19 using the ISARIC WHO Clinical Characterisation Protocol: Prospective observational cohort study. BMJ. 2020;369:1–12.
14. Williamson E, Walker A, Bhaskaran K, Bacon S, Curtis H, Mehrkar A, et al. Factors associated with COVID-19-related death using OpenSAFELY. Nature. 2020; 584(7821):430-436.
15. Office for National Statistics. The prevalence of long COVID symptoms and COVID-19 complications. 16 December 2020. https://www.ons.gov.uk/news/statementsandletters/theprevalenceoflongcovidsymptomsandcovid19complications
16. Del Rio C, Collins L, Malani P. Long-term Health Consequences of COVID-19 Carlos. JAMA. 2020;324(17).
17. Carfi A, Bernabei R, Landi F. Persistent Symptoms in Patients After Acute COVID-19. JAMA. 2020;324(6):604–5.
18. Nabavi N. Long covid: How to define it and how to manage it. BMJ. 2020;370:m3489.
19. Greenhalgh T, Knight M, A'Court C, Buxton M, Husain L. Management of post-acute covid-19 in primary care. BMJ. 2020;370.
20. National Institute for Health Research: New risk prediction model could help improve guidance for people shielding from COVID-19. June 2020. https://www.nihr.ac.uk/news/new-risk-prediction-model-could-help-improve-guidance-for-people-shielding-from-covid-19/25096

For peer review only

21. Hill AD, Fowler RA, Pinto R, Herridge MS, Cuthbertson BH, Scales DC. Long-term outcomes and healthcare utilization following critical illness - a population-based study. Crit Care. 2016;20(1):1–10.

22. Perrin R. Into the looking glass: Post-viral syndrome post COVID-19. Med Hypotheses. 2020;144.

23. George PM, Barratt SL, Condliffe R, Desai SR, Devaraj A, Forrest I, et al. Respiratory follow-up of patients with COVID-19 pneumonia. Thorax. 2020;75(11):1009–16.

24. Jiang DH, McCoy RG. Planning for the Post-COVID Syndrome: How Payers Can Mitigate Long-Term Complications of the Pandemic. J Gen Intern Med. 2020;35(10):3036–9.

25. Janssen MF, Pickard AS, Golicki D, Gudex C, Niewada M, Scalone L, et al. Measurement properties of the EQ-5D-5L compared to the EQ-5D-3L across eight patient groups: A multi-country study. Qual Life Res. 2013;22(7):1717–27.

26. Yorke J, Moosavi SH, Shuldham C, Jones PW. Quantification of dyspnoea using descriptors: development and initial testing of the Dyspnoea-12. Thorax. 2010;65(1):21–6.

27. Hobbins A, Barry L, Kelleher D, O'Neill C. The health of the residents of Ireland: Population norms for Ireland based on the EQ-5D-5L descriptive system – a cross sectional study. HRB Open Res. 2018;1:22.

28. Raman B, Philip Cassar M, Tunnicliffe EM, Filippini N, Griffanti L, Alfaro-Almagro F, et al. Medium-term effects of SARS-CoV-2 infection on multiple vital organs, exercise capacity, cognition, quality of life and mental health, post-hospital discharge. medRxiv. 2020. 18 October 2020. https://www.medrxiv.org/content/10.1101/2020.10.15.20205054v1.

29. Ferreira VM, Piechnik SK, Armellina ED, Karamitsos TD, Francis JM, Ntusi N, et al. Native T1-mapping detects the location , extent and patterns of acute myocarditis without the need for gadolinium contrast agents. 2014;16(1):1–11.

30. Gibson LM, Paul L, Chappell FM, Macleod M, Whiteley WN, Salman RAS, et al. Potentially serious incidental findings on brain and body magnetic resonance imaging of apparently asymptomatic adults: Systematic review and meta-analysis. BMJ. 2018;363: k4577.

31. Gupta A, Madhavan M V., Sehgal K, Nair N, Mahajan S, Sehrawat TS, et al. Extrapulmonary manifestations of COVID-19. Nat Med. 2020;26(7):1017–32.

32. Palmer K, Monaco A, Kivipelto M, Onder G, Maggi S, Michel JP, et al. The potential long-term impact of the COVID-19 outbreak on patients with non-communicable diseases in Europe: consequences for healthy ageing. Aging Clin Exp Res [Internet].

For peer review only

2020;32(7):1189–94.

33. Menni C, Valdes AM, Freidin MB, Sudre CH, Nguyen LH, Drew DA, et al. Real-time tracking of self-reported symptoms to predict potential COVID-19. Nat Med. 2020;26(7):1037–40.

34. Mandal S, Barnett J, Brill S, Brown J, Denneny E, Hare S, et al. “Long-COVID”: a cross-sectional study of persisting symptoms, biomarker and imaging abnormalities following hospitalisation for COVID-19. Thorax. 2020; Nov 10;thoraxjnl-2020-215818.

35. Chau VQ, Giustino G, Mahmood K, Oliveros E, Neibart E, Oloomi M, et al. Cardiogenic shock and hyperinflammatory syndrome in young males with COVID-19. Circ Hear Fail. 2020;556–9.

36. Rajpal S, Tong MS, Borchers J, Zareba KM, Obarski TP, Simonetti OP, et al. Cardiovascular Magnetic Resonance Findings in Competitive Athletes Recovering from COVID-19 Infection. JAMA Cardiol. 2020;5–7.

37. Mathew D, Giles JR, Baxter AE, Oldridge DA, Greenplate AR, Wu JE, et al. Deep immune profiling of COVID-19 patients reveals distinct immunotypes with therapeutic implications. Science. 2020; 369(6508):eabc8511.

38. Wynants L, Van Calster B, Collins GS, Riley RD, Heinze G, Schuit E, et al. Prediction models for diagnosis and prognosis of covid-19: Systematic review and critical appraisal. BMJ. 2020;369 :m1328.

39. PHOSP-COVID: Post-HOSPitalisation COVID-19 study. https://www.phosp.org/

40. NHS to offer ‘long covid’ sufferers help at specialist centres. https://www.england.nhs.uk/2020/10/nhs-to-offer-long-covid-help/

41. National Institute for Healh and Care Excellence. COVID-19 rapid guideline: managing the long-term effects of COVID-19. 18 December 2020. https://www.nice.org.uk/guidance/ng188

For peer review only

Figures and Table Titles and Legends

Table 1: Baseline demographics and symptoms in 201 low-risk individuals with post COVID syndrome.

[*] Data are presented as count (%). Comparisons between managed at home vs hospitalised, and between moderate vs post-COVID syndrome were conducted using Fisher's exact test.

Table 2: Evidence of organ impairment in 201 low-risk individuals with post COVID syndrome.

[*] Data are presented as count (%). Comparisons between managed at home vs hospitalised, and between moderate vs post-COVID syndrome were conducted using Fisher's exact test.

Figure 1: Flow from recruitment to enrolment of 201 patients with post COVID syndrome.

For peer review only - http://bmjopen.bmj.com/site/about/guidelines.xhtml

Figure 2. Organ impairment in low-risk individuals with post COVID syndrome (n=201) compared to healthy controls (n=36).

*Significance: · p=0.05; *p<0.05; **p<0.01; *** p<.001.*

Figure 3: Multi-organ impairment in low-risk individuals with post COVID syndrome by gender and hospitalisation.

Figure 4: Reported symptoms and organ impairment in individuals with severe post COVID syndrome.

Darker red indicates higher percentage of reported symptoms per impaired organ.

Figure 5: Natural history of post COVID syndrome, the COVERSCAN study in low-risk individuals (n=201) and policy recommendations.

For peer review only

Table 3: Baseline demographics and symptoms in 201 low-risk individuals with post COVID syndrome.

	All Patients (n=201)	Healthy Controls (N=36)	P	Not hospitalised (n=163)	Hospitalised (n=37)	P	Moderate PCS (n=77)	Severe PCS (n=116)	p
**Age** (yrs, mean; sd)	44 (11.0)	39 (12.4)	0.013	43 (10.9)	50 (10.0)	0.001	45 (12.2)	44 (10.0)	0.419
**Female** (No, %)	142 (70.6)	14 (38.9)	0.032	118 (72.4)	23 (62.2)	0.302	51 (66.2)	85 (73.3)	0.374
**BMI** (kg.m^{-2}); median(IQR)	25.7(22.7-28.1)	23.2 (21.4-23.1)	<0.001	25.3 (22.7-27.7)	27.2 (23.1-31.0)	0.063	25.8 (22.7-27.9)	25.4 (22.5-28.2)	0.639
Ethnicity									
White	176 (87.6)	33 (91.7)	0.904	148 (90.8)	28 (75.7)	0.016	67 (87.0)	106 (91.4)	0.178
Mixed	3 (1.5)	0 (0)		3 (1.8)	0 (0)		1 (1.3)	2 (1.7)	
South Asian	7 (3.5)	3 (8.3)		4 (2.5)	3 (8.1)		5 (6.5)	0 (0)	
Black	4 (2.0)	0 (0)		1 (0.6)	2 (5.4)		2 (2.6)	2 (1.7)	
**Comorbidities and risks**									
Smoking									
Never	133 (66.2)	20 (60.6)		108 (66.3)	24 (64.9)		55 (71.4)	72 (61.7)	
Current	6 (3.0)	8 (24.2)	<0.001	6 (3.7)	0 (0)	0.641	3 (3.9)	3 (2.6)	0.244
Ex-smoker	62 (30.8)	5 (15.2)		49 (30.1)	13 (35.1)		19 (24.7)	41 (35.3)	
**Health care worker**	64 (31.8)	4 (12.1)	0.009	50 (30.7)	13 (35.1)	0.695	33 (42.9)	28 (24.1)	0.007
**Asthma**	37 (18.4)	0 (0)	0.002	34 (20.9)	3 (8.1)	0.099	13 (16.9)	22 (19.0)	0.849
**BMI**									
≥25 kg/m^2	113 (56.5)	7 (20)		87 (53.7)	25 (67.6)	0.144	46 (60.5)	62 (53.4)	0.374
≥30 kg/m^2	40 (20.0)	0 (0)		28 (17.3)	12 (32.4)	0.066	16 (21.1)	24 (20.7)	1.000
**Hypertension**	13 (6.5)	0 (0)	0.001	11 (6.7)	2 (5.4)	1.000	6 (7.8)	7 (6.0)	0.771
**Diabetes**	4 (2.0)	0 (0)	0.104	4 (2.5)	0 (0.0)	1.000	4 (5.2)	0 (0.0)	0.024
**Previous heart disease**	9 (4.5)	0 (0)	0.001	8 (4.9)	1 (2.7)	1.000	3 (3.9)	5 (4.3)	1.000
**Symptoms**									

For peer review only

Fatigue	196 (98.0)			159 (97.5)	37 (100.0)	1.000	73 (96.1)	115 (99.1)	0.302
Shortness of breath	176 (88.0)			141 (86.5)	35 (94.6)	0.262	58 (76.3)	112 (96.6)	<0.0001
Muscle ache	173 (86.5)			142 (87.1)	31 (83.8)	0.597	66 (86.8)	101 (87.1)	1.000
Headache	165 (82.5)			138 (84.7)	27 (73.0)	0.098	56 (73.7)	102 (87.9)	0.019
Joint pain	156 (78.0)			127 (77.9)	29 (78.4)	1.000	56 (73.7)	94 (81.0)	0.284
Chest pain	152 (76.0)			128 (78.5)	24 (64.9)	0.090	47 (61.8)	98 (84.5)	0.001
Cough	146 (73.0)			117 (71.8)	29 (78.4)	0.539	55 (72.4)	84 (72.4)	1.000
Fever	144 (72.0)			113 (69.3)	31 (83.8)	0.104	51 (67.1)	86 (74.1)	0.329
Sore throat	143 (71.5)			120 (73.6)	23 (62.2)	0.165	50 (65.8)	86 (74.1)	0.256
Diarrhoea	118 (59.0)			91 (55.8)	27 (73.0)	0.065	40 (52.6)	76 (65.5)	0.097
Abnormal pain	108 (54.0)			91 (55.8)	17 (45.9)	0.361	30 (39.5)	75 (64.7)	0.001
Wheezing	98 (49.0)			75 (46.0)	23 (62.2)	0.101	30 (39.5)	64 (55.2)	0.039
Inability to walk	80 (40.0)			58 (35.6)	22 (59.5)	0.009	24 (31.6)	50 (43.1)	0.130
Runny nose	68 (34.0)			55 (33.7)	13 (35.1)	0.85	24 (31.6)	41 (35.3)	0.642
Time interval									
Initial symptoms-to-assessment (days): median (IQR)	141 (110, 162)			141 (112-163)	138 (97-150)	0.106	121 (89-158)	145 (121-163)	0.001
COVID-19 positive-to-assessment (days): median, (IQR)	71 (41, 114			68 (35-112)	105 (59-126)	0.012	60 (43,98)	78 (34-119)	0.305

Table 4: Evidence of organ impairment in 201 low-risk individuals with post COVID syndrome.

Measurement	All Patients (n=201)	Healthy Controls (n=36)	P	Not hospitalised (n=163)	Hospitalised (n=37)	P	Moderate PCS (n=77)	Severe PCS (n=116)	P
HEART									
Left ventricular ejection fraction (%)									
• Normal (>51%)	190 (95.0)	35 (97.2)	0.699	155 (95.7)	33 (89.1)	0.124	72 (93.5)	111 (95.7)	0.353
• Impaired (≤51%)	11 (5.0)	1 (2.8)		7 (4.3)	4 (10.1)		5 (6.4)	5 (4.3)	
Left ventricular end diastolic volume (ml)									
• >264ml in M; >206ml in W	8 (4.0)	1 (2.8)	1.00	4 (2.5)	4 (10.8)	0.040	4 (5.2)	4 (3.4)	0.715
Evidence of myocarditis									
• ≥ 3 segments with high T1 (≥1229ms at 3T; ≥1015ms at 1.5T)	39 (19.4)	2 (5.6)	0.053	30 (18.4)	8 (21.6)	0.647	9 (11.7)	29 (25.0)	0.027

For peer review only - http://bmjopen.bmj.com/site/about/guidelines.xhtml

LUNGS									
Deep Breathing Fractional area change	(n=17 missing)			(n=13 missing)	(n=3 missing)		(n=8 missing)	(n=7 missing)	
• < 31%	21 (11.4)	1 (2.8)	0.138	17 (11.3)	4 (11.8)	1	7 (10.1)	13 (11.9)	0.811
KIDNEYS									
Kidney cortex T1	(n=3 missing)			(n=3 missing)			(n=2 missing)		
• Normal (<1652 ms at 3T; <1227ms at 1.5T)	191 (96.5)	36 (100.0)	0.599	155 (96.9)	35 (94.6)	0.618	74 (98.7)	112 (96.6)	0.65
• Impaired (≥1652 ms at 3T; ≥1227ms at 1.5T)	7 (3.5)	0 (0.0)		5 (3.1)	2 (5.4)		1 (1.3)	4 (3.4)	
PANCREAS									
Pancreatic inflammation (T1 in ms)	(n=11 missing)	(n=13 missing)		(n=7 missing)	(n=4 missing)		(n=4 missing)	(n=6 missing)	
• Normal <803ms	162 (85.3)	23 (100.0)	0.049	139 (89.1)	22 (66.7)	0.002	60 (82.2)	95 (86.4)	0.530
• Impaired ≥803ms	28 (14.7)	0 (0)		17 (10.9)	11 (33.3)		13 (17.8)	15 (13.6)	
Pancreatic fat		(n=4 missing)							
• Normal <4.6%	122 (62.2)	30 (93.8)	<0.001	107 (66.9)	14 (40.0)	0.004	44 (57.9)	72 (63.7)	0.449
• Impaired ≥4.6%	74 (37.8)	2 (6.2)		53 (33.1)	21 (60.0)		32 (42.1)	41 (36.3)	
LIVER									
Liver Inflammation (cT1 in ms)	(n=1 missing)			(n=1 missing)			(n=1 missing)		
• Normal <784ms	177 (88.5)	36 (100)	0.030	148 (91.4)	28 (75.7)	0.018	69 (90.8)	101 (87.1)	0.494
• Impaired ≥784ms	23 (11.5)	0 (0)		14 (8.6)	9 (24.3)		7 (9.2)	15 (12.9)	
Liver fat									
• Normal <4.8%	159 (79.1)	34 (94.4)	0.034	134 (82.2)	24 (64.9)	0.026	61 (79.2)	91 (78.4)	1
• Impaired ≥4.8%	42 (20.9)	2 (5.4)		29 (17.8)	13 (35.1)		16 (20.8)	25 (21.6)	
Liver volume		(n=1 missing)							
• Normal <1935ml	180 (89.6)	34 (97.1)	0.214	154 (94.5)	25 (67.6)	<0.0001	68 (88.3)	104 (89.7)	0.816
• Impaired ≥1935ml	21 (10.4)	1 (2.9)		9 (5.5)	12 (32.4)		9 (11.7)	12 (10.3)	
SPLEEN									
Splenic volume (ml)		(n=1 missing)							
• Normal <350ml	194 (96.5)	32 (91.4)	0.172	160 (98.2)	33 (89.2)	0.023	74 (96.1)	112 (96.6)	1
• Impaired ≥350ml	7 (3.5)	3 (8.6)		3 (1.8)	4 (10.8)		3 (3.9)	4 (3.4)	

For peer review only - http://bmjopen.bmj.com/site/about/guidelines.xhtml

Data are presented as count (%). Comparisons between managed at home vs hospitalised, and between moderate vs post-COVID syndrome were conducted using Fisher's exact test.

For peer review only

For peer review only - http://bmjopen.bmj.com/site/about/guidelines.xhtml

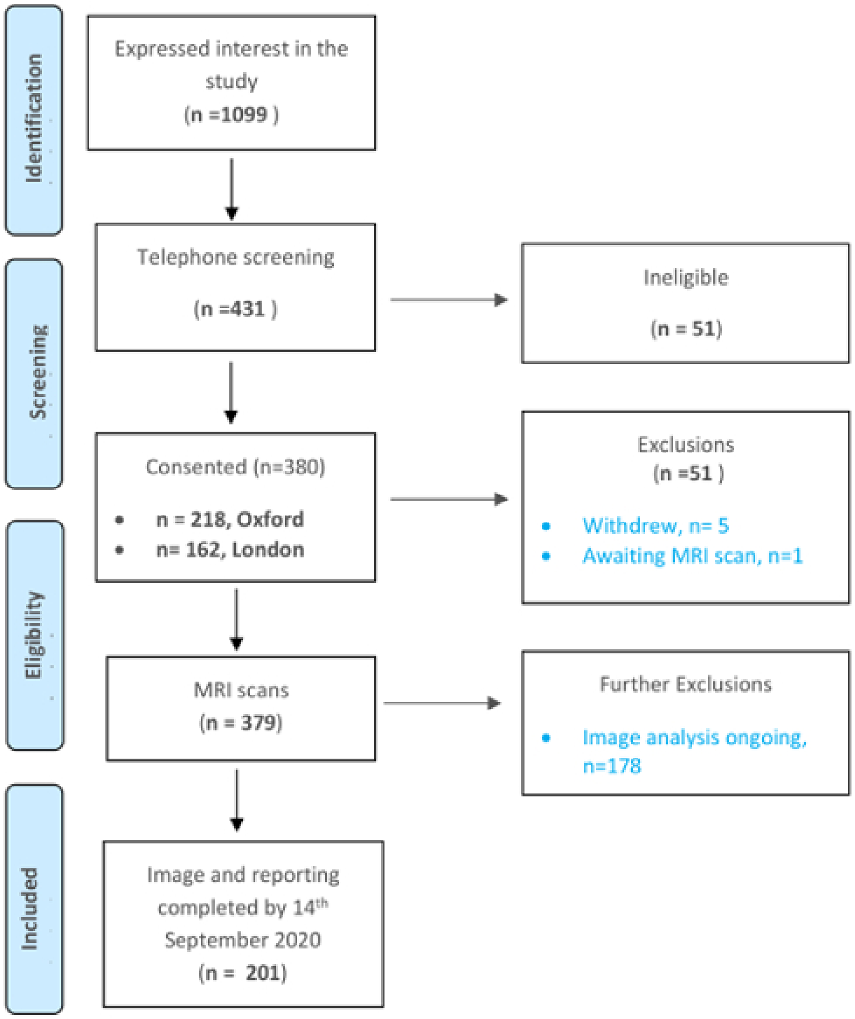

Figure 1. Flow from recruitment to enrolment of 201 patients with post COVID syndrome.

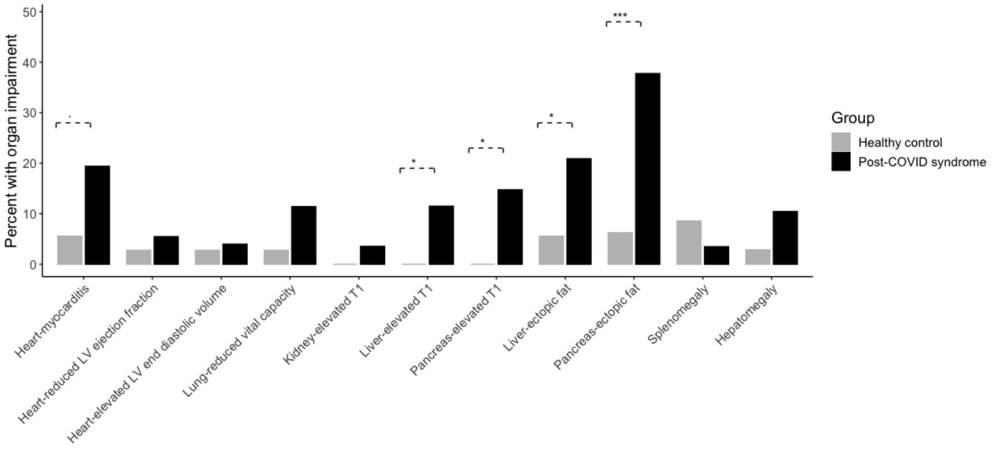

Figure 2. Organ impairment in low-risk individuals with post COVID syndrome (n=201) compared to healthy controls (n=36).

Significance: . p=0.05; *p<0.05; **p<0.01; *** p<.001.

418x206mm (72 x 72 DPI)

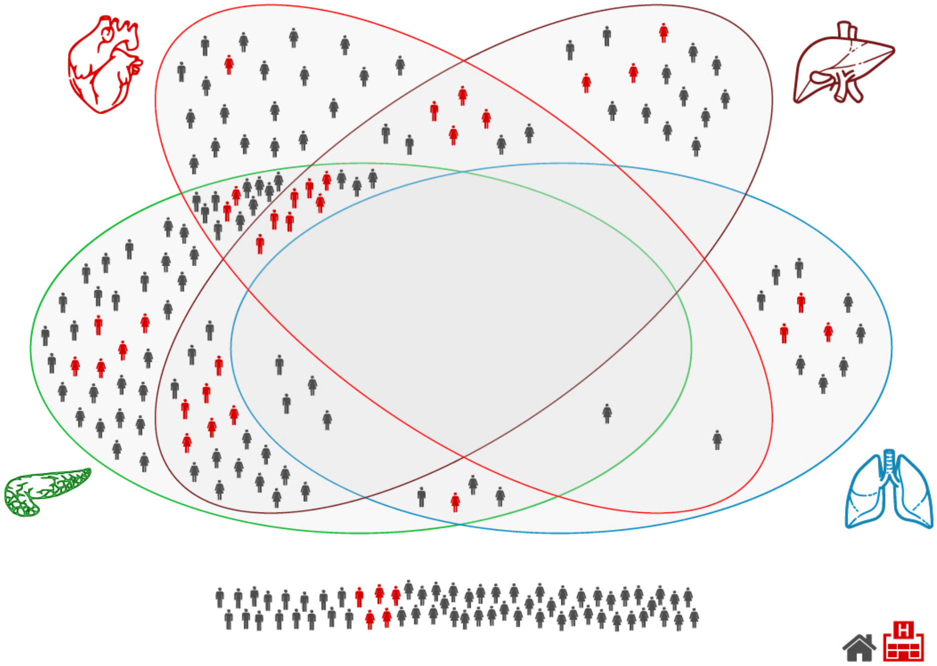

Figure 3. Multi-organ impairment in low-risk individuals with post COVID syndrome by gender and hospitalisation.

198x141mm (144 x 144 DPI)

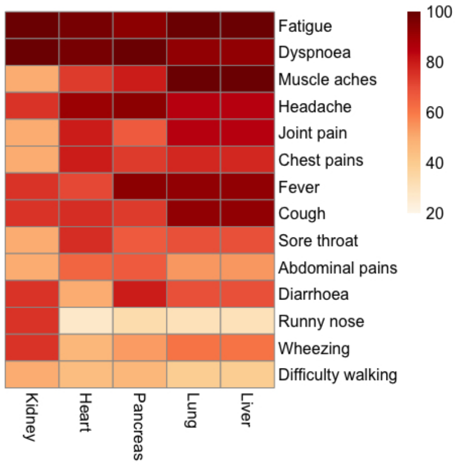

Figure 4: Reported symptoms and organ impairment in individuals with severe post COVID syndrome.

Darker red indicates higher percentage of reported symptoms per impaired organ.

349x206mm (72 x 72 DPI)

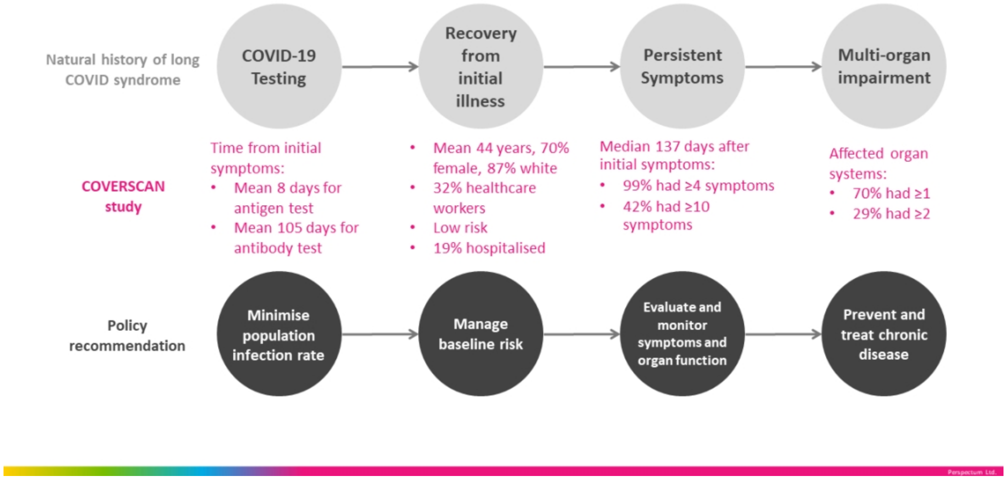

Figure 5: Natural history of post COVID syndrome, the COVERSCAN study in low-risk individuals (n=201) and policy recommendations.

338x190mm (96 x 96 DPI)

Web Supplementary Materials

Supplementary methods 2

Supplementary references 4

Supplementary results 6

Figure S1: Comparison of patients to control quantitative image derived measures in a subset of those scanned at 1.5T 6

Table S1: Reference ranges to define organ impairment 7

Figure S2: Organ impairment in severe versus moderate post COVID syndrome (n=201) 7

Table S2: Blood investigations in 201 low-risk individuals with post-COVID syndrome, sub-divided by hospitalisation or managed at home 8

Table S3: Blood investigations in 201 low-risk individuals, sub-divided by those with severe of moderate post-COVID syndrome 13

For peer review only - http://bmjopen.bmj.com/site/about/guidelines.xhtml

Supplementary methods

Blood investigations

Blood investigations included: full blood count, serum biochemistry (sodium, chloride, bicarbonate, urea, creatinine, bilirubin, alkaline phosphatase, aspartate transferase, alanine transferase, lactate dehydrogenase, creatinine kinase, gamma-glutamyl transpeptidase, total protein, albumin, globulin, calcium, magnesium, phosphate, uric acid, fasting triglycerides, cholesterol (total, HDL, LDL), iron, iron-binding capacity (unsaturated and total) and inflammatory markers (erythrocyte sedimentation rate, ESR; high sensitivity-C-Reactive Protein, CRP) (TDL laboratories, London).

Imaging

All the imaging methods can be deployed on standard clinical MRI scanners and are generally expedited approaches of methods previously demonstrated in the scientific literature that unless stated each utilise a short (<14seconds) breath-hold.

Cardiac imaging involved complete coverage of the heart with a short-axis stack (to the valve plane) of cine images acquired using cardiac gating, this acquisition mirrors that in UK Biobank and is a standardized approach(S1). Three short-axis cardiac T1 maps are acquired using the MOLLI-T1 approach at the basal, mid and apical levels of the left ventricle.

Liver and pancreas imaging used the LiverMultiScan acquisition protocol (Perspectum, Oxford, UK), which involves 3 single 2D axial slice breath-held acquisitions that separately are sensitive to the fat content (proton density fat fraction, or PDFF), to T2* (which is representative of liver iron content) and a MOLLI-T1 measurement (providing a measurement of tissue water), additionally a volumetric scan was used that covers the entire liver(S2).

Two dynamic cine MR acquisitions of the lung were acquired in the coronal plane with a 306.91 ms temporal resolution: one 40 s acquisition with the patient instructed to breathe normally and a second 30 s acquisition with the patient instructed to breathe deeply.

Kidney imaging used a single coronal view that was able to image both kidneys, imaging contrasts were MOLLI-T1, T2* (for blood oxygen level assessment), and diffusion imaging that was acquired during free-breathing in 2minutes.

Image Analysis

Cardiac MRI Analysis: Experienced cardiac MRI analysts used CVI42 (Cardiovascular Imaging Inc, Canada) to manually trace the end-diastolic and end-systolic phases in each of the short-axis views, following the standard UK BioBank evaluation approach as previously described(S3). This analysis yielded: For both the left and the right ventricle; End diastolic volume, End systolic volume, Stroke volume and Ejection Fraction. Additionally left ventricular muscle mass and wall thickness are determined from the function data. Cardiac T1 was determined for each of the 16 cardiac segments (of the AHA 17 segment model)(S4).

Liver Images were analysed by data analysts experienced at using the LiverMultiScan (Perspectum, Oxford, UK) software. This yielded global metrics in each liver of PDFF (proton density fat fraction), T2*, and cT1 (cT1 is a measurement of T1 that has been corrected for the confounding effects of iron and standardised to 3 Tesla; it is elevated with disease).

Pancreas images were analysed in a similar manner to the above except the software used was not FDA-cleared and iron correction was not performed. The output T1 was standardized to 3 Tesla.

For peer review only - http://bmjopen.bmj.com/site/about/guidelines.xhtml

Lung cine imaging allowed the measurement of the area of the left and right lungs through the breathing cycle in the coronal plane, which used automated methods that were reviewed by image analysts. The periodicity of the area fluctuations was used to determine the respiratory rate. All analysis was performed in-house using MATLAB based tools. The method was validated by measuring the correlation between the change in area and the forced vital capacity, the latter being measured using spirometry.

Patient respiration was assessed by imaging a single 2D coronal slice of the lungs over 30 seconds using a dynamic cine MRI acquisition, during which the patient instructed to breathe deeply.

Kidney images were assessed using in-house tools to fit the parametric maps and allow trained analysts to make measurements. The T2* maps were analysed by the Twelve Layer Concentric Object (TLCO) approach that generates a gradient of relaxation values, in the other evaluations the cortex and medulla were manually segmented using the MOLLI-T1 map or the b=0 (in the case of diffusion) to guide the boundary.

In all cases the volumetric assessments utilised an initial in-house developed machine-learning driven segmentation, and then a manual step that may be used to fine tune boundaries. This approach was also used in the body composition analysis, which for reasons of speed was performed only in a single slice (an axial view that passes through L3 of the spine) in this work.

For peer review only

For peer review only - http://bmjopen.bmj.com/site/about/guidelines.xhtml

Supplementary references

S1. Petersen SE, Matthews PM, Francis JM, Robson MD, Zemrak F, Boubertakh R, Young AA, Hudson S, Weale P, Garratt S, Collins R, Piechnik S, Neubauer S. UK Biobank's cardiovascular magnetic resonance protocol. J Cardiovasc Magn Reson. 2016 Feb 1;18:8.

S2. Banerjee R, Pavlides M, Tunnicliffe EM, Piechnik SK, Sarania N, Philips R, Collier JD, Booth JC, Schneider JE, Wang LM, Delaney DW, Fleming KA, Robson MD, Barnes E, Neubauer S. Multiparametric magnetic resonance for the non-invasive diagnosis of liver disease. J Hepatol. 2014 Jan;60(1):69-77. doi: 10.1016/j.jhep.2013.09.002.

S3. Petersen SE, Aung N, Sanghvi MM, Zemrak F, Fung K, Paiva JM, Francis JM, Khanji MY, Lukaschuk E, Lee AM, Carapella V, Kim YJ, Leeson P, Piechnik SK, Neubauer S. Reference ranges for cardiac structure and function using cardiovascular magnetic resonance (CMR) in Caucasians from the UK Biobank population cohort. J Cardiovasc Magn Reson. 2017 Feb 3;19(1):18.

S4. Cerqueira MD, Weissman NJ, Dilsizian V, Jacobs AK, Kaul S, Laskey WK, Pennell DJ, Rumberger JA, Ryan T, Verani MS; American Heart Association Writing Group on Myocardial Segmentation and Registration for Cardiac Imaging. Standardized myocardial segmentation and nomenclature for tomographic imaging of the heart. A statement for healthcare professionals from the Cardiac Imaging Committee of the Council on Clinical Cardiology of the American Heart Association. Circulation. 2002 Jan 29;105(4):539-42.

S5. Kawel-Boehm N, Maceira A, Valsangiacomo-Buechel ER, Vogel-Claussen J, Turkbey EB, Williams R, Plein S, Tee M, Eng J, Bluemke DA. Normal values for cardiovascular magnetic resonance in adults and children. J Cardiovasc Magn Reson. 2015 Apr 18;17(1):29.).

S6. Ponikowski P, Voors AA, Anker SD, Bueno H, Cleland JGF, Coats AJS, Falk V, González-Juanatey JR, Harjola VP, Jankowska EA, Jessup M, Linde C, Nihoyannopoulos P, Parissis JT, Pieske B, Riley JP, Rosano GMC, Ruilope LM, Ruschitzka F, Rutten FH, van der Meer P; ESC Scientific Document Group. 2016 ESC Guidelines for the diagnosis and treatment of acute and chronic heart failure: The Task Force for the diagnosis and treatment of acute and chronic heart failure of the European Society of Cardiology (ESC)Developed with the special contribution of the Heart Failure Association (HFA) of the ESC. Eur Heart J. 2016 Jul 14;37(27):2129-2200.

S7. Tsao CW, Lyass A, Larson MG, Cheng S, Lam CS, Aragam JR, Benjamin EJ, Vasan RS. Prognosis of adults with borderline left ventricular ejection fraction. JACC Heart Fail. 2016 Jun;4(6):502-10.

S8. Chalasani, Naga, et al. The diagnosis and management of nonalcoholic fatty liver disease: practice guidance from the American Association for the Study of Liver Diseases. Hepatology 67.1 (2018): 328-357

S9. Mojtahed A, Kelly C, Herlihy A, et al. Reference range of liver corrected T1 values in a population at low risk for fatty liver disease-a UK Biobank sub-study, with an appendix of interesting cases. Abdomimal Radiol 2019; 44: 72–84.

S10. Jayaswal AN, Levick C, Selvaraj EA, et al. Prognostic value of multiparametric MRI, transient elastography and blood-based fibrosis markers in patients with chronic liver disease. Liver Int 2020; in press. DOI:doi:10.1111/liv.14625.

S11. Jayaswal ANA, Levick C, Selvaraj EA, Dennis A, Booth JC, Collier J, Cobbold J, Tunnicliffe EM, Kelly M, Barnes E, Neubauer S, Banerjee R, Pavlides M. Prognostic value of multiparametric magnetic

For peer review only - http://bmjopen.bmj.com/site/about/guidelines.xhtml

resonance imaging, transient elastography and blood-based fibrosis markers in patients with chronic liver disease. Liver Int. 2020 Jul 30. doi: 10.1111/liv.14625

S12. Chouhan MD, Firmin L, Read S, Amin Z, Taylor SA. Quantitative pancreatic MRI: a pathology-based review. Br J Radiol. 2019 Jul;92(1099):20180941.

S13. Harrington KA, Shukla-Dave A, Paudyal R, Do RKG. MRI of the Pancreas. J Magn Reson Imaging. 2020 Apr 17. doi: 10.1002/jmri.27148.

S14. Gillis KA, McComb C, Patel RK, et al. Non-contrast renal magnetic resonance imaging to assess perfusion and corticomedullary differentiation in health and chronic kidney disease. Nephron 2016; 133: 183–92.

S15. Peperhove M, Vo Chieu VD, Jang M-S, et al. Assessment of acute kidney injury with T1 mapping MRI following solid organ transplantation. Eur Radiol 2018; 28: 44–50.

S16. Chow KU, Luxembourg B, Seifried E, Bonig H. Spleen size is significantly influenced by body height and sex: establishment of normal values for spleen size at us with a cohort of 1200 healthy individuals. Radiology 2015; 279: 306–13.

For peer review only

Supplementary results

Sub-group analysis

Data from healthy participants (n=36) scanned on the 1.5T Siemens MRI scanner were compared to the sub-group of patients (N=121) scanned on the same MRI machine. Median global cardiac T1 was elevated in the patient group (979 ms versus 962ms, P=0.001). Lung fractional area difference, a measure of relaxed vital capacity, was significantly lower in the patient group (41% versus 48%, P<.001). Kidney inflammation (1148 vs 1084 ms, p <0.001) was significantly elevated in the patients as were markers of organ fat (liver 2.6% versus 2.1%, p=0.008; pancreas: 4.3% versus 2.5%, p<0.001) (**Figure S1**).

Figure S1: Box plots showing median and interquartile ranges for the healthy control group and the patient group for those scanned at 1.5T. Comparisons between groups were performed using two-sided Kolmogorov-Smirnov (KS) tests. Significance stars are * P<.05; ** P<.01, ***P<.001.

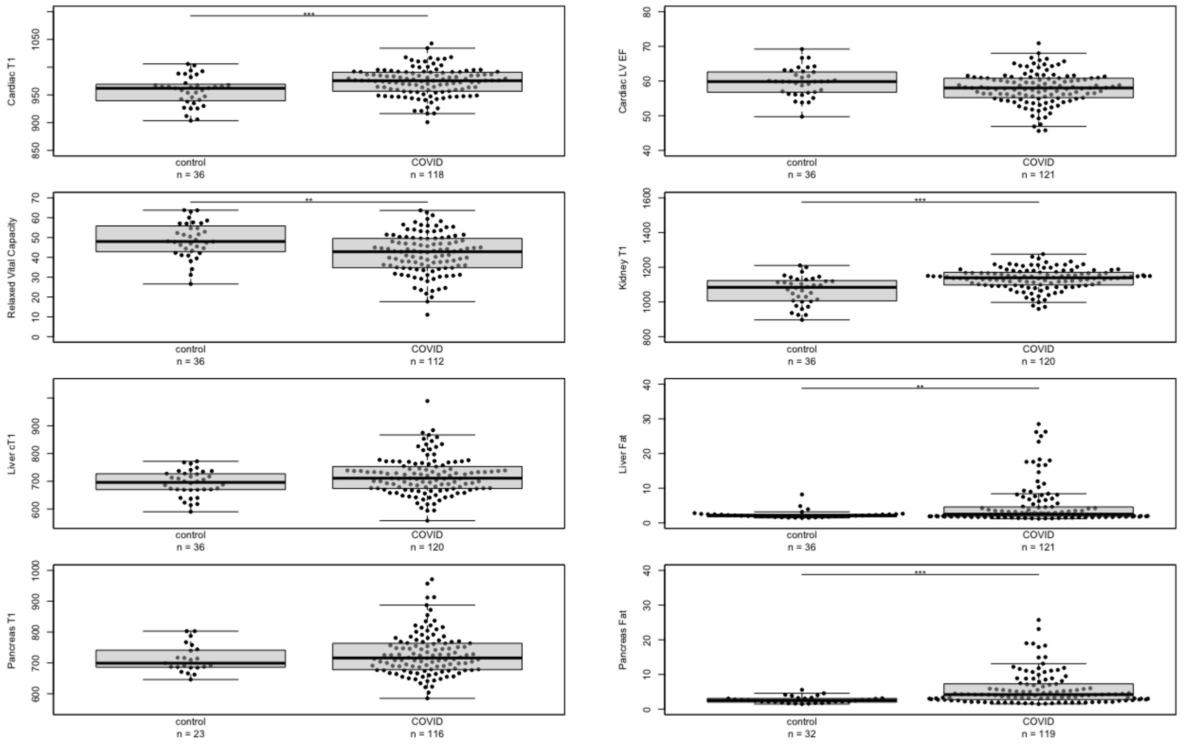

For peer review only - http://bmjopen.bmj.com/site/about/guidelines.xhtml

Figure S2: Organ impairment in severe versus moderate post COVID syndrome (n=201)

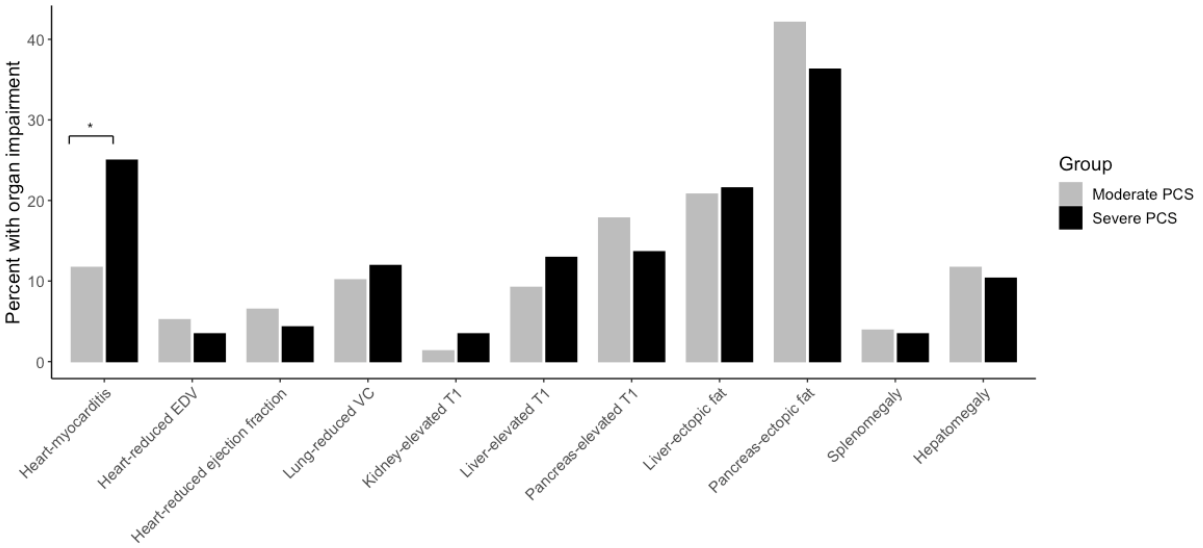

Table S1: Reference ranges for organ impairment, defined as a value that was greater than the mean plus 2 standard deviations of that from the control group for most; mean minus 2 standard deviations for left ventricular ejection fraction and lung fractional area difference for the 1.5T scans. For the 3T scans, this was the value as reported by Raman et al (2020).

	1.5T Reference range	3T reference range
Left ventricular ejection fraction (LVEF) (S4-S7)	≤ 51.5%	----
Increased end-diastolic volume (S4-S7)	≥ 264ml in men<br>≥ 206ml in women	----
Myocarditis (S4-S7)	≥ 1015 ms	≥ 1238ms
Deep breathing fractional area change	≤ 31%	----
Liver volume (S8-S11)	≤ 1.93L	----
Liver fat (S8-S11)	≥ 4.8%	----
Liver inflammation (S8-S11)	≥ 784 ms	----
Pancreatic fat (S12-S13)	≥ 4.6%	----
Pancreatic inflammation (S12-13)	≥ 803ms	----
Renal Cortical T1(S14-S15)	≥ 1227ms	≥ 1652ms
Spleen volume(S16)	≤ 0.35L	----

For peer review only - http://bmjopen.bmj.com/site/about/guidelines.xhtml

Table S2: Blood investigations in 201 low-risk individuals with post-COVID syndrome, sub-divided by those who were hospitalised versus those who were managed at home

Measurement	All	Managed at home	Hospitalised	p-value
Haemoglobin				
• Normal (130 - 170 g/L in men; 115 - 155 g/L in women)	170 (95.5%)	140 (95.9%)	30 (93.8%)	0.575
• Abnormal low (< 130 g/L in men; < 115 g/L in women)	5 (2.8%)	4 (2.7%)	1 (3.1%)	
• Abnormal high (> 170 g/L in men; > 155 g/L in women)	3 (1.7%)	2 (1.4%)	1 (3.1%)	
Haematocrit (HCT)				
• Normal (0.37 - 0.5 in men; 0.33 - 0.45 in women)	173 (97.2%)	142 (97.3%)	31 (96.9%)	0.386
• Abnormal low (< 0.37 in men; < 0.33 in women)	2 (1.1%)	1 (0.7%)	1 (3.1%)	
• Abnormal high (> 0.5 in men; > 0.45 in women)	3 (1.7%)	3 (2.1%)	0 (0%)	
Red cell count				
• Normal (4.4 - 5.8 x10^12/L in men; 3.95 - 5.15 x10^12/L in women)	170 (95.5%)	140 (95.9%)	30 (93.8%)	0.287
• Abnormal low (< 4.4 x10^12/L in men; < 3.95 x10^12/L in women)	5 (2.8%)	3 (2.1%)	2 (6.2%)	
• Abnormal high (> 5.8 x10^12/L in men; > 5.15 x10^12/L in women)	3 (1.7%)	3 (2.1%)	0 (0%)	
Mean cell volume (MCV)				
• Normal (80 - 99 fL)	174 (97.8%)	142 (97.3%)	32 (100%)	1
• Abnormal low (< 80 fL)	4 (2.2%)	4 (2.7%)	0 (0%)	
• Abnormal high (> 99 fL)	0 (0%)	0 (0%)	0 (0%)	
Mean corpuscular haemoglobin (MCH)				
• Normal (26 - 33.5 pg)	174 (97.8%)	143 (97.9%)	31 (96.9%)	0.249
• Abnormal low (< 26 pg)	3 (1.7%)	3 (2.1%)	0 (0%)	
• Abnormal high (> 33.5 pg)	1 (0.6%)	0 (0%)	1 (3.1%)	
Mean corpuscular haemoglobin concentration (MCHC)				
• Normal (300 - 350 g/L)	135 (75.8%)	109 (74.7%)	26 (81.2%)	0.501
• Abnormal low (< 300 g/L)	0 (0%)	0 (0%)	0 (0%)	
• Abnormal high (> 350 g/L)	43 (24.2%)	37 (25.3%)	6 (18.8%)	
Red cell distribution width (RDW)				
• Normal (11.5 - 15)	161 (91%)	129 (89%)	32 (100%)	0.218
• Abnormal low (< 11.5)	10 (5.6%)	10 (6.9%)	0 (0%)	
• Abnormal high (> 15)	6 (3.4%)	6 (4.1%)	0 (0%)	
Platelet count				
• Normal (150 - 400 x10^9/L)	166 (93.3%)	138 (94.5%)	28 (87.5%)	0.152
• Abnormal low (< 150 x10^9/L)	2 (1.1%)	2 (1.4%)	0 (0%)	
• Abnormal high (> 400 x10^9/L)	10 (5.6%)	6 (4.1%)	4 (12.5%)	
Mean platelet volume (MPV)				
• Normal (7 - 13 fL)	177 (99.4%)	145 (99.3%)	32 (100%)	1
• Abnormal low (< 7 fL)	0 (0%)	0 (0%)	0 (0%)	
• Abnormal high (> 13 fL)	1 (0.6%)	1 (0.7%)	0 (0%)	
White cell count				

For peer review only - http://bmjopen.bmj.com/site/about/guidelines.xhtml

• Normal (3 - 10 x10^9/L)	172 (96.6%)	140 (95.9%)	32 (100%)	0.593
• Abnormal low (< 3 x10^9/L)	0 (0%)	0 (0%)	0 (0%)	
• Abnormal high (> 10 x10^9/L)	6 (3.4%)	6 (4.1%)	0 (0%)	
Neutrophils				
• Normal (2 - 7.5 x10^9/L)	163 (91.6%)	133 (91.1%)	30 (93.8%)	1
• Abnormal low (< 2 x10^9/L)	12 (6.7%)	10 (6.8%)	2 (6.2%)	
• Abnormal high (> 7.5 x10^9/L)	3 (1.7%)	3 (2.1%)	0 (0%)	
Lymphocytes				
• Normal (1.2 - 3.65 x10^9/L)	161 (90.4%)	130 (89%)	31 (96.9%)	0.316
• Abnormal low (< 1.2 x10^9/L)	17 (9.6%)	16 (11%)	1 (3.1%)	
• Abnormal high (> 3.65 x10^9/L)	0 (0%)	0 (0%)	0 (0%)	
Monocytes				
• Normal (0.2 - 1 x10^9/L)	176 (98.9%)	144 (98.6%)	32 (100%)	1
• Abnormal low (< 0.2 x10^9/L)	1 (0.6%)	1 (0.7%)	0 (0%)	
• Abnormal high (> 1 x10^9/L)	1 (0.6%)	1 (0.7%)	0 (0%)	
Eosinophils				
• Normal (0 - 0.4 x10^9/L)	172 (96.6%)	141 (96.6%)	31 (96.9%)	1
• Abnormal low (< 0 x10^9/L)	0 (0%)	0 (0%)	0 (0%)	
• Abnormal high (> 0.4 x10^9/L)	6 (3.4%)	5 (3.4%)	1 (3.1%)	
Basophils				
• Normal (0 - 0.1 x10^9/L)	178 (100%)	146 (100%)	32 (100%)	N/A
• Abnormal low (< 0 x10^9/L)	0 (0%)	0 (0%)	0 (0%)	
• Abnormal high (> 0.1 x10^9/L)	0 (0%)	0 (0%)	0 (0%)	
Erythrocyte sedimentation rate (ESR)				
• Normal (1 - 20 mm/hr)	164 (91.1%)	136 (91.9%)	28 (87.5%)	0.491
• Abnormal low (< 1 mm/hr)	0 (0%)	0 (0%)	0 (0%)	
• Abnormal high (> 20 mm/hr)	16 (8.9%)	12 (8.1%)	4 (12.5%)	
Sodium				
• Normal (135 - 145 mmol/L)	173 (97.2%)	141 (96.6%)	32 (100%)	1
• Abnormal low (< 135 mmol/L)	4 (2.2%)	4 (2.7%)	0 (0%)	
• Abnormal high (> 145 mmol/L)	1 (0.6%)	1 (0.7%)	0 (0%)	
Potassium				
• Normal (3.5 - 5.1 mmol/L)	108 (62.1%)	87 (61.3%)	21 (65.6%)	0.692
• Abnormal low (< 3.5 mmol/L)	0 (0%)	0 (0%)	0 (0%)	
• Abnormal high (> 5.1 mmol/L)	66 (37.9%)	55 (38.7%)	11 (34.4%)	
Chloride				
• Normal (98 - 107 mmol/L)	171 (96.1%)	139 (95.2%)	32 (100%)	1
• Abnormal low (< 98 mmol/L)	4 (2.2%)	4 (2.7%)	0 (0%)	
• Abnormal high (> 107 mmol/L)	3 (1.7%)	3 (2.1%)	0 (0%)	
Bicarbonate				
• Normal (22 - 29 mmol/L)	150 (84.3%)	125 (85.6%)	25 (78.1%)	0.169
• Abnormal low (< 22 mmol/L)	18 (10.1%)	15 (10.3%)	3 (9.4%)	
• Abnormal high (> 29 mmol/L)	10 (5.6%)	6 (4.1%)	4 (12.5%)	
Urea				

• Normal (1.7 - 8.3 mmol/L)	178 (100%)	146 (100%)	32 (100%)	N/A
• Abnormal low (< 1.7 mmol/L)	0 (0%)	0 (0%)	0 (0%)	
• Abnormal high (> 8.3 mmol/L)	0 (0%)	0 (0%)	0 (0%)	
Creatinine				
• Normal (66 - 112 umol/L in men; 49 - 92 umol/L in women)	161 (90.4%)	134 (91.8%)	27 (84.4%)	0.219
• Abnormal low (< 66 umol/L in men; < 49 umol/L in women)	12 (6.7%)	9 (6.2%)	3 (9.4%)	
• Abnormal high (> 112 umol/L in men; > 92 umol/L in women)	5 (2.8%)	3 (2.1%)	2 (6.2%)	
Bilirubin				
• Normal (0 - 20 umol/L)	175 (98.3%)	144 (98.6%)	31 (96.9%)	0.45
• Abnormal low (< 0 umol/L)	0 (0%)	0 (0%)	0 (0%)	
• Abnormal high (> 20 umol/L)	3 (1.7%)	2 (1.4%)	1 (3.1%)	
Alkaline phosphatase				
• Normal (40 - 129 IU/L in men; 35 - 104 IU/L in women)	168 (94.4%)	137 (93.8%)	31 (96.9%)	0.161
• Abnormal low (< 40 IU/L in men; < 35 IU/L in women)	8 (4.5%)	8 (5.5%)	0 (0%)	
• Abnormal high (> 129 IU/L in men; > 104 IU/L in women)	2 (1.1%)	1 (0.7%)	1 (3.1%)	
Aspartate transferase				
• Normal (0 - 37 IU/L in men; 0 - 31 IU/L in women)	162 (93.1%)	133 (93.7%)	29 (90.6%)	0.464
• Abnormal low (< 0 IU/L in men; < 0 IU/L in women)	0 (0%)	0 (0%)	0 (0%)	
• Abnormal high (> 37 IU/L in men; > 31 IU/L in women)	12 (6.9%)	9 (6.3%)	3 (9.4%)	
Alanine transferase				
• Normal (10 - 50 IU/L in men; 10 - 35 IU/L in women)	151 (84.8%)	125 (85.6%)	26 (81.2%)	0.603
• Abnormal low (< 10 IU/L in men; < 10 IU/L in women)	2 (1.1%)	2 (1.4%)	0 (0%)	
• Abnormal high (> 50 IU/L in men; > 35 IU/L in women)	25 (14%)	19 (13%)	6 (18.8%)	
Lactate dehydrogenase (LDH)				
• Normal (135 - 225 IU/L in men; 135 - 214 IU/L in women)	142 (80.7%)	118 (81.9%)	24 (75%)	0.236
• Abnormal low (< 135 IU/L in men; < 135 IU/L in women)	5 (2.8%)	5 (3.5%)	0 (0%)	
• Abnormal high (> 225 IU/L in men; > 214 IU/L in women)	29 (16.5%)	21 (14.6%)	8 (25%)	
Creatinine kinase (CK)				
• Normal (38 - 204 IU/L in men; 26 - 140 IU/L in women)	163 (91.6%)	132 (90.4%)	31 (96.9%)	0.642
• Abnormal low (< 38 IU/L in men; < 26 IU/L in women)	2 (1.1%)	2 (1.4%)	0 (0%)	
• Abnormal high (> 204 IU/L in men; > 140 IU/L in women)	13 (7.3%)	12 (8.2%)	1 (3.1%)	
Gamma glutamyl transferase				
• Normal (10 - 71 IU/L in men; 6 - 42 IU/L in women)	165 (92.7%)	136 (93.2%)	29 (90.6%)	0.461
• Abnormal low (< 10 IU/L in men; < 6 IU/L in women)	4 (2.2%)	4 (2.7%)	0 (0%)	
• Abnormal high (> 71 IU/L in men; > 42 IU/L in women)	9 (5.1%)	6 (4.1%)	3 (9.4%)	
Total protein				
• Normal (63 - 83 g/L)	173 (97.2%)	143 (97.9%)	30 (93.8%)	0.22
• Abnormal low (< 63 g/L)	3 (1.7%)	2 (1.4%)	1 (3.1%)	
• Abnormal high (> 83 g/L)	2 (1.1%)	1 (0.7%)	1 (3.1%)	
Albumin				
• Normal (34 - 50 g/L)	167 (93.8%)	136 (93.2%)	31 (96.9%)	0.692
• Abnormal low (< 34 g/L)	0 (0%)	0 (0%)	0 (0%)	
• Abnormal high (> 50 g/L)	11 (6.2%)	10 (6.8%)	1 (3.1%)	
Globulin				

For peer review only - http://bmjopen.bmj.com/site/about/guidelines.xhtml

• Normal (19 - 35 g/L)	173 (97.2%)	142 (97.3%)	31 (96.9%)	0.386
• Abnormal low (< 19 g/L)	3 (1.7%)	3 (2.1%)	0 (0%)	
• Abnormal high (> 35 g/L)	2 (1.1%)	1 (0.7%)	1 (3.1%)	
Calcium				
• Normal (2.2 - 2.6 mmol/L)	172 (96.6%)	141 (96.6%)	31 (96.9%)	0.43
• Abnormal low (< 2.2 mmol/L)	2 (1.1%)	1 (0.7%)	1 (3.1%)	
• Abnormal high (> 2.6 mmol/L)	4 (2.2%)	4 (2.7%)	0 (0%)	
Magnesium				
• Normal (0.6 - 1 mmol/L)	176 (98.9%)	144 (98.6%)	32 (100%)	1
• Abnormal low (< 0.6 mmol/L)	1 (0.6%)	1 (0.7%)	0 (0%)	
• Abnormal high (> 1 mmol/L)	1 (0.6%)	1 (0.7%)	0 (0%)	
Phosphate				
• Normal (0.87 - 1.45 mmol/L)	150 (84.3%)	121 (82.9%)	29 (90.6%)	0.518
• Abnormal low (< 0.87 mmol/L)	23 (12.9%)	21 (14.4%)	2 (6.2%)	
• Abnormal high (> 1.45 mmol/L)	5 (2.8%)	4 (2.7%)	1 (3.1%)	
Uric acid				
• Normal (266 - 474 umol/L in men; 175 - 363 umol/L in women)	148 (83.1%)	124 (84.9%)	24 (75%)	0.067
• Abnormal low (< 266 umol/L in men; < 175 umol/L in women)	19 (10.7%)	16 (11%)	3 (9.4%)	
• Abnormal high (> 474 umol/L in men; > 363 umol/L in women)	11 (6.2%)	6 (4.1%)	5 (15.6%)	
Triglycerides				
• Normal (< 2.3 mmol/L)	10 (100%)	8 (100%)	2 (100%)	N/A
• Abnormal high (> 2.3 mmol/L)	0 (0%)	0 (0%)	0 (0%)	
Fasting triglycerides				
• Normal (< 2.3 mmol/L)	149 (88.7%)	128 (92.8%)	21 (70%)	0.002
• Abnormal high (> 2.3 mmol/L)	19 (11.3%)	10 (7.2%)	9 (30%)	
Cholesterol				
• Normal (< 5 mmol/L)	4 (40%)	3 (37.5%)	1 (50%)	1
• Abnormal high (> 5 mmol/L)	6 (60%)	5 (62.5%)	1 (50%)	
Fasting cholesterol				
• Normal (< 5 mmol/L)	98 (58.3%)	86 (62.3%)	12 (40%)	0.04
• Abnormal high (> 5 mmol/L)	70 (41.7%)	52 (37.7%)	18 (60%)	
HDL cholesterol				
• Normal (0.9 - 1.5 mmol/L in men; 1.2 - 1.7 mmol/L in women)	106 (59.6%)	87 (59.6%)	19 (59.4%)	0.075
• Abnormal low (< 0.9 mmol/L in men; < 1.2 mmol/L in women)	16 (9%)	10 (6.8%)	6 (18.8%)	
• Abnormal high (> 1.5 mmol/L in men; > 1.7 mmol/L in women)	56 (31.5%)	49 (33.6%)	7 (21.9%)	
LDL cholesterol				
• Normal (< 3 mmol/L)	113 (64.9%)	100 (69.4%)	13 (43.3%)	0.011
• Abnormal high (> 3 mmol/L)	61 (35.1%)	44 (30.6%)	17 (56.7%)	
Iron				
• Normal (10.6 - 28.3 umol/L in men; 6.6 - 26 umol/L in women)	164 (92.1%)	135 (92.5%)	29 (90.6%)	0.22
• Abnormal low (< 10.6 umol/L in men; < 6.6 umol/L in women)	4 (2.2%)	2 (1.4%)	2 (6.2%)	

• Abnormal high (> 28.3 umol/L in men; > 26 umol/L in women)	10 (5.6%)	9 (6.2%)	1 (3.1%)	
Total iron binding capacity (TIBC)				
• Normal (41 - 77 umol/L)	172 (97.2%)	141 (97.2%)	31 (96.9%)	1
• Abnormal low (< 41 umol/L)	0 (0%)	0 (0%)	0 (0%)	
• Abnormal high (> 77 umol/L)	5 (2.8%)	4 (2.8%)	1 (3.1%)	
Transferrin saturation				
• Normal (20 - 55 %)	139 (78.5%)	120 (82.8%)	19 (59.4%)	0.011
• Abnormal low (< 20 %)	34 (19.2%)	22 (15.2%)	12 (37.5%)	
• Abnormal high (> 55 %)	4 (2.3%)	3 (2.1%)	1 (3.1%)	
High sensitivity CRP				
• Normal (0 - 5 mg/L)	146 (92.4%)	124 (93.9%)	22 (84.6%)	0.112
• Abnormal low (< 0 mg/L)	0 (0%)	0 (0%)	0 (0%)	
• Abnormal high (> 5 mg/L)	12 (7.6%)	8 (6.1%)	4 (15.4%)	

For peer review only

For peer review only - http://bmjopen.bmj.com/site/about/guidelines.xhtml

Table S3: Blood investigations in 201 low-risk individuals sub-divided by those with severe or moderate post-COVID syndrome (PCS)

Measurement	All	Moderate PCS	Severe PCS	p-value
Haemoglobin				
• Normal (130 - 170 g/L in men; 115 - 155 g/L in women)	166 (96%)	62 (96.9%)	104 (95.4%)	1
• Abnormal low (< 130 g/L in men; < 115 g/L in women)	4 (2.3%)	1 (1.6%)	3 (2.8%)	
• Abnormal high (> 170 g/L in men; > 155 g/L in women)	3 (1.7%)	1 (1.6%)	2 (1.8%)	
Haematocrit (HCT)				
• Normal (0.37 - 0.5 in men; 0.33 - 0.45 in women)	168 (97.1%)	64 (100%)	104 (95.4%)	0.274
• Abnormal low (< 0.37 in men; < 0.33 in women)	2 (1.2%)	0 (0%)	2 (1.8%)	
• Abnormal high (> 0.5 in men; > 0.45 in women)	3 (1.7%)	0 (0%)	3 (2.8%)	
Red cell count				
• Normal (4.4 - 5.8 x10^12/L in men; 3.95 - 5.15 x10^12/L in women)	167 (96.5%)	61 (95.3%)	106 (97.2%)	0.825
• Abnormal low (< 4.4 x10^12/L in men; < 3.95 x10^12/L in women)	4 (2.3%)	2 (3.1%)	2 (1.8%)	
• Abnormal high (> 5.8 x10^12/L in men; > 5.15 x10^12/L in women)	2 (1.2%)	1 (1.6%)	1 (0.9%)	
Mean cell volume (MCV)				
• Normal (80 - 99 fL)	170 (98.3%)	62 (96.9%)	108 (99.1%)	0.556
• Abnormal low (< 80 fL)	3 (1.7%)	2 (3.1%)	1 (0.9%)	
• Abnormal high (> 99 fL)	0 (0%)	0 (0%)	0 (0%)	
Mean corpuscular haemoglobin (MCH)				
• Normal (26 - 33.5 pg)	170 (98.3%)	61 (95.3%)	109 (100%)	0.049
• Abnormal low (< 26 pg)	2 (1.2%)	2 (3.1%)	0 (0%)	
• Abnormal high (> 33.5 pg)	1 (0.6%)	1 (1.6%)	0 (0%)	
Mean corpuscular haemoglobin concentration (MCHC)				
• Normal (300 - 350 g/L)	131 (75.7%)	53 (82.8%)	78 (71.6%)	0.103
• Abnormal low (< 300 g/L)	0 (0%)	0 (0%)	0 (0%)	
• Abnormal high (> 350 g/L)	42 (24.3%)	11 (17.2%)	31 (28.4%)	
Red cell distribution width (RDW)				
• Normal (11.5 - 15)	157 (91.3%)	59 (92.2%)	98 (90.7%)	0.339
• Abnormal low (< 11.5)	10 (5.8%)	2 (3.1%)	8 (7.4%)	
• Abnormal high (> 15)	5 (2.9%)	3 (4.7%)	2 (1.9%)	
Platelet count				
• Normal (150 - 400 x10^9/L)	161 (93.1%)	59 (92.2%)	102 (93.6%)	0.417
• Abnormal low (< 150 x10^9/L)	2 (1.2%)	0 (0%)	2 (1.8%)	
• Abnormal high (> 400 x10^9/L)	10 (5.8%)	5 (7.8%)	5 (4.6%)	
Mean platelet volume (MPV)				
• Normal (7 - 13 fL)	172 (99.4%)	64 (100%)	108 (99.1%)	1
• Abnormal low (< 7 fL)	0 (0%)	0 (0%)	0 (0%)	

• Abnormal high (> 13 fL)	1 (0.6%)	0 (0%)	1 (0.9%)	
White cell count				
• Normal (3 - 10 x10^9/L)	167 (96.5%)	61 (95.3%)	106 (97.2%)	0.671
• Abnormal low (< 3 x10^9/L)	0 (0%)	0 (0%)	0 (0%)	
• Abnormal high (> 10 x10^9/L)	6 (3.5%)	3 (4.7%)	3 (2.8%)	
Neutrophils				
• Normal (2 - 7.5 x10^9/L)	159 (91.9%)	57 (89.1%)	102 (93.6%)	0.468
• Abnormal low (< 2 x10^9/L)	11 (6.4%)	5 (7.8%)	6 (5.5%)	
• Abnormal high (> 7.5 x10^9/L)	3 (1.7%)	2 (3.1%)	1 (0.9%)	
Lymphocytes				
• Normal (1.2 - 3.65 x10^9/L)	156 (90.2%)	56 (87.5%)	100 (91.7%)	0.43
• Abnormal low (< 1.2 x10^9/L)	17 (9.8%)	8 (12.5%)	9 (8.3%)	
• Abnormal high (> 3.65 x10^9/L)	0 (0%)	0 (0%)	0 (0%)	
Monocytes				
• Normal (0.2 - 1 x10^9/L)	171 (98.8%)	63 (98.4%)	108 (99.1%)	0.604
• Abnormal low (< 0.2 x10^9/L)	1 (0.6%)	0 (0%)	1 (0.9%)	
• Abnormal high (> 1 x10^9/L)	1 (0.6%)	1 (1.6%)	0 (0%)	
Eosinophils				
• Normal (0 - 0.4 x10^9/L)	167 (96.5%)	63 (98.4%)	104 (95.4%)	0.415
• Abnormal low (< 0 x10^9/L)	0 (0%)	0 (0%)	0 (0%)	
• Abnormal high (> 0.4 x10^9/L)	6 (3.5%)	1 (1.6%)	5 (4.6%)	
Basophils				
• Normal (0 - 0.1 x10^9/L)	173 (100%)	64 (100%)	109 (100%)	N/A
• Abnormal low (< 0 x10^9/L)	0 (0%)	0 (0%)	0 (0%)	
• Abnormal high (> 0.1 x10^9/L)	0 (0%)	0 (0%)	0 (0%)	
Erythrocyte sedimentation rate (ESR)				
• Normal (1 - 20 mm/hr)	160 (91.4%)	62 (93.9%)	98 (89.9%)	0.416
• Abnormal low (< 1 mm/hr)	0 (0%)	0 (0%)	0 (0%)	
• Abnormal high (> 20 mm/hr)	15 (8.6%)	4 (6.1%)	11 (10.1%)	
Sodium				
• Normal (135 - 145 mmol/L)	168 (97.1%)	63 (98.4%)	105 (96.3%)	1
• Abnormal low (< 135 mmol/L)	4 (2.3%)	1 (1.6%)	3 (2.8%)	
• Abnormal high (> 145 mmol/L)	1 (0.6%)	0 (0%)	1 (0.9%)	
Potassium				
• Normal (3.5 - 5.1 mmol/L)	105 (62.1%)	35 (56.5%)	70 (65.4%)	0.255
• Abnormal low (< 3.5 mmol/L)	0 (0%)	0 (0%)	0 (0%)	
• Abnormal high (> 5.1 mmol/L)	64 (37.9%)	27 (43.5%)	37 (34.6%)	
Chloride				
• Normal (98 - 107 mmol/L)	166 (96%)	62 (96.9%)	104 (95.4%)	1
• Abnormal low (< 98 mmol/L)	4 (2.3%)	1 (1.6%)	3 (2.8%)	
• Abnormal high (> 107 mmol/L)	3 (1.7%)	1 (1.6%)	2 (1.8%)	

For peer review only - http://bmjopen.bmj.com/site/about/guidelines.xhtml

Bicarbonate				
• Normal (22 - 29 mmol/L)	147 (85%)	55 (85.9%)	92 (84.4%)	0.946
• Abnormal low (< 22 mmol/L)	16 (9.2%)	6 (9.4%)	10 (9.2%)	
• Abnormal high (> 29 mmol/L)	10 (5.8%)	3 (4.7%)	7 (6.4%)	
Urea				
• Normal (1.7 - 8.3 mmol/L)	173 (100%)	64 (100%)	109 (100%)	N/A
• Abnormal low (< 1.7 mmol/L)	0 (0%)	0 (0%)	0 (0%)	
• Abnormal high (> 8.3 mmol/L)	0 (0%)	0 (0%)	0 (0%)	
Creatinine				
• Normal (66 - 112 umol/L in men; 49 - 92 umol/L in women)	156 (90.2%)	59 (92.2%)	97 (89%)	0.705
• Abnormal low (< 66 umol/L in men; < 49 umol/L in women)	12 (6.9%)	3 (4.7%)	9 (8.3%)	
• Abnormal high (> 112 umol/L in men; > 92 umol/L in women)	5 (2.9%)	2 (3.1%)	3 (2.8%)	
Bilirubin				
• Normal (0 - 20 umol/L)	170 (98.3%)	63 (98.4%)	107 (98.2%)	1
• Abnormal low (< 0 umol/L)	0 (0%)	0 (0%)	0 (0%)	
• Abnormal high (> 20 umol/L)	3 (1.7%)	1 (1.6%)	2 (1.8%)	
Alkaline phosphatase				
• Normal (40 - 129 IU/L in men; 35 - 104 IU/L in women)	164 (94.8%)	59 (92.2%)	105 (96.3%)	0.185
• Abnormal low (< 40 IU/L in men; < 35 IU/L in women)	7 (4%)	3 (4.7%)	4 (3.7%)	
• Abnormal high (> 129 IU/L in men; > 104 IU/L in women)	2 (1.2%)	2 (3.1%)	0 (0%)	
Aspartate transferase				
• Normal (0 - 37 IU/L in men; 0 - 31 IU/L in women)	157 (92.9%)	59 (93.7%)	98 (92.5%)	1
• Abnormal low (< 0 IU/L in men; < 0 IU/L in women)	0 (0%)	0 (0%)	0 (0%)	
• Abnormal high (> 37 IU/L in men; > 31 IU/L in women)	12 (7.1%)	4 (6.3%)	8 (7.5%)	
Alanine transferase				
• Normal (10 - 50 IU/L in men; 10 - 35 IU/L in women)	146 (84.4%)	56 (87.5%)	90 (82.6%)	0.512
• Abnormal low (< 10 IU/L in men; < 10 IU/L in women)	2 (1.2%)	1 (1.6%)	1 (0.9%)	
• Abnormal high (> 50 IU/L in men; > 35 IU/L in women)	25 (14.5%)	7 (10.9%)	18 (16.5%)	
Lactate dehydrogenase (LDH)				
• Normal (135 - 225 IU/L in men; 135 - 214 IU/L in women)	137 (80.1%)	51 (81%)	86 (79.6%)	0.24
• Abnormal low (< 135 IU/L in men; < 135 IU/L in women)	5 (2.9%)	0 (0%)	5 (4.6%)	
• Abnormal high (> 225 IU/L in men; > 214 IU/L in women)	29 (17%)	12 (19%)	17 (15.7%)	
Creatinine kinase (CK)				
• Normal (38 - 204 IU/L in men; 26 - 140 IU/L in women)	159 (91.9%)	56 (87.5%)	103 (94.5%)	0.28
• Abnormal low (< 38 IU/L in men; < 26 IU/L in women)	2 (1.2%)	1 (1.6%)	1 (0.9%)	
• Abnormal high (> 204 IU/L in men; > 140 IU/L in women)	12 (6.9%)	7 (10.9%)	5 (4.6%)	
Gamma glutamyl transferase				
• Normal (10 - 71 IU/L in men; 6 - 42 IU/L in women)	161 (93.1%)	60 (93.8%)	101 (92.7%)	0.426
• Abnormal low (< 10 IU/L in men; < 6 IU/L in women)	3 (1.7%)	0 (0%)	3 (2.8%)	
• Abnormal high (> 71 IU/L in men; > 42 IU/L in women)	9 (5.2%)	4 (6.2%)	5 (4.6%)	
Total protein				
• Normal (63 - 83 g/L)	168 (97.1%)	63 (98.4%)	105 (96.3%)	0.792

• Abnormal low (< 63 g/L)	3 (1.7%)	1 (1.6%)	2 (1.8%)	
• Abnormal high (> 83 g/L)	2 (1.2%)	0 (0%)	2 (1.8%)	
Albumin				
• Normal (34 - 50 g/L)	162 (93.6%)	59 (92.2%)	103 (94.5%)	0.538
• Abnormal low (< 34 g/L)	0 (0%)	0 (0%)	0 (0%)	
• Abnormal high (> 50 g/L)	11 (6.4%)	5 (7.8%)	6 (5.5%)	
Globulin				
• Normal (19 - 35 g/L)	168 (97.1%)	61 (95.3%)	107 (98.2%)	0.616
• Abnormal low (< 19 g/L)	3 (1.7%)	2 (3.1%)	1 (0.9%)	
• Abnormal high (> 35 g/L)	2 (1.2%)	1 (1.6%)	1 (0.9%)	
Calcium				
• Normal (2.2 - 2.6 mmol/L)	167 (96.5%)	62 (96.9%)	105 (96.3%)	0.525
• Abnormal low (< 2.2 mmol/L)	2 (1.2%)	0 (0%)	2 (1.8%)	
• Abnormal high (> 2.6 mmol/L)	4 (2.3%)	2 (3.1%)	2 (1.8%)	
Magnesium				
• Normal (0.6 - 1 mmol/L)	171 (98.8%)	63 (98.4%)	108 (99.1%)	0.604
• Abnormal low (< 0.6 mmol/L)	1 (0.6%)	1 (1.6%)	0 (0%)	
• Abnormal high (> 1 mmol/L)	1 (0.6%)	0 (0%)	1 (0.9%)	
Phosphate				
• Normal (0.87 - 1.45 mmol/L)	145 (83.8%)	55 (85.9%)	90 (82.6%)	0.824
• Abnormal low (< 0.87 mmol/L)	23 (13.3%)	8 (12.5%)	15 (13.8%)	
• Abnormal high (> 1.45 mmol/L)	5 (2.9%)	1 (1.6%)	4 (3.7%)	
Uric acid				
• Normal (266 - 474 umol/L in men; 175 - 363 umol/L in women)	145 (83.8%)	53 (82.8%)	92 (84.4%)	0.804
• Abnormal low (< 266 umol/L in men; < 175 umol/L in women)	18 (10.4%)	8 (12.5%)	10 (9.2%)	
• Abnormal high (> 474 umol/L in men; > 363 umol/L in women)	10 (5.8%)	3 (4.7%)	7 (6.4%)	
Triglycerides				
• Normal (< 2.3 mmol/L)	10 (100%)	6 (100%)	4 (100%)	N/A
• Abnormal high (> 2.3 mmol/L)	0 (0%)	0 (0%)	0 (0%)	
Fasting triglycerides				
• Normal (< 2.3 mmol/L)	144 (88.3%)	52 (89.7%)	92 (87.6%)	0.802
• Abnormal high (> 2.3 mmol/L)	19 (11.7%)	6 (10.3%)	13 (12.4%)	
Cholesterol				
• Normal (< 5 mmol/L)	4 (40%)	3 (50%)	1 (25%)	0.571
• Abnormal high (> 5 mmol/L)	6 (60%)	3 (50%)	3 (75%)	
Fasting cholesterol				
• Normal (< 5 mmol/L)	96 (58.9%)	39 (67.2%)	57 (54.3%)	0.135
• Abnormal high (> 5 mmol/L)	67 (41.1%)	19 (32.8%)	48 (45.7%)	
HDL cholesterol				
• Normal (0.9 - 1.5 mmol/L in men; 1.2 - 1.7 mmol/L in women)	103 (59.5%)	38 (59.4%)	65 (59.6%)	0.539
• Abnormal low (< 0.9 mmol/L in men; < 1.2 mmol/L in women)	16 (9.2%)	4 (6.2%)	12 (11%)	

• Abnormal high (> 1.5 mmol/L in men; > 1.7 mmol/L in women)	54 (31.2%)	22 (34.4%)	32 (29.4%)	
**LDL cholesterol**				
• Normal (< 3 mmol/L)	111 (65.7%)	45 (72.6%)	66 (61.7%)	0.18
• Abnormal high (> 3 mmol/L)	58 (34.3%)	17 (27.4%)	41 (38.3%)	
**Iron**				
• Normal (10.6 - 28.3 umol/L in men; 6.6 - 26 umol/L in women)	160 (92.5%)	57 (89.1%)	103 (94.5%)	0.337
• Abnormal low (< 10.6 umol/L in men; < 6.6 umol/L in women)	3 (1.7%)	2 (3.1%)	1 (0.9%)	
• Abnormal high (> 28.3 umol/L in men; > 26 umol/L in women)	10 (5.8%)	5 (7.8%)	5 (4.6%)	
**Total iron binding capacity (TIBC)**				
• Normal (41 - 77 umol/L)	167 (97.1%)	60 (93.8%)	107 (99.1%)	0.064
• Abnormal low (< 41 umol/L)	0 (0%)	0 (0%)	0 (0%)	
• Abnormal high (> 77 umol/L)	5 (2.9%)	4 (6.2%)	1 (0.9%)	
**Transferrin saturation**				
• Normal (20 - 55 %)	135 (78.5%)	50 (78.1%)	85 (78.7%)	0.283
• Abnormal low (< 20 %)	33 (19.2%)	11 (17.2%)	22 (20.4%)	
• Abnormal high (> 55 %)	4 (2.3%)	3 (4.7%)	1 (0.9%)	
**High sensitivity CRP**				
• Normal (0 - 5 mg/L)	141 (92.2%)	50 (96.2%)	91 (90.1%)	0.223
• Abnormal low (< 0 mg/L)	0 (0%)	0 (0%)	0 (0%)	
• Abnormal high (> 5 mg/L)	12 (7.8%)	2 (3.8%)	10 (9.9%)	

eview only

For peer review only - http://bmjopen.bmj.com/site/about/guidelines.xhtml

STROBE Statement—checklist of items that should be included in reports of observational studies

	Item No	Recommendation	Pages
**Title and abstract**	1	(*a*) Indicate the study's design with a commonly used term in the title or the abstract	3
		(*b*) Provide in the abstract an informative and balanced summary of what was done and what was found	3
**Introduction**			
Background/rationale	2	Explain the scientific background and rationale for the investigation being reported	4
Objectives	3	State specific objectives, including any prespecified hypotheses	4
**Methods**			
Study design	4	Present key elements of study design early in the paper	5
Setting	5	Describe the setting, locations, and relevant dates, including periods of recruitment, exposure, follow-up, and data collection	5
Participants	6	(*a*) *Cohort study*—Give the eligibility criteria, and the sources and methods of selection of participants. Describe methods of follow-up<br>*Case-control study*—Give the eligibility criteria, and the sources and methods of case ascertainment and control selection. Give the rationale for the choice of cases and controls<br>*Cross-sectional study*—Give the eligibility criteria, and the sources and methods of selection of participants	5
		(*b*) *Cohort study*—For matched studies, give matching criteria and number of exposed and unexposed<br>*Case-control study*—For matched studies, give matching criteria and the number of controls per case	3
Variables	7	Clearly define all outcomes, exposures, predictors, potential confounders, and effect modifiers. Give diagnostic criteria, if applicable	8
Data sources/ measurement	8*	For each variable of interest, give sources of data and details of methods of assessment (measurement). Describe comparability of assessment methods if there is more than one group	5-6 and supplementary
Bias	9	Describe any efforts to address potential sources of bias	10
Study size	10	Explain how the study size was arrived at	Ongoing study
Quantitative variables	11	Explain how quantitative variables were handled in the analyses. If applicable, describe which groupings were chosen and why	6
Statistical methods	12	(*a*) Describe all statistical methods, including those used to control for confounding	6
		(*b*) Describe any methods used to examine subgroups and interactions	6
		(*c*) Explain how missing data were addressed	10
		(*d*) *Cohort study*—If applicable, explain how loss to follow-up was addressed	n/a ongoing observational study

For peer review only - http://bmjopen.bmj.com/site/about/guidelines.xhtml

		Case-control study—If applicable, explain how matching of cases and controls was addressed<br>*Cross-sectional study*—If applicable, describe analytical methods taking account of sampling strategy	
		(*e*) Describe any sensitivity analyses	n/a
**Results**			
Participants	13*	(a) Report numbers of individuals at each stage of study—eg numbers potentially eligible, examined for eligibility, confirmed eligible, included in the study, completing follow-up, and analysed	7<br>Figure 1
		(b) Give reasons for non-participation at each stage	Figure 1
		(c) Consider use of a flow diagram	Figure 1
Descriptive data	14*	(a) Give characteristics of study participants (eg demographic, clinical, social) and information on exposures and potential confounders	Table 1
		(b) Indicate number of participants with missing data for each variable of interest	Table 1
		(c) *Cohort study*—Summarise follow-up time (eg, average and total amount)	Table 1
Outcome data	15*	*Cohort study*—Report numbers of outcome events or summary measures over time	Table 1
		Case-control study—Report numbers in each exposure category, or summary measures of exposure	Table 1
		Cross-sectional study—Report numbers of outcome events or summary measures	n/a
Main results	16	(*a*) Give unadjusted estimates and, if applicable, confounder-adjusted estimates and their precision (eg, 95% confidence interval). Make clear which confounders were adjusted for and why they were included	8
		(*b*) Report category boundaries when continuous variables were categorized	Table 2
		(*c*) If relevant, consider translating estimates of relative risk into absolute risk for a meaningful time period	n/a
Other analyses	17	Report other analyses done—eg analyses of subgroups and interactions, and sensitivity analyses	n/a
**Discussion**			
Key results	18	Summarise key results with reference to study objectives	9
Limitations	19	Discuss limitations of the study, taking into account sources of potential bias or imprecision. Discuss both direction and magnitude of any potential bias	10
Interpretation	20	Give a cautious overall interpretation of results considering objectives, limitations, multiplicity of analyses, results from similar studies, and other relevant evidence	10
Generalisability	21	Discuss the generalisability (external validity) of the study results	10
**Other information**			
Funding	22	Give the source of funding and the role of the funders for the present study and, if applicable, for the original study on which the present article is based	12

For peer review only - http://bmjopen.bmj.com/site/about/guidelines.xhtml

*Give information separately for cases and controls in case-control studies and, if applicable, for exposed and unexposed groups in cohort and cross-sectional studies.

Note: An Explanation and Elaboration article discusses each checklist item and gives methodological background and published examples of transparent reporting. The STROBE checklist is best used in conjunction with this article (freely available on the Web sites of PLoS Medicine at http://www.plosmedicine.org/, Annals of Internal Medicine at http://www.annals.org/, and Epidemiology at http://www.epidem.com/). Information on the STROBE Initiative is available at www.strobe-statement.org.

For peer review only

For peer review only - http://bmjopen.bmj.com/site/about/guidelines.xhtml

BMJ Open

Prospective, community-based study of multi-organ impairment in low-risk individuals with post-COVID syndrome

Journal:	*BMJ Open*
Manuscript ID	bmjopen-2020-048391.R1
Article Type:	Original research
Date Submitted by the Author:	25-Feb-2021
Complete List of Authors:	Dennis, Andrea; Perspectum Diagnostics Ltd, Innovation<br>Wamil, Malgorzata ; Great Western Hospital Foundation NHS Trust, Department of Cardiology<br>Alberts, Johann; Alliance Medical Limited<br>Oben, Jude; Guy's and St Thomas' NHS Foundation Trust, Department of Gastroenterology<br>Cuthbertson, Daniel; University of Liverpool, Institute of Cardiovascular and Metabolic Medicine<br>Wootton, Dan; University of Liverpool Institute of Infection and Global Health; Liverpool University Hospitals NHS Foundation Trust, Department of Respiratory Research<br>Crooks, Michael; Hull and East Yorkshire Hospitals NHS Trust, Department of Respiratory Medicine<br>Gabbay, Mark; University of Liverpool, 12Institute of Population Health Sciences<br>Brady, MIchael; Perspectum Diagnostics Ltd<br>Hishmeh, Lyth; Long COVID SOS<br>Attree, Emily; UKDoctors#Longcovid<br>Heightman, Melissa; University College London Hospitals NHS Foundation Trust, Department of Medicine<br>Banerjee, Rajarshi; Perspectum Diagnostics Ltd<br>Banerjee, Amitava; University College London, Institute of Health Informatics
**Primary Subject Heading**:	Health services research
Secondary Subject Heading:	Diagnostics, Epidemiology, Infectious diseases, Patient-centred medicine
Keywords:	COVID-19, EPIDEMIOLOGY, Health policy < HEALTH SERVICES ADMINISTRATION & MANAGEMENT, Public health < INFECTIOUS DISEASES, PUBLIC HEALTH

SCHOLARONE™
Manuscripts

I, the Submitting Author has the right to grant and does grant on behalf of all authors of the Work (as defined in the below author licence), an exclusive licence and/or a non-exclusive licence for contributions from authors who are: i) UK Crown employees; ii) where BMJ has agreed a CC-BY licence shall apply, and/or iii) in accordance with the terms applicable for US Federal Government officers or employees acting as part of their official duties; on a worldwide, perpetual, irrevocable, royalty-free basis to BMJ Publishing Group Ltd ("BMJ") its licensees and where the relevant Journal is co-owned by BMJ to the co-owners of the Journal, to publish the Work in this journal and any other BMJ products and to exploit all rights, as set out in our licence.

The Submitting Author accepts and understands that any supply made under these terms is made by BMJ to the Submitting Author unless you are acting as an employee on behalf of your employer or a postgraduate student of an affiliated institution which is paying any applicable article publishing charge ("APC") for Open Access articles. Where the Submitting Author wishes to make the Work available on an Open Access basis (and intends to pay the relevant APC), the terms of reuse of such Open Access shall be governed by a Creative Commons licence – details of these licences and which Creative Commons licence will apply to this Work are set out in our licence referred to above.

Other than as permitted in any relevant BMJ Author's Self Archiving Policies, I confirm this Work has not been accepted for publication elsewhere, is not being considered for publication elsewhere and does not duplicate material already published. I confirm all authors consent to publication of this Work and authorise the granting of this licence.

For peer review only

Prospective, community-based study of multi-organ impairment in low-risk individuals with post-COVID syndrome

Dennis A, Wamil M, Alberts J, Oben J, Cuthbertson D, Wootton D, Crooks M, Gabbay M, Brady M, Hishmeh L, Attree E, Heightman M, Banerjee R*, Banerjee A*

Andrea Dennis PhD[1], *Head of Biomarker Science, Perspectum* andrea.dennis@perspectum.com

Malgorzata Wamil PhD[2, 3] *Consultant Cardiologist* gosia.wamil@googlemail.com

Johann Alberts MBBCh[4], *Medical Director* jalberts@alliance.co.uk

Jude Oben PhD[5, 6], *Consultant Gastroenterologist/Hepatologist and Reader in Experimental Hepatology* jude.1.oben@kcl.ac.uk

Daniel Cuthbertson PhD[7, 8], *Professor of Diabetes and Consultant Physician* dan.cuthbertson@liverpool.ac.uk

Dan Wootton PhD[8, 9], *Senior Fellow and Consultant Respiratory Physician* D.G.Wootton@liverpool.ac.uk

Michael Crooks PhD [10, 11], *Senior Clinical Lecturer in Respiratory Medicine and Consultant Respiratory Physician* michael.crooks@hey.nhs.uk

Mark Gabbay PhD[12], *Professor of General Practice and General Practititoner* M.B.Gabbay@liverpool.ac.uk

Michael Brady PhD[1, 13], Professor of Oncological Imaging and Chair, Perspectum Michael.Brady@perspectum.com

Lyth Hishmeh BSc[14], Member, Long COVID SOS lythb7@hotmail.com

Emily Attree MBBS[15], *salaried general practitioner and Founder,* UKDoctors#Longcovid emily.attree@nhs.net

Melissa Heightman PhD[16], *Consultant Respiratory Physician* melissa.heightman1@nhs.net

Rajarshi Banerjee DPhil*[1,3], *Honorary Consultant Physician and Chief Executive, Perspectum* rajarshi.banerjee@perspectum.com

Amitava Banerjee DPhil*[16,17,18], *Associate Professor of Clinical Data Science and Honorary Consultant Cardiologist* ami.banerjee@ucl.ac.uk

On behalf of the COVERSCAN study investigators (listed at the end of manuscript)

[1]*Perspectum, 5520 John Smith Drive, Oxford, OX4 2LL, UK*

[2]*Great Western Hospitals NHS Foundation Trust*

[3]*Oxford University Hospitals NHS Foundation Trust*

[4]*Alliance Medical Limited, Iceni Centre, Warwick Technology Park, Warwick, CV34 6DA*

For peer review only - http://bmjopen.bmj.com/site/about/guidelines.xhtml

[5] *Guy's and St Thomas' NHS Foundation Trust, London*

[6] *Institute for Liver and Digestive Health, University College London*

[7] *Institute of Cardiovascular and Metabolic Medicine, University of Liverpool*

[8] *Liverpool University Hospitals NHS Foundation Trust, Liverpool*

[9] *Institute of Infection & Global Health, University of Liverpool*

[10] *Institute of Clinical and Applied Health Research, University of Hull*

[11] *Hull and East Yorkshire Hospitals NHS Trust, Hull.*

[12] *Institute of Population Health Sciences, University of Liverpool*

[13] *Department of Oncology, University of Oxford, Oxford.*

[14] *Long COVID SOS, Oxford, UK*

[15] *UKDoctors#Longcovid, London, UK*

[16] *University College London Hospitals NHS Trust, 235 Euston Road, London, UK*

[17] *Institute of Health Informatics, University College London, 222 Euston Road, London, UK*

[18] *Barts Health NHS Trust, The Royal London Hospital, Whitechapel Rd, London, UK*

*joint senior author

Corresponding authors: ami.banerjee@ucl.ac.uk; rajarshi.banerjee@perspectum.com

Abstract

Objective: To assess medium-term organ impairment in symptomatic individuals following recovery from acute SARS-CoV2 infection.

Design: Baseline findings from a prospective, observational cohort study.

Setting: Community-based individuals from two UK centres between 1 April and 14 September 2020.

Participants: Individuals ≥18 years with persistent symptoms following recovery from acute SARS-CoV-2 infection; and age-matched healthy controls.

Intervention: Assessment of symptoms by standardised questionnaires (EQ-5D-5L, Dyspnoea-12) and organ-specific metrics by biochemical assessment and quantitative magnetic resonance imaging (MRI).

For peer review only - http://bmjopen.bmj.com/site/about/guidelines.xhtml

Main outcome measures: Severe post-COVID syndrome defined as ongoing respiratory symptoms and/or moderate functional impairment in activities of daily living. Single and multi-organ impairment (heart, lungs, kidneys, liver, pancreas, spleen) by consensus definitions at baseline investigation.

Results: 201 individuals (mean age 45, range 21-71) years, 71% female, 88% white, 32% healthcare workers) completed baseline assessment (median 141 days following SARS-CoV-2 infection, IQR 110-162). The study population was low-risk for COVID-19 mortality (obesity: 20%, hypertension: 7%; type 2 diabetes: 2%; heart disease: 5%), with only 19% hospitalised with COVID-19. 42% of individuals had ten or more symptoms, and 60% had severe post-COVID syndrome. Fatigue (98%), muscle aches (87%), breathlessness (88%) and headaches (83%) were most frequently reported. Mild organ impairment was present in heart (26%), lungs (11%), kidneys (4%), liver (28%), pancreas (40%), and spleen (4%), with single and multi-organ impairment in 70% and 29% respectively. Hospitalisation was associated with older age ($p=0.001$), non-white ethnicity ($p=0.016$), increased liver volume ($p<0.0001$), pancreatic inflammation ($p<0.01$), and fat accumulation in the liver ($p<0.05$) and pancreas ($p<0.01$). Severe post-COVID syndrome was associated with radiological evidence of cardiac damage (myocarditis) ($p<0.05$).

Conclusions: In individuals at low risk of COVID-19 mortality with ongoing symptoms, 70% have impairment in one or more organs four months after initial COVID-19 symptoms, with implications for healthcare and public health, which have assumed low risk in young people with no comorbidities.

Study registration: https://clinicaltrials.gov/ct2/show/NCT04369807

For peer review only - http://bmjopen.bmj.com/site/about/guidelines.xhtml

Strengths and limitations

- This is an ongoing, prospective, longitudinal COVID-19 recovery study with biochemical and imaging characterisation of organ function, starting in April 2020 before recognition of "long-COVID", proper testing availability and prospective COVID-19-related research.
- By recruiting ambulatory patients with broad inclusion criteria, we focused on a real world population at lower risk of COVID-19 severity and mortality.
- Healthy controls were included for comparison, not individuals with post-flu symptoms, COVID-19 without symptoms or from general clinics, which further studies may explore.
- The study population was not ethnically diverse, despite disproportionate COVID-19 impact in non-white individuals.
- To limit interaction and exposure between trial team and patients, pulse oximetry, spirometry, MRI assessment of brain and muscle function were not included from the outset.

For peer review only

For peer review only

Introduction

Early in the COVID-19 pandemic, research and clinical practice focused on pulmonary manifestations[1]. There is increasing evidence for direct multi-organ effects[2-7], as well as indirect effects on other organ systems and disease processes, such as cardiovascular diseases and cancers, through changes in healthcare delivery and patient behaviours[8–10]. The clear long-term impact on individuals and health systems underlines the urgent need for a whole body approach with assessment of all major organ systems following SARS-CoV-2

For peer review only - http://bmjopen.bmj.com/site/about/guidelines.xhtml

(Severe acute respiratory syndrome-coronavirus 2) infection. Quantitative MRI has recently been used to show multi-organ impairment in individuals post-COVID-19 hospitalisation[11], but has not been used in non-hospitalised individuals.

COVID-19 is the convergence of an infectious disease, under-treated non-communicable diseases and social determinants of health, described as a “syndemic”[12]. Pre-existing non-communicable diseases and risk factors predict poor COVID-19 outcomes, whether intensive care admission or mortality[10]. Research has emphasised acute SARS-CoV-2 infection, hospitalised individuals, and COVID-19 mortality[13–15], which is likely to under-estimate the true burden of COVID-19-related disease. Among those surviving acute infection, 10% report persistent symptoms for 12 weeks or longer after initial infection (“long-COVID”, or “post COVID syndrome”, PCS)[16]. However, PCS is yet to be fully defined[17-20]. Neither severity of symptoms, nor medium- and long-term pathophysiology across organ systems, nor the appropriate control populations are understood.

UK government policies have emphasised excess mortality risk in moderate- and high-risk conditions, including “shielding”[10] and commissioning of a risk calculator to identify those at highest risk of COVID-19 severity and mortality[21]. These policies assume that younger individuals without apparent underlying conditions are at low risk. However, unlike symptoms following critical illness[22] or acute phase of other coronavirus infections[23], symptoms in PCS are commonly reported in individuals with low COVID-19 mortality risk, e.g. female, young and no chronic co-morbidities[14]. The potential scale of PCS in “lower-risk” individuals, representing up to 80% of the population[3], necessitates urgent policies across countries to

For peer review only

monitor[24], treat[19] and pay[25] for long-term implications of COVID-19, and to mitigate impact on healthcare utilisation and economies.

Therefore, in a pragmatic, prospective cohort study of individuals with persistent symptoms at least 4 weeks following recovery from acute SARS-CoV2 infection and at low risk of COVID-19 mortality, we investigated: (i) prevalence of multi-organ impairment, compared with healthy, age-matched controls; (ii) associations between typical COVID-19 symptoms and multi-organ impairment; and (iii) associations between hospitalisation, severity of symptoms and multi-organ impairment.

For peer review only

For peer review only - http://bmjopen.bmj.com/site/about/guidelines.xhtml

For peer review only

Methods

Patient population and study design

In an ongoing, prospective study, participants were recruited to the study following expressing their interest on the study registration website. Participants learnt about the study through advertisement on social media or via recommendation from clinicians from four Participant Identification Centres, the latter usually applied to patients who had been hospitalised. Assessment took place at two UK research imaging sites (Perspectum,

For peer review only - http://bmjopen.bmj.com/site/about/guidelines.xhtml

Oxford and Mayo Clinic Healthcare, London) between 1 April 2020 and 14 September 2020, completing baseline assessment by 14 September 2020 (**Figure 1**). Participants were eligible for enrolment with laboratory confirmed SAR-COV2 infection (tested SARS-CoV-2-positive by oro/nasopharyngeal swab by reverse-transcriptase-polymerase-chain reaction (n=62), a positive antibody test (n=63), or with strong clinical suspicion of infection with typical symptoms/signs and assessed as highly likely to have COVID-19 by two independent clinicians (n=73)). Exclusion criteria were: symptoms of active respiratory viral infection (temperature >37.8°C or three or more episodes of coughing in 24 hours); hospital discharge in the last 7 days; and contraindications to MRI, including: implanted pacemakers, defibrillators, other metallic implanted devices and claustrophobia. All participants gave written informed consent.

Assessment of post-COVID syndrome

Assessment included patient-reported validated questionnaires (quality of life, EQ-5D-5L[26], and dyspnoea-12[27]) and fasting biochemical investigations (listed in **Supplementary Methods**). PCS was classified as "severe" (defined as persistent breathlessness, ≥10 on the dyspnoea-12 score, or reported moderate or greater problems with usual activities on EQ-5D-5L), or "moderate". These thresholds were selected as the dyspnoea-12 has been correlated with the MRC dyspnoea grade, where level 3 warrants referral to rehabilitation services[27], and with EQ-5D-5L, less than 8% of the general population report moderate or greater problems with usual activities[28].

Multi-organ impairment in PCS compared with healthy controls

For peer review only

We selected MRI as the imaging modality (as in UK Biobank) due to: (1) safety (no radiation exposure, no need for intravenous contrast, and minimal contact with the radiographer); (2) quantitative reproducibility (>95% acquisition and image processing success rate); (3) capacity for information sharing (digital data repository for independent analysis and research); and (4) rapid scalability (35-minute scan to phenotype lung, heart, kidney, liver, pancreas and spleen). Multi-organ MRI data were collected at both study sites (Oxford: MAGNETOM Aera 1.5T, Mayo Healthcare London: MAGNETOM Vida 3T; both from Siemens Healthcare, Erlangen, Germany). The COVERSCAN multi-paramertic MRI assessment typically required 35 minutes per patient, including lungs, heart, liver, pancreas, kidneys and spleen by standardised methodology (**Supplementary methods**). In brief, we assessed inflammation of the heart, kidneys, liver and pancreas with quantitative T1-relaxation mapping, lung function was characterised with a dynamic structural T2-weighted lung scan estimating lung capacity, ectopic fat accumulation in the liver and pancreas from proton density fat fraction and volume of the liver and spleen measured from T1-weighted structural scan.

To determine impairment for each organ, we compared MRI-derived measurements from heart, lungs, kidney, liver, pancreas and spleen with reference ranges (**Table S1**), which were established as mean +/- 2 standard deviations from the healthy, age-matched control subjects (n=36), and validated by scoping literature review[11]. We defined organ impairment if quantitative T1 mapping was outside reference ranges for heart, kidney, liver and pancreas, reduced estimated lung capacity from dynamic measurements in the lungs or there was evidence of hepatomegaly, splenomegaly or ectopic fat accumulation.

Symptoms and multi-organ impairment

For peer review only - http://bmjopen.bmj.com/site/about/guidelines.xhtml

Associations between organ impairment and symptoms were visually assessed using a heat map dividing those with impairments to an organ into columns and colouring the rows by percentage of reported symptoms.

Hospitalisation, severity and multi-organ impairment

We compared mean differences in quantitative organ metrics for hospitalised versus not hospitalised and moderate versus severe PCS using Kruskal-Wallis test (Fisher's exact test for differences in binary outcomes). We defined multi-organ impairment as ≥2 organs with metrics outside the reference range. We investigated associations between multi-organ impairment and (i) being hospitalised and (ii) severe PCS with multivariate logistic regression models, adjusting for age, sex and BMI.

Patient and public involvement and engagement

Patients and public have directly, and indirectly, informed our research, from design to dissemination, with regular updates and webinars, including Q&A sessions with patients. Several clinician co-authors were indirectly informed by their patients in the COVERSCAN study (RB, AB) or PCS clinics (DW, MH, MC), who are members of organisations, such as Long Covid SOS (e.g. LH) and UKDoctors#Longcovid (e.g. EA). LH and EA have been involved in the research, interpretation of results, understanding implications of our results, and providing critical feedback for the manuscript.

Statistical analysis

We performed all analyses using R version 3.6.1, using descriptive statistics to summarise baseline characteristics, and considering a p-value less than 0.05 as statistically significant.

For peer review only - http://bmjopen.bmj.com/site/about/guidelines.xhtml

For peer review only

Mean and standard deviation (SD) were used for normally distributed-continuous, median with interquartile range (IQR) for non-normally distributed, and frequency and percentage for categorical variables, respectively. For group-wise comparison for absolute values between cases and healthy controls, we used Kruskal-Wallis test.

For peer review only

Results

Overall study population

Baseline characteristics

201 individuals were included (full details regarding hospitalisation: n=199; full questionnaire data to assign PCS severity: n=193). The mean age was 44.0 (range 21-71) years and median BMI 25.7 [IQR 23-28]. 71% of individuals were female, 88% were white, 32% were healthcare workers, 19% had been hospitalised with COVID-19. Assessment (symptoms, blood and MRI) was a median 141 (IQR 110-162) days after initial symptoms. Past medical history included smoking (3%), asthma (19%), obesity (20%), hypertension (7%), diabetes (2%) and prior heart disease (5%). The healthy control group had a mean age of 39 years (range 20-70), 40% were female and had a median BMI of 23 [IQR: 21-25] (**Table 1**).

Regardless of hospitalisation, the most frequently reported symptoms were fatigue (98%), shortness of breath (88%), muscle ache (87%), and headache (83%) (**Table 1).** 99% of individuals had four or more and 42% had ten or more symptoms. 70% of individuals reported ≥13 weeks off paid employment. Of the incidental structural findings observed on MRI (n=56),

For peer review only - http://bmjopen.bmj.com/site/about/guidelines.xhtml

three were cardiac (atrial septal defect, bicuspid aortic valve and right atrial mass), one renal (hydronephrosis), and the rest were benign cysts.

Haematological investigations including mean corpuscular haemoglobin concentration (MCHC, 24%), and renal, liver and lipid biochemistry, including potassium (38%), alanine transferase (14%), lactate dehydrogenase (17%), triglycerides (11%) and cholesterol (42%) were abnormally high in ≥10% of individuals. Bicarbonate (10%), phosphate (11%), uric acid (11%), and transferrin saturation (19%) were abnormally low in ≥10% of individuals (**Table S2**).

Single and multi-organ impairment in PCS compared with healthy controls

Organ impairment was more common in PCS than healthy controls (**Figure 2**, **Supplementary results, Figure S1**). Impairment was present in the heart in 26% (myocarditis 19%; systolic dysfunction 9%), lung in 11% (reduced vital capacity), kidney in 4% (inflammation), liver in 28% (12% inflammation; 21% ectopic fat, 10% hepatomegaly) and pancreas in 40% (15% inflammation, 38% ectopic fat); and spleen in 4% (splenomegaly). (**Table 2, Figure 2).** 70% of individuals had impairment in at least one organ. 29% of individuals had multi-organ impairment, with overlap across multiple organs (**Figure 3**). Impairment in the liver, heart or lungs was associated with further organ impairment in 63%, 62% and 48% f individuals respectively(**Figure 3**).

Symptoms and multi-organ impairment

For peer review only

Hepatic and pulmonary impairment frequently clustered together, with fatigue, muscle aches, fever and cough commonly reported. Impairment in particular organs was associated with particular symptoms: pancreas (diarrhoea, fever, headache and dyspnoea); heart (headache, dyspnoea and fatigue) and kidney (wheezing, runny nose, diarrhoea, cough, fever, headache, dyspnoea and fatigue) (**Figure 4**).

Hospitalisation, severity and multi-organ impairment

The hospitalised group were older ($p=0.001$), had higher BMI ($p=0.063$), were more likely to be non-white ($p=0.016$), and to report 'inability to walk' ($p=0.009$) than non-hospitalised individuals. There were no other statistically significant differences between risk factors or symptoms between the groups. Impairment of liver, pancreas (e.g. ectopic fat in the pancreas and liver, hepatomegaly) and ≥2 organs was higher in hospitalised individuals (all $p < 0.05$)(**Table 2, Figure 3)**. In multivariate analyses, adjusting for age, sex and BMI, liver volume remained significantly associated with hospitalisation ($p=0.001$). Hospitalised individuals had high triglycerides (30% vs 7.2%, $p=0.002$), cholesterol (60 vs 38%, $p=0.04$) and LDL-cholesterol (57 vs 31%, $p=0.01$), and low transferrin saturation (38 vs 15%, $p=0.01$), compared with non-hospitalised individuals. ESR (13%), bicarbonate (12%), uric acid (16%), platelet count (13%) and high-sensitivity CRP (15%) were high in ≥10% of hospitalised individuals.

60% (n=120) had severe PCS, with 52% reporting persistent, moderate problems undertaking usual activities (level 3 or greater in the relevant EQ-5D-5L question; 34% reported Dyspnoea-12 ≥10). Of those with severe PCS, 84% were not hospitalised, and 73% were female. There was no differences in age, BMI or ethnicity between the groups. Individuals with severe PCS

For peer review only - http://bmjopen.bmj.com/site/about/guidelines.xhtml

were more likely to report shortness of breath (p<.001), headache (p=0.019), chest pain (p=0.001), abdominal pain (p=0.001) and wheezing (p=0.039). 25% of those with 'severe' PCS had myocarditis compared to 12% with moderate PCS (unadjusted: 0.023; adjustment for age, sex and BMI: p=0.04, **Figure S2**). Severe PCS was associated with higher mean cell haemoglobin concentration (28% versus 17%), cholesterol (46.2 versus 32.8), CRP (10% versus 3.8%) and ESR (10% versus 6%), than moderate PCS, but these differences were not statistically significant (**Table S3**). Muscle aches, fever and coughing were common in severe PCS, and headache was common in individuals with pancreas inflammation (**Figure 4**).

Discussion

We report three findings in the first COVID-19 recovery study to evaluate medium-term, multi-organ impairment. First, in low-risk individuals, there were chronic symptoms and mild impairment in the heart, lung, liver, kidney and pancreas four months post-COVID-19, compared with healthy controls. Second, cardiac impairment was more common in severe PCS. Third, we demonstrate feasibility and potential utility of community-based multi-organ assessment for PCS.

Comparison with other studies

Common symptoms were fatigue, dyspnoea, myalgia, headache and arthralgia, despite low risk of COVID-19 mortality or hospitalisation. COVID-19 impact models have included age, underlying conditions and mortality, but not morbidity, multi-organ impairment and chronic diseases[29,30]. Even in non-hospitalised individuals, up to 10% of those infected have

For peer review only - http://bmjopen.bmj.com/site/about/guidelines.xhtml

PCS[15, 31], but studies of extra-pulmonary manifestations emphasise acute illness[32]. We describe mild rather than severe organ impairment, but the pandemic's scale and high infection rates in lower risk individuals signal medium- and longer- term COVID-19 impact, which cannot be ignored in healthcare or policy spheres.

Acute myocarditis and cardiogenic shock[33] are documented in hospitalised patients with COVID-19 [6]. In American athletes, recent COVID-19 was associated with myocarditis[34]. Although causality cannot be attributed, and post-viral syndromes have included similar findings[21], we show that one quarter of low-risk individuals with PCS have mild systolic dysfunction or myocarditis. The significance of these findings and associations with contemporaneous abnormal echocardiography findings and long-term myocardial fibrosis and impairment are unknown. Cardiac impairment, a risk factor for severe COVID-19, may have a role in PCS. Two further findings deserving investigation are pancreatic abnormalities, given the excess diabetes risk reported in PCS(15), and the preponderance of healthcare workers at increased PCS risk (as observed for COVID-19 mortality), possibly due to higher viral burden.

PCS is likely to be a syndrome rather than a single condition. Despite an immunologic basis for individual variations in COVID-19 progression and severity [35], prediction models have high rates of bias, perform poorly[36], and focus on respiratory dysfunction and decisions for ventilation in acutely unwell patients, rather than multi-organ function. Ongoing long-term studies[37] exclude non-hospitalised, low-risk individuals. During a pandemic, we studied subclinical organ impairment in PCS, showing low rates of incidental findings. As specialist PCS services are rolled out[38,39], multi-organ assessment, monitoring and community pathways have potential roles during and beyond COVID-19, but need to be evaluated.

For peer review only - http://bmjopen.bmj.com/site/about/guidelines.xhtml

Implications for research, clinical practice and public health

Our findings have three research implications. First, as countries face second waves, COVID-19 impact models should include PCS, whether quality of life, healthcare utilisation, or economic effects. Second, there is urgent need for multi-organ assessment, including blood and imaging, as well as primary and secondary care data linkage, to define PCS. Third, longitudinal studies of clustering of symptoms and organ impairment will inform health services research to plan multidisciplinary care pathways. There are three management implications. First, we signal the need for multi-organ monitoring in at least the medium-term, especially extra-pulmonary sequelae. Care pathways involving MRI (with limited access in many clinical settings) need evaluation versus other modalities to detect organ impairment (e.g. spirometry, NT-pro-BNP, ECG, echocardiography, ultrasound and blood investigations). Second, until effective vaccines and treatments are widely available, “infection suppression” (e.g. social distancing, masks, physical isolation) is the prevention strategy. Third, whether understanding baseline risk or multi-organ complications, PCS requires management across specialities (e.g. cardiology, gastroenterology) and disciplines (e.g. epidemiology, diagnostics, laboratory science)(**Figure 5**).

Limitations

There are some limitations. First, our cardiac MRI protocol excluded gadolinium contrast due to concerns regarding COVID-19-related renal complications, relying on native T1 mapping to characterise myocardial inflammation non-invasively (previously validated for acute myocarditis)[40]. Second, for organ impairment, we show association, not causation, and incidental findings are possible in asymptomatic individuals[41], but our findings are strengthened by comparison with healthy, age-matched controls, although not matched for sex or baseline comorbidities. Third, for pragmatic reasons, our controls were scanned using

For peer review only - http://bmjopen.bmj.com/site/about/guidelines.xhtml

1.5T, but we used 3T ranges as described in an analogous study with similar acquisition protocols. Therefore, we may be under-representing the true proportion of impairment in those individuals with PCS scanned at 3T. Fourth, further studies may explore different controls, e.g. individuals with post-flu symptoms, COVID-19 without symptoms or from general clinics. We will investigate duration, trajectory, complications and recovery for specific symptoms and organ impairment in the follow-up phase. Fifth, our study population was not ethnically diverse, despite disproportionate COVID-19 impact in non-white individuals. Sixth, to limit interaction and exposure between trial team and patients, pulse oximetry, spirometry, MRI assessment of brain and muscle function were not included from the outset.

Conclusions

Our study suggests PCS has a physiological basis, with measurable patient-reported outcomes and organ impairment. Future research should address longer-term follow-up of organ function beyond symptoms and blood investigations, even in lower risk individuals; prioritisation for imaging, investigation and referral; and optimal care pathways. Health system responses should emphasise infection suppression, and management of pre- and post-COVID-19 risk factors and chronic diseases.

Contributors:

Study design: AD, RB, JA, COVERSCAN team

Patient recruitment: RB, COVERSCAN team

Data collection: MW, COVERSCAN team

Data analysis: AD, COVERSCAN team, AB

Data interpretation: AB, AD, MW, RB

For peer review only

For peer review only - http://bmjopen.bmj.com/site/about/guidelines.xhtml

Initial manuscript drafting: AB, AD, RB

Critical review of early and final versions of manuscript: all authors including JO and DC.

Specialist input: cardiology (MW, AB); general medicine (RB, MH, DW, MC, DC); long COVID (MH, MC, DW); imaging (MB, RB); statistics (AD); epidemiology/public health (AB); primary care (MG); healthcare management (JA); patient and public involvement (LH, EA).

COVERSCAN study investigators

Perspectum: Mary Xu, Faezah Sanaei-Nezhad, Andrew Parks, Andrea Borghetto, Matthew D Robson, Petrus Jacobs, John Michael Brady, Carla Cascone, Soubera Rymell, Jacky Law, Virginia Woolgar, Velko Tonev, Claire Herlihy, Rob Suriano, Tom Waddell, Henrike Puchta, Alessandra Borlotti, Arun Jandor, Freddie Greatrex, Robin Jones, Georgina Pitts, Ashleigh West, Marion Maguire, Anu Chandra, Naomi Jayaratne, Dali Wu, Stella Kin, Mike Linsley, Valentina Carapella, Isobel Gordon, George Ralli, John McGonigle, Darryl McClymont, Boyan Ivanov, James Owler, Diogo Cunha, Tatiana Lim, Carlos Duncker, Madison Wagner, Marc Goldfinger, Adriana Roca, Charlotte Erpicum, Matthew David Kelly, Rexford D Newbould, Catherine J Kelly, Andrea Dennis, Sofia Mouchti, Arina Kazimianec, Helena Thomaides-Briers, Rajarshi Banerjee

University College London: Amitava Banerjee

Great Western Hospitals NHS Foundation Trust: Malgorzata Wamil

University of Oxford: Yi-Chun Wang, Tom Waddell

Mayo Clinic: Sandeep Kapur and Louise McLaughlin

Funding:

This work was supported by the UK's National Consortium of Intelligent Medical Imaging (Industry Strategy Challenge Fund), Innovate UK (Grant 104688) and the European Union's Horizon 2020 research and innovation programme (agreement No 719445). The research was

For peer review only

designed, conducted, analysed, and interpreted by the authors independently of the funding sources.

Competing interests: All authors have completed the ICMJE uniform disclosure form at www.icmje.org/coi_disclosure.pdf. AD, RB and MB are employees of Perspectum. All other authors declare no financial relationships with any organisations that might have an interest in the submitted work in the previous three years; no other relationships or activities that could appear to have influenced the submitted work.

Ethical approval

The study (https://clinicaltrials.gov/ct2/show/NCT04369807) had ethical approval (20/SC/0185).

Data sharing statement

Deidentified participant data are available from the corresponding authors on request.

Transparency statement

AB affirms that this manuscript is an honest, accurate, and transparent account of the reported study; that no important study aspects have been omitted; and that any discrepancies from the study as planned (and, if relevant, registered) have been explained.

Dissemination to participants and related patient and public communities: The results of this study will be disseminated to relevant patient organisations (e.g. Long COVID SOS), policymakers and health professionals.

Provenance and peer review: Not commissioned; externally peer reviewed.

For peer review only

Figures and Table Titles and Legends

Table 1: Baseline demographics and symptoms in 201 low-risk individuals with post COVID syndrome.

**Data are presented as count (%). Comparisons between managed at home vs hospitalised, and between moderate vs post-COVID syndrome were conducted using Fisher's exact test.*

Table 2: Evidence of organ impairment in 201 low-risk individuals with post COVID syndrome.

**Data are presented as count (%). Comparisons between managed at home vs hospitalised, and between moderate vs post-COVID syndrome were conducted using Fisher's exact test.*

For peer review only

Figure 1: Flow from recruitment to enrolment of 201 patients with post COVID syndrome.

Figure 2. Percentage of patients (black) and controls (grey) with individual organ measures outside of the pre-defined normal range. Lines represent significant difference in the proportions between the two groups and significant stars represent *p<0.05; **p<0.01; *** p<.001.

Figure 3: Multi-organ impairment in low-risk individuals with post COVID syndrome by gender and hospitalisation.

Figure 4: Percentage of reported symptoms during the acute phases of the illness within those with evidence of organ impairment for each organ separately. Darker red indicates higher percentage of reported symptoms per impaired organ, there are no distinct patterns of symptoms relating to each impaired organ but highlights a high burden of symptoms individual

Figure 5: Natural history of post COVID syndrome, the COVERSCAN study in low-risk individuals (n=201) and policy recommendations.

For peer review only

For peer review only - http://bmjopen.bmj.com/site/about/guidelines.xhtml

Table 3: Baseline demographics and symptoms in 201 low-risk individuals with post COVID syndrome.

	All Patients (n=201)	Healthy Controls (N=36)	P	Not hospitalised (n=163)	Hospitalised (n=37)	P	Moderate PCS (n=77)	Severe PCS (n=116)	p
**Age** (yrs, mean; sd)	44 (11.0)	39 (12.4)	0.013	43 (10.9)	50 (10.0)	0.001	45 (12.2)	44 (10.0)	0.419
**Female** (No, %)	142 (70.6)	14 (38.9)	0.032	118 (72.4)	23 (62.2)	0.302	51 (66.2)	85 (73.3)	0.374
**BMI** (kg.m^{-2}); median(IQR)	25.7(22.7-28.1)	23.2 (21.4-23.1)	<0.001	25.3 (22.7-27.7)	27.2 (23.1-31.0)	0.063	25.8 (22.7-27.9)	25.4 (22.5-28.2)	0.639
Ethnicity									
White	176 (87.6)	33 (91.7)	0.904	148 (90.8)	28 (75.7)	0.016	67 (87.0)	106 (91.4)	0.178
Mixed	3 (1.5)	0 (0)		3 (1.8)	0 (0)		1 (1.3)	2 (1.7)	
South Asian	7 (3.5)	3 (8.3)		4 (2.5)	3 (8.1)		5 (6.5)	0 (0)	
Black	4 (2.0)	0 (0)		1 (0.6)	2 (5.4)		2 (2.6)	2 (1.7)	
Comorbidities and risks									
Smoking									
Never	133 (66.2)	20 (60.6)		108 (66.3)	24 (64.9)		55 (71.4)	72 (61.7)	
Current	6 (3.0)	8 (24.2)	<0.001	6 (3.7)	0 (0)	0.641	3 (3.9)	3 (2.6)	0.244
Ex-smoker	62 (30.8)	5 (15.2)		49 (30.1)	13 (35.1)		19 (24.7)	41 (35.3)	
**Health care worker**	64 (31.8)	4 (12.1)	0.009	50 (30.7)	13 (35.1)	0.695	33 (42.9)	28 (24.1)	0.007
**Asthma**	37 (18.4)	0 (0)	0.002	34 (20.9)	3 (8.1)	0.099	13 (16.9)	22 (19.0)	0.849
**BMI**									
≥25 kg/m^2	113 (56.5)	7 (20)		87 (53.7)	25 (67.6)	0.144	46 (60.5)	62 (53.4)	0.374
≥30 kg/m^2	40 (20.0)	0 (0)		28 (17.3)	12 (32.4)	0.066	16 (21.1)	24 (20.7)	1.000
**Hypertension**	13 (6.5)	0 (0)	0.001	11 (6.7)	2 (5.4)	1.000	6 (7.8)	7 (6.0)	0.771
**Diabetes**	4 (2.0)	0 (0)	0.104	4 (2.5)	0 (0.0)	1.000	4 (5.2)	0 (0.0)	0.024
**Previous heart disease**	9 (4.5)	0 (0)	0.001	8 (4.9)	1 (2.7)	1.000	3 (3.9)	5 (4.3)	1.000
Symptoms									

For peer review only

For peer review only - http://bmjopen.bmj.com/site/about/guidelines.xhtml

Fatigue	196 (98.0)			159 (97.5)	37 (100.0)	1.000	73 (96.1)	115 (99.1)	0.302
Shortness of breath	176 (88.0)			141 (86.5)	35 (94.6)	0.262	58 (76.3)	112 (96.6)	<0.0001
Muscle ache	173 (86.5)			142 (87.1)	31 (83.8)	0.597	66 (86.8)	101 (87.1)	1.000
Headache	165 (82.5)			138 (84.7)	27 (73.0)	0.098	56 (73.7)	102 (87.9)	0.019
Joint pain	156 (78.0)			127 (77.9)	29 (78.4)	1.000	56 (73.7)	94 (81.0)	0.284
Chest pain	152 (76.0)			128 (78.5)	24 (64.9)	0.090	47 (61.8)	98 (84.5)	0.001
Cough	146 (73.0)			117 (71.8)	29 (78.4)	0.539	55 (72.4)	84 (72.4)	1.000
Fever	144 (72.0)			113 (69.3)	31 (83.8)	0.104	51 (67.1)	86 (74.1)	0.329
Sore throat	143 (71.5)			120 (73.6)	23 (62.2)	0.165	50 (65.8)	86 (74.1)	0.256
Diarrhoea	118 (59.0)			91 (55.8)	27 (73.0)	0.065	40 (52.6)	76 (65.5)	0.097
Abnormal pain	108 (54.0)			91 (55.8)	17 (45.9)	0.361	30 (39.5)	75 (64.7)	0.001
Wheezing	98 (49.0)			75 (46.0)	23 (62.2)	0.101	30 (39.5)	64 (55.2)	0.039
Inability to walk	80 (40.0)			58 (35.6)	22 (59.5)	0.009	24 (31.6)	50 (43.1)	0.130
Runny nose	68 (34.0)			55 (33.7)	13 (35.1)	0.85	24 (31.6)	41 (35.3)	0.642
Time interval									
Initial symptoms-to-assessment (days): median (IQR)	141 (110, 162)			141 (112-163)	138 (97-150)	0.106	121 (89-158)	145 (121-163)	0.001
COVID-19 positive-to-assessment (days): median, (IQR)	71 (41, 114			68 (35-112)	105 (59-126)	0.012	60 (43,98)	78 (34-119)	0.305

Table 4: Evidence of organ impairment in 201 low-risk individuals with post COVID syndrome.

Measurement	All Patients (n=201)	Healthy Controls (n=36)	P	Not hospitalised (n=163)	Hospitalised (n=37)	P	Moderate PCS (n=77)	Severe PCS (n=116)	P
HEART									
Left ventricular ejection fraction (%)									
• Normal (>51%)	190 (95.0)	35 (97.2)	0.699	155 (95.7)	33 (89.1)	0.124	72 (93.5)	111 (95.7)	0.353
• Impaired (≤51%)	11 (5.0)	1 (2.8)		7 (4.3)	4 (10.1)		5 (6.4)	5 (4.3)	
Left ventricular end diastolic volume (ml)									
• >264ml in M; >206ml in W	8 (4.0)	1 (2.8)	1.00	4 (2.5)	4 (10.8)	0.040	4 (5.2)	4 (3.4)	0.715
Evidence of myocarditis									
• ≥ 3 segments with high T1 (≥1229ms at 3T; ≥1015ms at 1.5T)	39 (19.4)	2 (5.6)	0.053	30 (18.4)	8 (21.6)	0.647	9 (11.7)	29 (25.0)	0.027

For peer review only - http://bmjopen.bmj.com/site/about/guidelines.xhtml

LUNGS									
Deep Breathing Fractional area change	(n=17 missing)			(n=13 missing)	(n=3 missing)		(n=8 missing)	(n=7 missing)	
• < 31%	21 (11.4)	1 (2.8)	0.138	17 (11.3)	4 (11.8)	1	7 (10.1)	13 (11.9)	0.811
KIDNEYS									
Kidney cortex T1	(n=3 missing)			(n=3 missing)			(n=2 missing)		
• Normal (<1652 ms at 3T; <1227ms at 1.5T)	191 (96.5)	36 (100.0)	0.599	155 (96.9)	35 (94.6)	0.618	74 (98.7)	112 (96.6)	0.65
• Impaired (≥1652 ms at 3T; ≥1227ms at 1.5T)	7 (3.5)	0 (0.0)		5 (3.1)	2 (5.4)		1 (1.3)	4 (3.4)	
PANCREAS									
Pancreatic inflammation (T1 in ms)	(n=11 missing)	(n=13 missing)		(n=7 missing)	(n=4 missing)		(n=4 missing)	(n=6 missing)	
• Normal <803ms	162 (85.3)	23 (100.0)	0.049	139 (89.1)	22 (66.7)	0.002	60 (82.2)	95 (86.4)	0.530
• Impaired ≥803ms	28 (14.7)	0 (0)		17 (10.9)	11 (33.3)		13 (17.8)	15 (13.6)	
Pancreatic fat		(n=4 missing)							
• Normal <4.6%	122 (62.2)	30 (93.8)	<0.001	107 (66.9)	14 (40.0)	0.004	44 (57.9)	72 (63.7)	0.449
• Impaired ≥4.6%	74 (37.8)	2 (6.2)		53 (33.1)	21 (60.0)		32 (42.1)	41 (36.3)	
LIVER									
Liver Inflammation (cT1 in ms)	(n=1 missing)			(n=1 missing)			(n=1 missing)		
• Normal <784ms	177 (88.5)	36 (100)	0.030	148 (91.4)	28 (75.7)	0.018	69 (90.8)	101 (87.1)	0.494
• Impaired ≥784ms	23 (11.5)	0 (0)		14 (8.6)	9 (24.3)		7 (9.2)	15 (12.9)	
Liver fat									
• Normal <4.8%	159 (79.1)	34 (94.4)	0.034	134 (82.2)	24 (64.9)	0.026	61 (79.2)	91 (78.4)	1
• Impaired ≥4.8%	42 (20.9)	2 (5.4)		29 (17.8)	13 (35.1)		16 (20.8)	25 (21.6)	
Liver volume		(n=1 missing)							
• Normal <1935ml	180 (89.6)	34 (97.1)	0.214	154 (94.5)	25 (67.6)	<0.0001	68 (88.3)	104 (89.7)	0.816
• Impaired ≥1935ml	21 (10.4)	1 (2.9)		9 (5.5)	12 (32.4)		9 (11.7)	12 (10.3)	
SPLEEN									
Splenic volume (ml)		(n=1 missing)							
• Normal <350ml	194 (96.5)	32 (91.4)	0.172	160 (98.2)	33 (89.2)	0.023	74 (96.1)	112 (96.6)	1
• Impaired ≥350ml	7 (3.5)	3 (8.6)		3 (1.8)	4 (10.8)		3 (3.9)	4 (3.4)	

For peer review only

Data are presented as count (%). Comparisons between managed at home vs hospitalised, and between moderate vs post-COVID syndrome were conducted using Fisher's exact test.

For peer review only

For peer review only - http://bmjopen.bmj.com/site/about/guidelines.xhtml

References

1. World Health Organization. Clinical management of severe acute respiratory infection (SARI) when COVID-19 disease is suspected. Interim guidance 13 March 2020. https://apps.who.int/iris/handle/10665/331446
2. Pavon AG, Meier D, Samim D, Rotzinger DC, Fournier S, Marquis P, et al. First Documentation of Persistent SARS-Cov-2 Infection Presenting With Late Acute Severe Myocarditis. Can J Cardiol. 2020;36(8):1326.e5-1326.e7.
3. Puntmann VO, Carerj ML, Wieters I, Fahim M, Arendt C, Hoffmann J, et al. Outcomes of Cardiovascular Magnetic Resonance Imaging in Patients Recently Recovered from Coronavirus Disease 2019 (COVID-19). JAMA Cardiol. 2020;2019(11):1265–73.
4. Tabary M, Khanmohammadi S. Pathologic features of COVID-19: A concise review. Pathol - Res Pract. 2020;216(9): 153097.
5. Alqahtani SA, Schattenberg JM. Liver injury in COVID-19: The current evidence. United Eur Gastroenterol J. 2020;8(5):509–19.
6. Somasundaram NP, Ranathunga I, Ratnasamy V, Wijewickrama PSA, Dissanayake HA, Yogendranathan N, et al. The impact of SARS-Cov-2 virus infection on the endocrine system. J Endocr Soc. 2020;4(8):1–22.
7. Farouk SS, Fiaccadori E, Cravedi P, Campbell KN. COVID-19 and the kidney: what we think we know so far and what we don't. J Nephrol. 2020. Jul 20 : 1–6.
8. Lai A, Pasea L, Banerjee A, Denaxas S, Katsoulis M, Chang W, et al. Estimating excess mortality in people with cancer and multimorbidity in the COVID-19 emergency. BMJ Open. 2020. Nov 17;10(11):e043828.
9. Banerjee A, Pasea L, Harris S, Gonzalez-Izquierdo A, Torralbo A, Shallcross L, et al. Estimating excess 1-year mortality associated with the COVID-19 pandemic according to underlying conditions and age: a population-based cohort study. Lancet. 2020;395(10238):1715–25.
10. Banerjee A, Chen S, Pasea L, Lai A, Katsoulis M, Denaxas S, et al. Excess deaths in people with cardiovascular diseases during the COVID-19 pandemic. Eur J Prev Cardiol. 2020. In press.
11. Raman B, Philip Cassar M, Tunnicliffe EM, Filippini N, Griffanti L, Alfaro-Almagro F, et al. Medium-term effects of SARS-CoV-2 infection on multiple vital organs, exercise capacity, cognition, quality of life and mental health, post-hospital discharge. EClinicalMedicine. 2021 Jan 7;31:100683.
12. Horton R. Offline: COVID-19 is not a pandemic. Lancet. 2020;396(10255):874.
13. Shovlin CL, Vizcaychipi MP. Implications for COVID-19 triage from the ICNARC report of 2204 COVID-19 cases managed in UK adult intensive care units. Emerg Med J.

For peer review only

2020;37(6):332–3.

14. Docherty AB, Harrison EM, Green CA, Hardwick HE, Pius R, Norman L, et al. Features of 20 133 UK patients in hospital with covid-19 using the ISARIC WHO Clinical Characterisation Protocol: Prospective observational cohort study. BMJ. 2020;369:1–12.
15. Williamson E, Walker A, Bhaskaran K, Bacon S, Curtis H, Mehrkar A, et al. Factors associated with COVID-19-related death using OpenSAFELY. Nature. 2020; 584(7821):430-436.
16. Office for National Statistics. The prevalence of long COVID symptoms and COVID-19 complications. 16 December 2020. https://www.ons.gov.uk/news/statementsandletters/theprevalenceoflongcovidsymptomsandcovid19complications
17. Del Rio C, Collins L, Malani P. Long-term Health Consequences of COVID-19 Carlos. JAMA. 2020;324(17).
18. Carfi A, Bernabei R, Landi F. Persistent Symptoms in Patients After Acute COVID-19. JAMA. 2020;324(6):604–5.
19. Nabavi N. Long covid: How to define it and how to manage it. BMJ. 2020;370:m3489.
20. Greenhalgh T, Knight M, A'Court C, Buxton M, Husain L. Management of post-acute covid-19 in primary care. BMJ. 2020;370.
21. National Institute for Health Research: New risk prediction model could help improve guidance for people shielding from COVID-19. June 2020. https://www.nihr.ac.uk/news/new-risk-prediction-model-could-help-improve-guidance-for-people-shielding-from-covid-19/25096
22. Hill AD, Fowler RA, Pinto R, Herridge MS, Cuthbertson BH, Scales DC. Long-term outcomes and healthcare utilization following critical illness - a population-based study. Crit Care. 2016;20(1):1–10.
23. Perrin R. Into the looking glass: Post-viral syndrome post COVID-19. Med Hypotheses. 2020;144.
24. George PM, Barratt SL, Condliffe R, Desai SR, Devaraj A, Forrest I, et al. Respiratory follow-up of patients with COVID-19 pneumonia. Thorax. 2020;75(11):1009–16.
25. Jiang DH, McCoy RG. Planning for the Post-COVID Syndrome: How Payers Can Mitigate Long-Term Complications of the Pandemic. J Gen Intern Med. 2020;35(10):3036–9.
26. Janssen MF, Pickard AS, Golicki D, Gudex C, Niewada M, Scalone L, et al. Measurement properties of the EQ-5D-5L compared to the EQ-5D-3L across eight patient groups: A multi-country study. Qual Life Res. 2013;22(7):1717–27.

For peer review only - http://bmjopen.bmj.com/site/about/guidelines.xhtml

27. Yorke J, Moosavi SH, Shuldham C, Jones PW. Quantification of dyspnoea using descriptors: development and initial testing of the Dyspnoea-12. Thorax. 2010;65(1):21–6.
28. Hobbins A, Barry L, Kelleher D, O'Neill C. The health of the residents of Ireland: Population norms for Ireland based on the EQ-5D-5L descriptive system – a cross sectional study. HRB Open Res. 2018;1:22.
29. Gupta A, Madhavan M V., Sehgal K, Nair N, Mahajan S, Sehrawat TS, et al. Extrapulmonary manifestations of COVID-19. Nat Med. 2020;26(7):1017–32.
30. Palmer K, Monaco A, Kivipelto M, Onder G, Maggi S, Michel JP, et al. The potential long-term impact of the COVID-19 outbreak on patients with non-communicable diseases in Europe: consequences for healthy ageing. Aging Clin Exp Res [Internet]. 2020;32(7):1189–94.
31. Menni C, Valdes AM, Freidin MB, Sudre CH, Nguyen LH, Drew DA, et al. Real-time tracking of self-reported symptoms to predict potential COVID-19. Nat Med. 2020;26(7):1037–40.
32. Mandal S, Barnett J, Brill S, Brown J, Denneny E, Hare S, et al. "Long-COVID": a cross-sectional study of persisting symptoms, biomarker and imaging abnormalities following hospitalisation for COVID-19. Thorax. 2020; Nov 10;thoraxjnl-2020-215818.
33. Chau VQ, Giustino G, Mahmood K, Oliveros E, Neibart E, Oloomi M, et al. Cardiogenic shock and hyperinflammatory syndrome in young males with COVID-19. Circ Hear Fail. 2020;556–9.
34. Rajpal S, Tong MS, Borchers J, Zareba KM, Obarski TP, Simonetti OP, et al. Cardiovascular Magnetic Resonance Findings in Competitive Athletes Recovering from COVID-19 Infection. JAMA Cardiol. 2020;5–7.
35. Mathew D, Giles JR, Baxter AE, Oldridge DA, Greenplate AR, Wu JE, et al. Deep immune profiling of COVID-19 patients reveals distinct immunotypes with therapeutic implications. Science. 2020; 369(6508):eabc8511.
36. Wynants L, Van Calster B, Collins GS, Riley RD, Heinze G, Schuit E, et al. Prediction models for diagnosis and prognosis of covid-19: Systematic review and critical appraisal. BMJ. 2020;369 :m1328.
37. PHOSP-COVID: Post-HOSPitalisation COVID-19 study. https://www.phosp.org/
38. NHS to offer 'long covid' sufferers help at specialist centres. https://www.england.nhs.uk/2020/10/nhs-to-offer-long-covid-help/
39. National Institute for Healh and Care Excellence. COVID-19 rapid guideline: managing the long-term effects of COVID-19. 18 December 2020. https://www.nice.org.uk/guidance/ng188

For peer review only

40. Ferreira VM, Piechnik SK, Armellina ED, Karamitsos TD, Francis JM, Ntusi N, et al. Native T1-mapping detects the location , extent and patterns of acute myocarditis without the need for gadolinium contrast agents. 2014;16(1):1–11.

41. Gibson LM, Paul L, Chappell FM, Macleod M, Whiteley WN, Salman RAS, et al. Potentially serious incidental findings on brain and body magnetic resonance imaging of apparently asymptomatic adults: Systematic review and meta-analysis. BMJ. 2018;363: k4577.

For peer review only

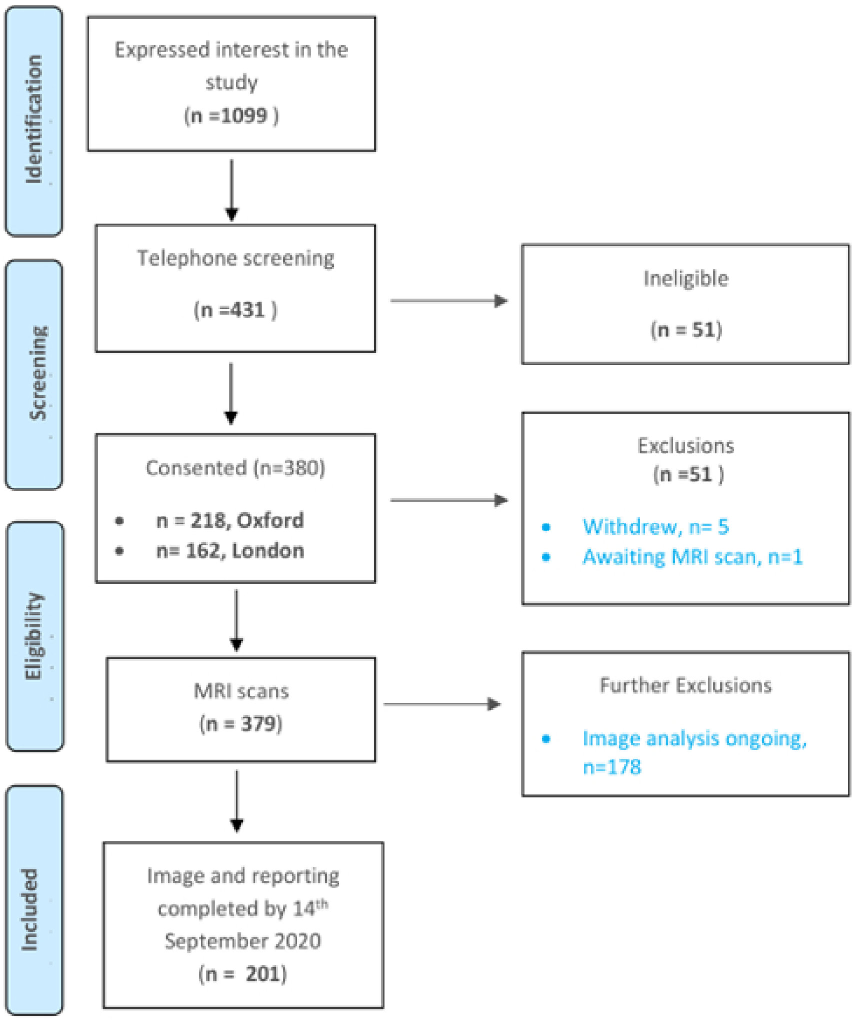

Figure 1. Flow from recruitment to enrolment of 201 patients with post COVID syndrome.

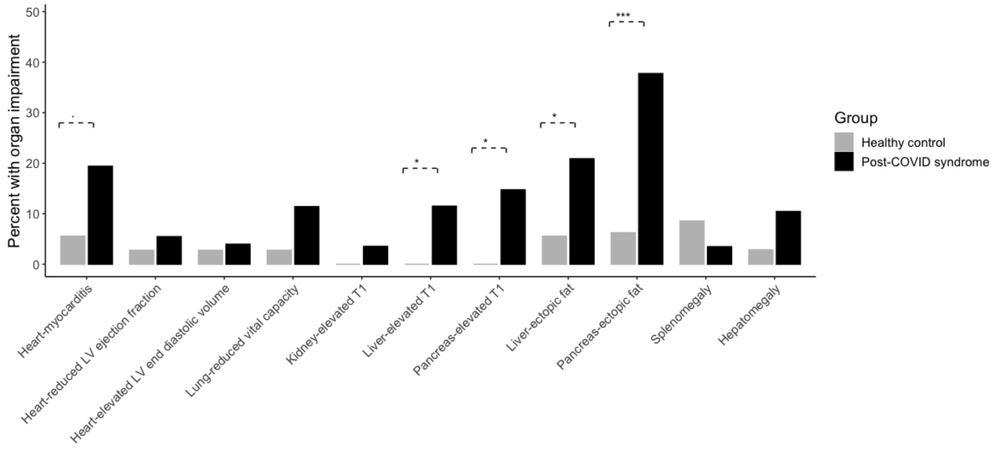

Figure 2. Organ impairment in low-risk individuals with post COVID syndrome (n=201) compared to healthy controls (n=36).

Significance: . p=0.05; *p<0.05; **p<0.01; *** p<.001.

418x206mm (72 x 72 DPI)

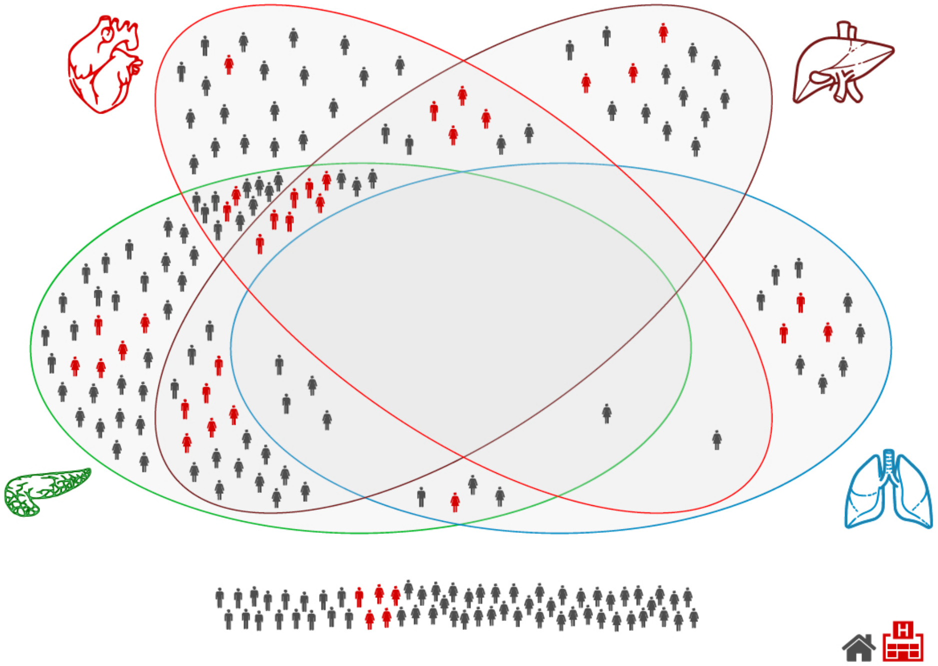

Figure 3. Multi-organ impairment in low-risk individuals with post COVID syndrome by gender and hospitalisation.

198x141mm (144 x 144 DPI)

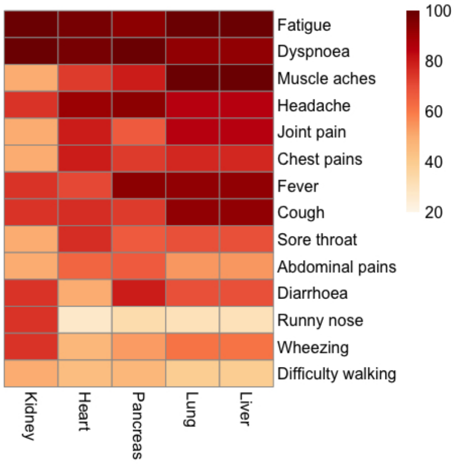

Figure 4: Reported symptoms and organ impairment in individuals with severe post COVID syndrome.

Darker red indicates higher percentage of reported symptoms per impaired organ.

349x206mm (72 x 72 DPI)

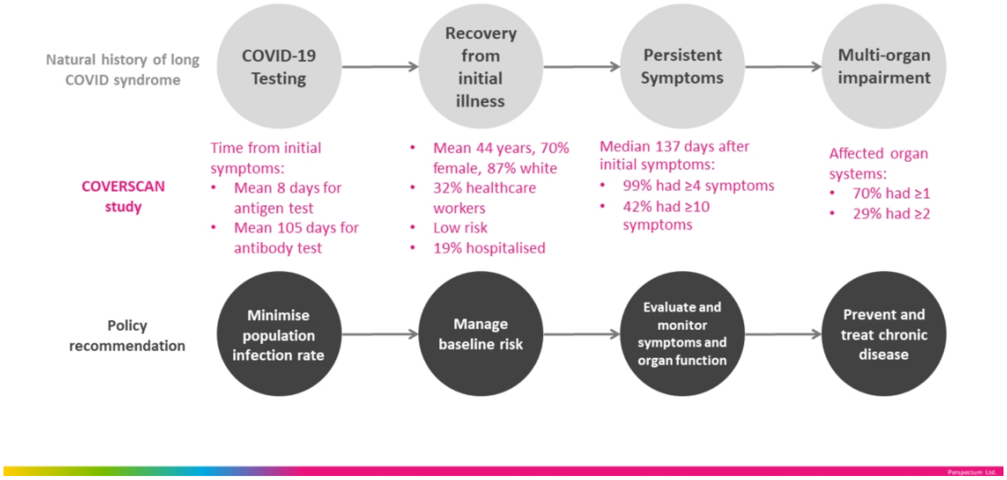

Figure 5: Natural history of post COVID syndrome, the COVERSCAN study in low-risk individuals (n=201) and policy recommendations.

338x190mm (96 x 96 DPI)

Web Supplementary Materials

Supplementary methods 2

Supplementary references 4

Supplementary results 6

Figure S1: Comparison of patients to control quantitative image derived measures in a subset of those scanned at 1.5T 6

Figure S2: Organ impairment in severe versus moderate post COVID syndrome (n=201) 7

Table S1: Reference ranges to define organ impairment 7

Table S2: Blood investigations in 201 low-risk individuals with post-COVID syndrome, sub-divided by hospitalisation or managed at home 8

Table S3: Blood investigations in 201 low-risk individuals, sub-divided by those with severe of moderate post-COVID syndrome 13

For peer review only

For peer review only - http://bmjopen.bmj.com/site/about/guidelines.xhtml

Supplementary methods

Blood investigations

Blood investigations included: full blood count, serum biochemistry (sodium, chloride, bicarbonate, urea, creatinine, bilirubin, alkaline phosphatase, aspartate transferase, alanine transferase, lactate dehydrogenase, creatinine kinase, gamma-glutamyl transpeptidase, total protein, albumin, globulin, calcium, magnesium, phosphate, uric acid, fasting triglycerides, cholesterol (total, HDL, LDL), iron, iron-binding capacity (unsaturated and total) and inflammatory markers (erythrocyte sedimentation rate, ESR; high sensitivity-C-Reactive Protein, CRP) (TDL laboratories, London).

Imaging

All the imaging methods can be deployed on standard clinical MRI scanners and are generally expedited approaches of methods previously demonstrated in the scientific literature that unless stated each utilise a short (<14seconds) breath-hold.

Cardiac imaging involved complete coverage of the heart with a short-axis stack (to the valve plane) of cine images acquired using cardiac gating, this acquisition mirrors that in UK Biobank and is a standardized approach(S1). Three short-axis cardiac T1 maps are acquired using the MOLLI-T1 approach at the basal, mid and apical levels of the left ventricle.

Liver and pancreas imaging used the LiverMultiScan acquisition protocol (Perspectum, Oxford, UK), which involves 3 single 2D axial slice breath-held acquisitions that separately are sensitive to the fat content (proton density fat fraction, or PDFF), to T2* (which is representative of liver iron content) and a MOLLI-T1 measurement (providing a measurement of tissue water), additionally a volumetric scan was used that covers the entire liver(S2).

Two dynamic cine MR acquisitions of the lung were acquired in the coronal plane with a 306.91 ms temporal resolution: one 40 s acquisition with the patient instructed to breathe normally and a second 30 s acquisition with the patient instructed to breathe deeply.

Kidney imaging used a single coronal view that was able to image both kidneys, imaging contrasts were MOLLI-T1, T2* (for blood oxygen level assessment), and diffusion imaging that was acquired during free-breathing in 2minutes.

Image Analysis

Cardiac MRI Analysis: Experienced cardiac MRI analysts used CVI42 (Cardiovascular Imaging Inc, Canada) to manually trace the end-diastolic and end-systolic phases in each of the short-axis views, following the standard UK BioBank evaluation approach as previously described(S3). This analysis yielded: For both the left and the right ventricle; End diastolic volume, End systolic volume, Stroke volume and Ejection Fraction. Additionally left ventricular muscle mass and wall thickness are determined from the function data. Cardiac T1 was determined for each of the 16 cardiac segments (of the AHA 17 segment model)(S4).

Liver Images were analysed by data analysts experienced at using the LiverMultiScan (Perspectum, Oxford, UK) software. This yielded global metrics in each liver of PDFF (proton density fat fraction), T2*, and cT1 (cT1 is a measurement of T1 that has been corrected for the confounding effects of iron and standardised to 3 Tesla; it is elevated with disease).

Pancreas images were analysed in a similar manner to the above except the software used was not FDA-cleared and iron correction was not performed. The output T1 was standardized to 3 Tesla.

For peer review only - http://bmjopen.bmj.com/site/about/guidelines.xhtml

Lung cine imaging allowed the measurement of the area of the left and right lungs through the breathing cycle in the coronal plane, which used automated methods that were reviewed by image analysts. The periodicity of the area fluctuations was used to determine the respiratory rate. All analysis was performed in-house using MATLAB based tools. The method was validated by measuring the correlation between the change in area and the forced vital capacity, the latter being measured using spirometry.

Patient respiration was assessed by imaging a single 2D coronal slice of the lungs over 30 seconds using a dynamic cine MRI acquisition, during which the patient instructed to breathe deeply.

Kidney images were assessed using in-house tools to fit the parametric maps and allow trained analysts to make measurements. The T2* maps were analysed by the Twelve Layer Concentric Object (TLCO) approach that generates a gradient of relaxation values, in the other evaluations the cortex and medulla were manually segmented using the MOLLI-T1 map or the b=0 (in the case of diffusion) to guide the boundary.

In all cases the volumetric assessments utilised an initial in-house developed machine-learning driven segmentation, and then a manual step that may be used to fine tune boundaries. This approach was also used in the body composition analysis, which for reasons of speed was performed only in a single slice (an axial view that passes through L3 of the spine) in this work.

For peer review only

For peer review only - http://bmjopen.bmj.com/site/about/guidelines.xhtml

Supplementary references

S1. Petersen SE, Matthews PM, Francis JM, Robson MD, Zemrak F, Boubertakh R, Young AA, Hudson S, Weale P, Garratt S, Collins R, Piechnik S, Neubauer S. UK Biobank's cardiovascular magnetic resonance protocol. J Cardiovasc Magn Reson. 2016 Feb 1;18:8.

S2. Banerjee R, Pavlides M, Tunnicliffe EM, Piechnik SK, Sarania N, Philips R, Collier JD, Booth JC, Schneider JE, Wang LM, Delaney DW, Fleming KA, Robson MD, Barnes E, Neubauer S. Multiparametric magnetic resonance for the non-invasive diagnosis of liver disease. J Hepatol. 2014 Jan;60(1):69-77. doi: 10.1016/j.jhep.2013.09.002.

S3. Petersen SE, Aung N, Sanghvi MM, Zemrak F, Fung K, Paiva JM, Francis JM, Khanji MY, Lukaschuk E, Lee AM, Carapella V, Kim YJ, Leeson P, Piechnik SK, Neubauer S. Reference ranges for cardiac structure and function using cardiovascular magnetic resonance (CMR) in Caucasians from the UK Biobank population cohort. J Cardiovasc Magn Reson. 2017 Feb 3;19(1):18.

S4. Cerqueira MD, Weissman NJ, Dilsizian V, Jacobs AK, Kaul S, Laskey WK, Pennell DJ, Rumberger JA, Ryan T, Verani MS; American Heart Association Writing Group on Myocardial Segmentation and Registration for Cardiac Imaging. Standardized myocardial segmentation and nomenclature for tomographic imaging of the heart. A statement for healthcare professionals from the Cardiac Imaging Committee of the Council on Clinical Cardiology of the American Heart Association. Circulation. 2002 Jan 29;105(4):539-42.

S5. Kawel-Boehm N, Maceira A, Valsangiacomo-Buechel ER, Vogel-Claussen J, Turkbey EB, Williams R, Plein S, Tee M, Eng J, Bluemke DA. Normal values for cardiovascular magnetic resonance in adults and children. J Cardiovasc Magn Reson. 2015 Apr 18;17(1):29.).

S6. Ponikowski P, Voors AA, Anker SD, Bueno H, Cleland JGF, Coats AJS, Falk V, González-Juanatey JR, Harjola VP, Jankowska EA, Jessup M, Linde C, Nihoyannopoulos P, Parissis JT, Pieske B, Riley JP, Rosano GMC, Ruilope LM, Ruschitzka F, Rutten FH, van der Meer P; ESC Scientific Document Group. 2016 ESC Guidelines for the diagnosis and treatment of acute and chronic heart failure: The Task Force for the diagnosis and treatment of acute and chronic heart failure of the European Society of Cardiology (ESC)Developed with the special contribution of the Heart Failure Association (HFA) of the ESC. Eur Heart J. 2016 Jul 14;37(27):2129-2200.

S7. Tsao CW, Lyass A, Larson MG, Cheng S, Lam CS, Aragam JR, Benjamin EJ, Vasan RS. Prognosis of adults with borderline left ventricular ejection fraction. JACC Heart Fail. 2016 Jun;4(6):502-10.

S8. Chalasani, Naga, et al. The diagnosis and management of nonalcoholic fatty liver disease: practice guidance from the American Association for the Study of Liver Diseases. Hepatology 67.1 (2018): 328-357

S9. Mojtahed A, Kelly C, Herlihy A, et al. Reference range of liver corrected T1 values in a population at low risk for fatty liver disease-a UK Biobank sub-study, with an appendix of interesting cases. Abdomimal Radiol 2019; 44: 72–84.

S10. Jayaswal AN, Levick C, Selvaraj EA, et al. Prognostic value of multiparametric MRI, transient elastography and blood-based fibrosis markers in patients with chronic liver disease. Liver Int 2020; in press. DOI:doi:10.1111/liv.14625.

S11. Jayaswal ANA, Levick C, Selvaraj EA, Dennis A, Booth JC, Collier J, Cobbold J, Tunnicliffe EM, Kelly M, Barnes E, Neubauer S, Banerjee R, Pavlides M. Prognostic value of multiparametric magnetic

For peer review only - http://bmjopen.bmj.com/site/about/guidelines.xhtml

resonance imaging, transient elastography and blood-based fibrosis markers in patients with chronic liver disease. Liver Int. 2020 Jul 30. doi: 10.1111/liv.14625

S12. Chouhan MD, Firmin L, Read S, Amin Z, Taylor SA. Quantitative pancreatic MRI: a pathology-based review. Br J Radiol. 2019 Jul;92(1099):20180941.

S13. Harrington KA, Shukla-Dave A, Paudyal R, Do RKG. MRI of the Pancreas. J Magn Reson Imaging. 2020 Apr 17. doi: 10.1002/jmri.27148.

S14. Gillis KA, McComb C, Patel RK, et al. Non-contrast renal magnetic resonance imaging to assess perfusion and corticomedullary differentiation in health and chronic kidney disease. Nephron 2016; 133: 183–92.

S15. Peperhove M, Vo Chieu VD, Jang M-S, et al. Assessment of acute kidney injury with T1 mapping MRI following solid organ transplantation. Eur Radiol 2018; 28: 44–50.

S16. Chow KU, Luxembourg B, Seifried E, Bonig H. Spleen size is significantly influenced by body height and sex: establishment of normal values for spleen size at us with a cohort of 1200 healthy individuals. Radiology 2015; 279: 306–13.

For peer review only

For peer review only - http://bmjopen.bmj.com/site/about/guidelines.xhtml

Supplementary results

Sub-group analysis

Data from healthy participants (n=36) scanned on the 1.5T Siemens MRI scanner were compared to the sub-group of patients (N=121) scanned on the same MRI machine. Median global cardiac T1 was elevated in the patient group (979 ms versus 962ms, P=0.001). Lung fractional area difference, a measure of relaxed vital capacity, was significantly lower in the patient group (41% versus 48%, P<.001). Kidney inflammation (1148 vs 1084 ms, p <0.001) was significantly elevated in the patients as were markers of organ fat (liver 2.6% versus 2.1%, p=0.008; pancreas: 4.3% versus 2.5%, p<0.001) (**Figure S1)**.

Figure S1: Box plots showing median and interquartile ranges for the healthy control group and the patient group for those scanned at 1.5T. Comparisons between groups were performed using two-sided Kolmogorov-Smirnov (KS) tests. Significance stars are * P<.05; ** P<.01, ***P<.001.

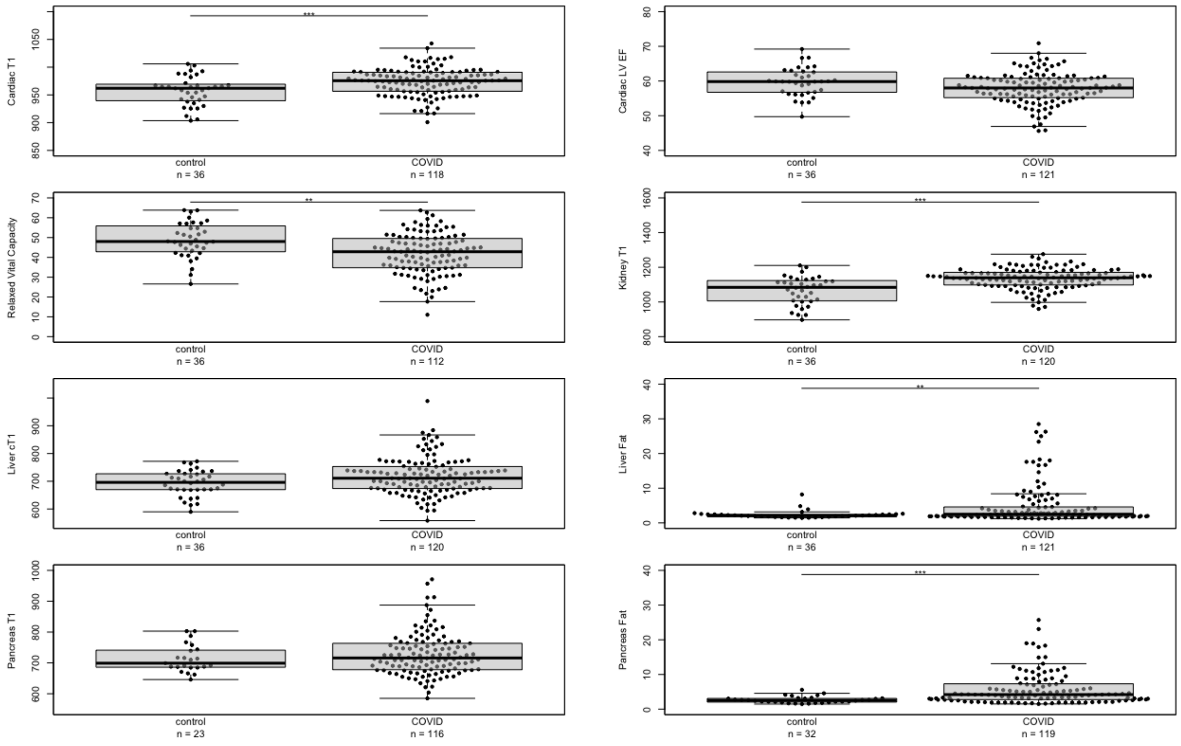

For peer review only - http://bmjopen.bmj.com/site/about/guidelines.xhtml

Figure S2: Organ impairment in severe versus moderate post COVID syndrome (n=201)

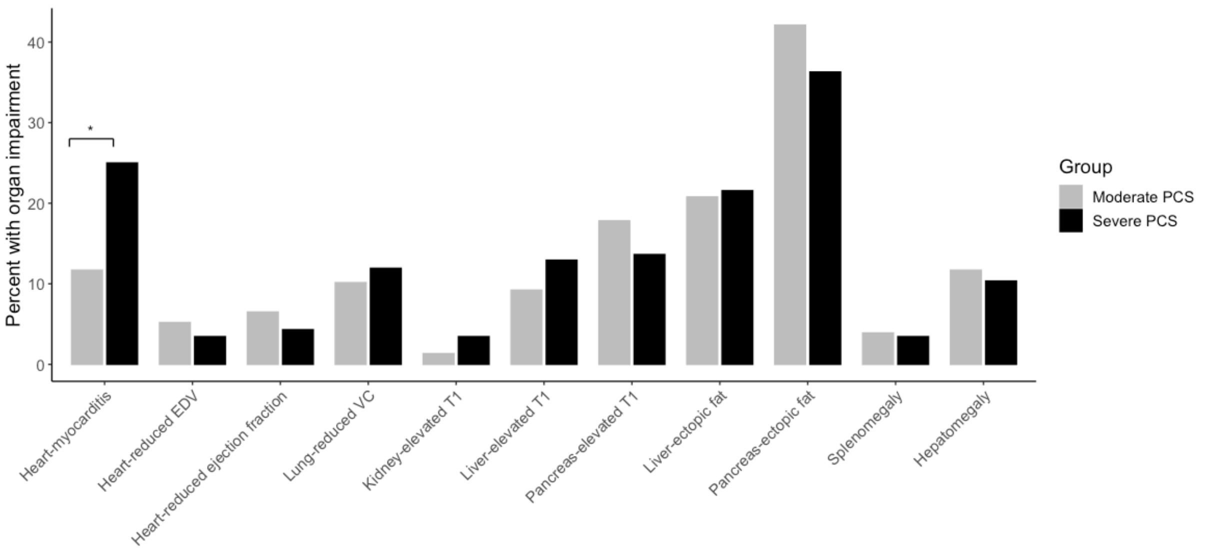

Table S1: Reference ranges for organ impairment, defined as a value that was greater than the mean plus 2 standard deviations of that from the control group for most; mean minus 2 standard deviations for left ventricular ejection fraction and lung fractional area difference for the 1.5T scans. For the 3T scans, this was the value as reported by Raman et al (2020).

	1.5T Reference range	3T reference range
Left ventricular ejection fraction (LVEF) (S4-S7)	≤ 51.5%	----
Increased end-diastolic volume (S4-S7)	≥ 264ml in men<br>≥ 206ml in women	----
Myocarditis (S4-S7)	≥ 1015 ms	≥ 1238ms
Deep breathing fractional area change*	≤ 31%	----
Liver volume (S8-S11)	≤ 1.93L	----
Liver fat (S8-S11)	≥ 4.8%	----
Liver inflammation (S8-S11)	≥ 784 ms	----
Pancreatic fat (S12-S13)	≥ 4.6%	----
Pancreatic inflammation (S12-13)	≥ 803ms	----
Renal Cortical T1(S14-S15)	≥ 1227ms	≥ 1652ms
Spleen volume(S16)	≤ 0.35L	----

** Our lung imaging protocol captured 2D dynamic imaging of the lungs as the patient breathes. We delineated the lungs at maximum inspiration and again at maximum expiration and take the difference to give a proxy of 'vital capacity', which correlates well with forced vital capacity (r = 0.61, P<.001) from spirometry. Given the measure was associated with body size, we divided the difference in maximum inspiration and expiration by maximum inspiration to give a normalised 'lung ejection fraction'. In order to assess whether an individual's 'lung ejection fraction' was abnormal, it was measured in 39 controls, characterising a healthy normal range of the mean +/- 2 standard deviations, with a lower score representing poorer lung health. 31% (0.31) was the lower limit for normal from our controls and therefore selected as the threshold for respiratory impairment.*

For peer review only - http://bmjopen.bmj.com/site/about/guidelines.xhtml

Table S2: Blood investigations in 201 low-risk individuals with post-COVID syndrome, sub-divided by those who were hospitalised versus those who were managed at home

Measurement	All	Managed at home	Hospitalised	p-value
Haemoglobin				
• Normal (130 - 170 g/L in men; 115 - 155 g/L in women)	170 (95.5%)	140 (95.9%)	30 (93.8%)	0.575
• Abnormal low (< 130 g/L in men; < 115 g/L in women)	5 (2.8%)	4 (2.7%)	1 (3.1%)	
• Abnormal high (> 170 g/L in men; > 155 g/L in women)	3 (1.7%)	2 (1.4%)	1 (3.1%)	
Haematocrit (HCT)				
• Normal (0.37 - 0.5 in men; 0.33 - 0.45 in women)	173 (97.2%)	142 (97.3%)	31 (96.9%)	0.386
• Abnormal low (< 0.37 in men; < 0.33 in women)	2 (1.1%)	1 (0.7%)	1 (3.1%)	
• Abnormal high (> 0.5 in men; > 0.45 in women)	3 (1.7%)	3 (2.1%)	0 (0%)	
Red cell count				
• Normal (4.4 - 5.8 x10^12/L in men; 3.95 - 5.15 x10^12/L in women)	170 (95.5%)	140 (95.9%)	30 (93.8%)	0.287
• Abnormal low (< 4.4 x10^12/L in men; < 3.95 x10^12/L in women)	5 (2.8%)	3 (2.1%)	2 (6.2%)	
• Abnormal high (> 5.8 x10^12/L in men; > 5.15 x10^12/L in women)	3 (1.7%)	3 (2.1%)	0 (0%)	
Mean cell volume (MCV)				
• Normal (80 - 99 fL)	174 (97.8%)	142 (97.3%)	32 (100%)	1
• Abnormal low (< 80 fL)	4 (2.2%)	4 (2.7%)	0 (0%)	
• Abnormal high (> 99 fL)	0 (0%)	0 (0%)	0 (0%)	
Mean corpuscular haemoglobin (MCH)				
• Normal (26 - 33.5 pg)	174 (97.8%)	143 (97.9%)	31 (96.9%)	0.249
• Abnormal low (< 26 pg)	3 (1.7%)	3 (2.1%)	0 (0%)	
• Abnormal high (> 33.5 pg)	1 (0.6%)	0 (0%)	1 (3.1%)	
Mean corpuscular haemoglobin concentration (MCHC)				
• Normal (300 - 350 g/L)	135 (75.8%)	109 (74.7%)	26 (81.2%)	0.501
• Abnormal low (< 300 g/L)	0 (0%)	0 (0%)	0 (0%)	
• Abnormal high (> 350 g/L)	43 (24.2%)	37 (25.3%)	6 (18.8%)	
Red cell distribution width (RDW)				
• Normal (11.5 - 15)	161 (91%)	129 (89%)	32 (100%)	0.218
• Abnormal low (< 11.5)	10 (5.6%)	10 (6.9%)	0 (0%)	
• Abnormal high (> 15)	6 (3.4%)	6 (4.1%)	0 (0%)	
Platelet count				
• Normal (150 - 400 x10^9/L)	166 (93.3%)	138 (94.5%)	28 (87.5%)	0.152
• Abnormal low (< 150 x10^9/L)	2 (1.1%)	2 (1.4%)	0 (0%)	
• Abnormal high (> 400 x10^9/L)	10 (5.6%)	6 (4.1%)	4 (12.5%)	
Mean platelet volume (MPV)				
• Normal (7 - 13 fL)	177 (99.4%)	145 (99.3%)	32 (100%)	1
• Abnormal low (< 7 fL)	0 (0%)	0 (0%)	0 (0%)	
• Abnormal high (> 13 fL)	1 (0.6%)	1 (0.7%)	0 (0%)	
White cell count				

For peer review only - http://bmjopen.bmj.com/site/about/guidelines.xhtml

• Normal (3 - 10 x10^9/L)	172 (96.6%)	140 (95.9%)	32 (100%)	0.593
• Abnormal low (< 3 x10^9/L)	0 (0%)	0 (0%)	0 (0%)	
• Abnormal high (> 10 x10^9/L)	6 (3.4%)	6 (4.1%)	0 (0%)	
Neutrophils				
• Normal (2 - 7.5 x10^9/L)	163 (91.6%)	133 (91.1%)	30 (93.8%)	1
• Abnormal low (< 2 x10^9/L)	12 (6.7%)	10 (6.8%)	2 (6.2%)	
• Abnormal high (> 7.5 x10^9/L)	3 (1.7%)	3 (2.1%)	0 (0%)	
Lymphocytes				
• Normal (1.2 - 3.65 x10^9/L)	161 (90.4%)	130 (89%)	31 (96.9%)	0.316
• Abnormal low (< 1.2 x10^9/L)	17 (9.6%)	16 (11%)	1 (3.1%)	
• Abnormal high (> 3.65 x10^9/L)	0 (0%)	0 (0%)	0 (0%)	
Monocytes				
• Normal (0.2 - 1 x10^9/L)	176 (98.9%)	144 (98.6%)	32 (100%)	1
• Abnormal low (< 0.2 x10^9/L)	1 (0.6%)	1 (0.7%)	0 (0%)	
• Abnormal high (> 1 x10^9/L)	1 (0.6%)	1 (0.7%)	0 (0%)	
Eosinophils				
• Normal (0 - 0.4 x10^9/L)	172 (96.6%)	141 (96.6%)	31 (96.9%)	1
• Abnormal low (< 0 x10^9/L)	0 (0%)	0 (0%)	0 (0%)	
• Abnormal high (> 0.4 x10^9/L)	6 (3.4%)	5 (3.4%)	1 (3.1%)	
Basophils				
• Normal (0 - 0.1 x10^9/L)	178 (100%)	146 (100%)	32 (100%)	N/A
• Abnormal low (< 0 x10^9/L)	0 (0%)	0 (0%)	0 (0%)	
• Abnormal high (> 0.1 x10^9/L)	0 (0%)	0 (0%)	0 (0%)	
Erythrocyte sedimentation rate (ESR)				
• Normal (1 - 20 mm/hr)	164 (91.1%)	136 (91.9%)	28 (87.5%)	0.491
• Abnormal low (< 1 mm/hr)	0 (0%)	0 (0%)	0 (0%)	
• Abnormal high (> 20 mm/hr)	16 (8.9%)	12 (8.1%)	4 (12.5%)	
Sodium				
• Normal (135 - 145 mmol/L)	173 (97.2%)	141 (96.6%)	32 (100%)	1
• Abnormal low (< 135 mmol/L)	4 (2.2%)	4 (2.7%)	0 (0%)	
• Abnormal high (> 145 mmol/L)	1 (0.6%)	1 (0.7%)	0 (0%)	
Potassium				
• Normal (3.5 - 5.1 mmol/L)	108 (62.1%)	87 (61.3%)	21 (65.6%)	0.692
• Abnormal low (< 3.5 mmol/L)	0 (0%)	0 (0%)	0 (0%)	
• Abnormal high (> 5.1 mmol/L)	66 (37.9%)	55 (38.7%)	11 (34.4%)	
Chloride				
• Normal (98 - 107 mmol/L)	171 (96.1%)	139 (95.2%)	32 (100%)	1
• Abnormal low (< 98 mmol/L)	4 (2.2%)	4 (2.7%)	0 (0%)	
• Abnormal high (> 107 mmol/L)	3 (1.7%)	3 (2.1%)	0 (0%)	
Bicarbonate				
• Normal (22 - 29 mmol/L)	150 (84.3%)	125 (85.6%)	25 (78.1%)	0.169
• Abnormal low (< 22 mmol/L)	18 (10.1%)	15 (10.3%)	3 (9.4%)	
• Abnormal high (> 29 mmol/L)	10 (5.6%)	6 (4.1%)	4 (12.5%)	
Urea				

• Normal (1.7 - 8.3 mmol/L)	178 (100%)	146 (100%)	32 (100%)	N/A
• Abnormal low (< 1.7 mmol/L)	0 (0%)	0 (0%)	0 (0%)	
• Abnormal high (> 8.3 mmol/L)	0 (0%)	0 (0%)	0 (0%)	
Creatinine				
• Normal (66 - 112 umol/L in men; 49 - 92 umol/L in women)	161 (90.4%)	134 (91.8%)	27 (84.4%)	0.219
• Abnormal low (< 66 umol/L in men; < 49 umol/L in women)	12 (6.7%)	9 (6.2%)	3 (9.4%)	
• Abnormal high (> 112 umol/L in men; > 92 umol/L in women)	5 (2.8%)	3 (2.1%)	2 (6.2%)	
Bilirubin				
• Normal (0 - 20 umol/L)	175 (98.3%)	144 (98.6%)	31 (96.9%)	0.45
• Abnormal low (< 0 umol/L)	0 (0%)	0 (0%)	0 (0%)	
• Abnormal high (> 20 umol/L)	3 (1.7%)	2 (1.4%)	1 (3.1%)	
Alkaline phosphatase				
• Normal (40 - 129 IU/L in men; 35 - 104 IU/L in women)	168 (94.4%)	137 (93.8%)	31 (96.9%)	0.161
• Abnormal low (< 40 IU/L in men; < 35 IU/L in women)	8 (4.5%)	8 (5.5%)	0 (0%)	
• Abnormal high (> 129 IU/L in men; > 104 IU/L in women)	2 (1.1%)	1 (0.7%)	1 (3.1%)	
Aspartate transferase				
• Normal (0 - 37 IU/L in men; 0 - 31 IU/L in women)	162 (93.1%)	133 (93.7%)	29 (90.6%)	0.464
• Abnormal low (< 0 IU/L in men; < 0 IU/L in women)	0 (0%)	0 (0%)	0 (0%)	
• Abnormal high (> 37 IU/L in men; > 31 IU/L in women)	12 (6.9%)	9 (6.3%)	3 (9.4%)	
Alanine transferase				
• Normal (10 - 50 IU/L in men; 10 - 35 IU/L in women)	151 (84.8%)	125 (85.6%)	26 (81.2%)	0.603
• Abnormal low (< 10 IU/L in men; < 10 IU/L in women)	2 (1.1%)	2 (1.4%)	0 (0%)	
• Abnormal high (> 50 IU/L in men; > 35 IU/L in women)	25 (14%)	19 (13%)	6 (18.8%)	
Lactate dehydrogenase (LDH)				
• Normal (135 - 225 IU/L in men; 135 - 214 IU/L in women)	142 (80.7%)	118 (81.9%)	24 (75%)	0.236
• Abnormal low (< 135 IU/L in men; < 135 IU/L in women)	5 (2.8%)	5 (3.5%)	0 (0%)	
• Abnormal high (> 225 IU/L in men; > 214 IU/L in women)	29 (16.5%)	21 (14.6%)	8 (25%)	
Creatinine kinase (CK)				
• Normal (38 - 204 IU/L in men; 26 - 140 IU/L in women)	163 (91.6%)	132 (90.4%)	31 (96.9%)	0.642
• Abnormal low (< 38 IU/L in men; < 26 IU/L in women)	2 (1.1%)	2 (1.4%)	0 (0%)	
• Abnormal high (> 204 IU/L in men; > 140 IU/L in women)	13 (7.3%)	12 (8.2%)	1 (3.1%)	
Gamma glutamyl transferase				
• Normal (10 - 71 IU/L in men; 6 - 42 IU/L in women)	165 (92.7%)	136 (93.2%)	29 (90.6%)	0.461
• Abnormal low (< 10 IU/L in men; < 6 IU/L in women)	4 (2.2%)	4 (2.7%)	0 (0%)	
• Abnormal high (> 71 IU/L in men; > 42 IU/L in women)	9 (5.1%)	6 (4.1%)	3 (9.4%)	
Total protein				
• Normal (63 - 83 g/L)	173 (97.2%)	143 (97.9%)	30 (93.8%)	0.22
• Abnormal low (< 63 g/L)	3 (1.7%)	2 (1.4%)	1 (3.1%)	
• Abnormal high (> 83 g/L)	2 (1.1%)	1 (0.7%)	1 (3.1%)	
Albumin				
• Normal (34 - 50 g/L)	167 (93.8%)	136 (93.2%)	31 (96.9%)	0.692
• Abnormal low (< 34 g/L)	0 (0%)	0 (0%)	0 (0%)	
• Abnormal high (> 50 g/L)	11 (6.2%)	10 (6.8%)	1 (3.1%)	
Globulin				

For peer review only - http://bmjopen.bmj.com/site/about/guidelines.xhtml

• Normal (19 - 35 g/L)	173 (97.2%)	142 (97.3%)	31 (96.9%)	0.386
• Abnormal low (< 19 g/L)	3 (1.7%)	3 (2.1%)	0 (0%)	
• Abnormal high (> 35 g/L)	2 (1.1%)	1 (0.7%)	1 (3.1%)	
Calcium				
• Normal (2.2 - 2.6 mmol/L)	172 (96.6%)	141 (96.6%)	31 (96.9%)	0.43
• Abnormal low (< 2.2 mmol/L)	2 (1.1%)	1 (0.7%)	1 (3.1%)	
• Abnormal high (> 2.6 mmol/L)	4 (2.2%)	4 (2.7%)	0 (0%)	
Magnesium				
• Normal (0.6 - 1 mmol/L)	176 (98.9%)	144 (98.6%)	32 (100%)	1
• Abnormal low (< 0.6 mmol/L)	1 (0.6%)	1 (0.7%)	0 (0%)	
• Abnormal high (> 1 mmol/L)	1 (0.6%)	1 (0.7%)	0 (0%)	
Phosphate				
• Normal (0.87 - 1.45 mmol/L)	150 (84.3%)	121 (82.9%)	29 (90.6%)	0.518
• Abnormal low (< 0.87 mmol/L)	23 (12.9%)	21 (14.4%)	2 (6.2%)	
• Abnormal high (> 1.45 mmol/L)	5 (2.8%)	4 (2.7%)	1 (3.1%)	
Uric acid				
• Normal (266 - 474 umol/L in men; 175 - 363 umol/L in women)	148 (83.1%)	124 (84.9%)	24 (75%)	0.067
• Abnormal low (< 266 umol/L in men; < 175 umol/L in women)	19 (10.7%)	16 (11%)	3 (9.4%)	
• Abnormal high (> 474 umol/L in men; > 363 umol/L in women)	11 (6.2%)	6 (4.1%)	5 (15.6%)	
Triglycerides				
• Normal (< 2.3 mmol/L)	10 (100%)	8 (100%)	2 (100%)	N/A
• Abnormal high (> 2.3 mmol/L)	0 (0%)	0 (0%)	0 (0%)	
Fasting triglycerides				
• Normal (< 2.3 mmol/L)	149 (88.7%)	128 (92.8%)	21 (70%)	0.002
• Abnormal high (> 2.3 mmol/L)	19 (11.3%)	10 (7.2%)	9 (30%)	
Cholesterol				
• Normal (< 5 mmol/L)	4 (40%)	3 (37.5%)	1 (50%)	1
• Abnormal high (> 5 mmol/L)	6 (60%)	5 (62.5%)	1 (50%)	
Fasting cholesterol				
• Normal (< 5 mmol/L)	98 (58.3%)	86 (62.3%)	12 (40%)	0.04
• Abnormal high (> 5 mmol/L)	70 (41.7%)	52 (37.7%)	18 (60%)	
HDL cholesterol				
• Normal (0.9 - 1.5 mmol/L in men; 1.2 - 1.7 mmol/L in women)	106 (59.6%)	87 (59.6%)	19 (59.4%)	0.075
• Abnormal low (< 0.9 mmol/L in men; < 1.2 mmol/L in women)	16 (9%)	10 (6.8%)	6 (18.8%)	
• Abnormal high (> 1.5 mmol/L in men; > 1.7 mmol/L in women)	56 (31.5%)	49 (33.6%)	7 (21.9%)	
LDL cholesterol				
• Normal (< 3 mmol/L)	113 (64.9%)	100 (69.4%)	13 (43.3%)	0.011
• Abnormal high (> 3 mmol/L)	61 (35.1%)	44 (30.6%)	17 (56.7%)	
Iron				
• Normal (10.6 - 28.3 umol/L in men; 6.6 - 26 umol/L in women)	164 (92.1%)	135 (92.5%)	29 (90.6%)	0.22
• Abnormal low (< 10.6 umol/L in men; < 6.6 umol/L in women)	4 (2.2%)	2 (1.4%)	2 (6.2%)	

For peer review only - http://bmjopen.bmj.com/site/about/guidelines.xhtml

• Abnormal high (> 28.3 umol/L in men; > 26 umol/L in women)	10 (5.6%)	9 (6.2%)	1 (3.1%)	
Total iron binding capacity (TIBC)				
• Normal (41 - 77 umol/L)	172 (97.2%)	141 (97.2%)	31 (96.9%)	1
• Abnormal low (< 41 umol/L)	0 (0%)	0 (0%)	0 (0%)	
• Abnormal high (> 77 umol/L)	5 (2.8%)	4 (2.8%)	1 (3.1%)	
Transferrin saturation				
• Normal (20 - 55 %)	139 (78.5%)	120 (82.8%)	19 (59.4%)	0.011
• Abnormal low (< 20 %)	34 (19.2%)	22 (15.2%)	12 (37.5%)	
• Abnormal high (> 55 %)	4 (2.3%)	3 (2.1%)	1 (3.1%)	
High sensitivity CRP				
• Normal (0 - 5 mg/L)	146 (92.4%)	124 (93.9%)	22 (84.6%)	0.112
• Abnormal low (< 0 mg/L)	0 (0%)	0 (0%)	0 (0%)	
• Abnormal high (> 5 mg/L)	12 (7.6%)	8 (6.1%)	4 (15.4%)	

For peer review only

Table S3: Blood investigations in 201 low-risk individuals sub-divided by those with severe or moderate post-COVID syndrome (PCS)

Measurement	All	Moderate PCS	Severe PCS	p-value
Haemoglobin				
• Normal (130 - 170 g/L in men; 115 - 155 g/L in women)	166 (96%)	62 (96.9%)	104 (95.4%)	1
• Abnormal low (< 130 g/L in men; < 115 g/L in women)	4 (2.3%)	1 (1.6%)	3 (2.8%)	
• Abnormal high (> 170 g/L in men; > 155 g/L in women)	3 (1.7%)	1 (1.6%)	2 (1.8%)	
Haematocrit (HCT)				
• Normal (0.37 - 0.5 in men; 0.33 - 0.45 in women)	168 (97.1%)	64 (100%)	104 (95.4%)	0.274
• Abnormal low (< 0.37 in men; < 0.33 in women)	2 (1.2%)	0 (0%)	2 (1.8%)	
• Abnormal high (> 0.5 in men; > 0.45 in women)	3 (1.7%)	0 (0%)	3 (2.8%)	
Red cell count				
• Normal (4.4 - 5.8 x10^12/L in men; 3.95 - 5.15 x10^12/L in women)	167 (96.5%)	61 (95.3%)	106 (97.2%)	0.825
• Abnormal low (< 4.4 x10^12/L in men; < 3.95 x10^12/L in women)	4 (2.3%)	2 (3.1%)	2 (1.8%)	
• Abnormal high (> 5.8 x10^12/L in men; > 5.15 x10^12/L in women)	2 (1.2%)	1 (1.6%)	1 (0.9%)	
Mean cell volume (MCV)				
• Normal (80 - 99 fL)	170 (98.3%)	62 (96.9%)	108 (99.1%)	0.556
• Abnormal low (< 80 fL)	3 (1.7%)	2 (3.1%)	1 (0.9%)	
• Abnormal high (> 99 fL)	0 (0%)	0 (0%)	0 (0%)	
Mean corpuscular haemoglobin (MCH)				
• Normal (26 - 33.5 pg)	170 (98.3%)	61 (95.3%)	109 (100%)	0.049
• Abnormal low (< 26 pg)	2 (1.2%)	2 (3.1%)	0 (0%)	
• Abnormal high (> 33.5 pg)	1 (0.6%)	1 (1.6%)	0 (0%)	
Mean corpuscular haemoglobin concentration (MCHC)				
• Normal (300 - 350 g/L)	131 (75.7%)	53 (82.8%)	78 (71.6%)	0.103
• Abnormal low (< 300 g/L)	0 (0%)	0 (0%)	0 (0%)	
• Abnormal high (> 350 g/L)	42 (24.3%)	11 (17.2%)	31 (28.4%)	
Red cell distribution width (RDW)				
• Normal (11.5 - 15)	157 (91.3%)	59 (92.2%)	98 (90.7%)	0.339
• Abnormal low (< 11.5)	10 (5.8%)	2 (3.1%)	8 (7.4%)	
• Abnormal high (> 15)	5 (2.9%)	3 (4.7%)	2 (1.9%)	
Platelet count				
• Normal (150 - 400 x10^9/L)	161 (93.1%)	59 (92.2%)	102 (93.6%)	0.417
• Abnormal low (< 150 x10^9/L)	2 (1.2%)	0 (0%)	2 (1.8%)	
• Abnormal high (> 400 x10^9/L)	10 (5.8%)	5 (7.8%)	5 (4.6%)	
Mean platelet volume (MPV)				
• Normal (7 - 13 fL)	172 (99.4%)	64 (100%)	108 (99.1%)	1
• Abnormal low (< 7 fL)	0 (0%)	0 (0%)	0 (0%)	

For peer review only - http://bmjopen.bmj.com/site/about/guidelines.xhtml

• Abnormal high (> 13 fL)	1 (0.6%)	0 (0%)	1 (0.9%)	
White cell count				
• Normal (3 - 10 x10^9/L)	167 (96.5%)	61 (95.3%)	106 (97.2%)	0.671
• Abnormal low (< 3 x10^9/L)	0 (0%)	0 (0%)	0 (0%)	
• Abnormal high (> 10 x10^9/L)	6 (3.5%)	3 (4.7%)	3 (2.8%)	
Neutrophils				
• Normal (2 - 7.5 x10^9/L)	159 (91.9%)	57 (89.1%)	102 (93.6%)	0.468
• Abnormal low (< 2 x10^9/L)	11 (6.4%)	5 (7.8%)	6 (5.5%)	
• Abnormal high (> 7.5 x10^9/L)	3 (1.7%)	2 (3.1%)	1 (0.9%)	
Lymphocytes				
• Normal (1.2 - 3.65 x10^9/L)	156 (90.2%)	56 (87.5%)	100 (91.7%)	0.43
• Abnormal low (< 1.2 x10^9/L)	17 (9.8%)	8 (12.5%)	9 (8.3%)	
• Abnormal high (> 3.65 x10^9/L)	0 (0%)	0 (0%)	0 (0%)	
Monocytes				
• Normal (0.2 - 1 x10^9/L)	171 (98.8%)	63 (98.4%)	108 (99.1%)	0.604
• Abnormal low (< 0.2 x10^9/L)	1 (0.6%)	0 (0%)	1 (0.9%)	
• Abnormal high (> 1 x10^9/L)	1 (0.6%)	1 (1.6%)	0 (0%)	
Eosinophils				
• Normal (0 - 0.4 x10^9/L)	167 (96.5%)	63 (98.4%)	104 (95.4%)	0.415
• Abnormal low (< 0 x10^9/L)	0 (0%)	0 (0%)	0 (0%)	
• Abnormal high (> 0.4 x10^9/L)	6 (3.5%)	1 (1.6%)	5 (4.6%)	
Basophils				
• Normal (0 - 0.1 x10^9/L)	173 (100%)	64 (100%)	109 (100%)	N/A
• Abnormal low (< 0 x10^9/L)	0 (0%)	0 (0%)	0 (0%)	
• Abnormal high (> 0.1 x10^9/L)	0 (0%)	0 (0%)	0 (0%)	
Erythrocyte sedimentation rate (ESR)				
• Normal (1 - 20 mm/hr)	160 (91.4%)	62 (93.9%)	98 (89.9%)	0.416
• Abnormal low (< 1 mm/hr)	0 (0%)	0 (0%)	0 (0%)	
• Abnormal high (> 20 mm/hr)	15 (8.6%)	4 (6.1%)	11 (10.1%)	
Sodium				
• Normal (135 - 145 mmol/L)	168 (97.1%)	63 (98.4%)	105 (96.3%)	1
• Abnormal low (< 135 mmol/L)	4 (2.3%)	1 (1.6%)	3 (2.8%)	
• Abnormal high (> 145 mmol/L)	1 (0.6%)	0 (0%)	1 (0.9%)	
Potassium				
• Normal (3.5 - 5.1 mmol/L)	105 (62.1%)	35 (56.5%)	70 (65.4%)	0.255
• Abnormal low (< 3.5 mmol/L)	0 (0%)	0 (0%)	0 (0%)	
• Abnormal high (> 5.1 mmol/L)	64 (37.9%)	27 (43.5%)	37 (34.6%)	
Chloride				
• Normal (98 - 107 mmol/L)	166 (96%)	62 (96.9%)	104 (95.4%)	1
• Abnormal low (< 98 mmol/L)	4 (2.3%)	1 (1.6%)	3 (2.8%)	
• Abnormal high (> 107 mmol/L)	3 (1.7%)	1 (1.6%)	2 (1.8%)	

For peer review only - http://bmjopen.bmj.com/site/about/guidelines.xhtml

Bicarbonate				
• Normal (22 - 29 mmol/L)	147 (85%)	55 (85.9%)	92 (84.4%)	0.946
• Abnormal low (< 22 mmol/L)	16 (9.2%)	6 (9.4%)	10 (9.2%)	
• Abnormal high (> 29 mmol/L)	10 (5.8%)	3 (4.7%)	7 (6.4%)	
Urea				
• Normal (1.7 - 8.3 mmol/L)	173 (100%)	64 (100%)	109 (100%)	N/A
• Abnormal low (< 1.7 mmol/L)	0 (0%)	0 (0%)	0 (0%)	
• Abnormal high (> 8.3 mmol/L)	0 (0%)	0 (0%)	0 (0%)	
Creatinine				
• Normal (66 - 112 umol/L in men; 49 - 92 umol/L in women)	156 (90.2%)	59 (92.2%)	97 (89%)	0.705
• Abnormal low (< 66 umol/L in men; < 49 umol/L in women)	12 (6.9%)	3 (4.7%)	9 (8.3%)	
• Abnormal high (> 112 umol/L in men; > 92 umol/L in women)	5 (2.9%)	2 (3.1%)	3 (2.8%)	
Bilirubin				
• Normal (0 - 20 umol/L)	170 (98.3%)	63 (98.4%)	107 (98.2%)	1
• Abnormal low (< 0 umol/L)	0 (0%)	0 (0%)	0 (0%)	
• Abnormal high (> 20 umol/L)	3 (1.7%)	1 (1.6%)	2 (1.8%)	
Alkaline phosphatase				
• Normal (40 - 129 IU/L in men; 35 - 104 IU/L in women)	164 (94.8%)	59 (92.2%)	105 (96.3%)	0.185
• Abnormal low (< 40 IU/L in men; < 35 IU/L in women)	7 (4%)	3 (4.7%)	4 (3.7%)	
• Abnormal high (> 129 IU/L in men; > 104 IU/L in women)	2 (1.2%)	2 (3.1%)	0 (0%)	
Aspartate transferase				
• Normal (0 - 37 IU/L in men; 0 - 31 IU/L in women)	157 (92.9%)	59 (93.7%)	98 (92.5%)	1
• Abnormal low (< 0 IU/L in men; < 0 IU/L in women)	0 (0%)	0 (0%)	0 (0%)	
• Abnormal high (> 37 IU/L in men; > 31 IU/L in women)	12 (7.1%)	4 (6.3%)	8 (7.5%)	
Alanine transferase				
• Normal (10 - 50 IU/L in men; 10 - 35 IU/L in women)	146 (84.4%)	56 (87.5%)	90 (82.6%)	0.512
• Abnormal low (< 10 IU/L in men; < 10 IU/L in women)	2 (1.2%)	1 (1.6%)	1 (0.9%)	
• Abnormal high (> 50 IU/L in men; > 35 IU/L in women)	25 (14.5%)	7 (10.9%)	18 (16.5%)	
Lactate dehydrogenase (LDH)				
• Normal (135 - 225 IU/L in men; 135 - 214 IU/L in women)	137 (80.1%)	51 (81%)	86 (79.6%)	0.24
• Abnormal low (< 135 IU/L in men; < 135 IU/L in women)	5 (2.9%)	0 (0%)	5 (4.6%)	
• Abnormal high (> 225 IU/L in men; > 214 IU/L in women)	29 (17%)	12 (19%)	17 (15.7%)	
Creatinine kinase (CK)				
• Normal (38 - 204 IU/L in men; 26 - 140 IU/L in women)	159 (91.9%)	56 (87.5%)	103 (94.5%)	0.28
• Abnormal low (< 38 IU/L in men; < 26 IU/L in women)	2 (1.2%)	1 (1.6%)	1 (0.9%)	
• Abnormal high (> 204 IU/L in men; > 140 IU/L in women)	12 (6.9%)	7 (10.9%)	5 (4.6%)	
Gamma glutamyl transferase				
• Normal (10 - 71 IU/L in men; 6 - 42 IU/L in women)	161 (93.1%)	60 (93.8%)	101 (92.7%)	0.426
• Abnormal low (< 10 IU/L in men; < 6 IU/L in women)	3 (1.7%)	0 (0%)	3 (2.8%)	
• Abnormal high (> 71 IU/L in men; > 42 IU/L in women)	9 (5.2%)	4 (6.2%)	5 (4.6%)	
Total protein				
• Normal (63 - 83 g/L)	168 (97.1%)	63 (98.4%)	105 (96.3%)	0.792

• Abnormal low (< 63 g/L)	3 (1.7%)	1 (1.6%)	2 (1.8%)	
• Abnormal high (> 83 g/L)	2 (1.2%)	0 (0%)	2 (1.8%)	
Albumin				
• Normal (34 - 50 g/L)	162 (93.6%)	59 (92.2%)	103 (94.5%)	0.538
• Abnormal low (< 34 g/L)	0 (0%)	0 (0%)	0 (0%)	
• Abnormal high (> 50 g/L)	11 (6.4%)	5 (7.8%)	6 (5.5%)	
Globulin				
• Normal (19 - 35 g/L)	168 (97.1%)	61 (95.3%)	107 (98.2%)	0.616
• Abnormal low (< 19 g/L)	3 (1.7%)	2 (3.1%)	1 (0.9%)	
• Abnormal high (> 35 g/L)	2 (1.2%)	1 (1.6%)	1 (0.9%)	
Calcium				
• Normal (2.2 - 2.6 mmol/L)	167 (96.5%)	62 (96.9%)	105 (96.3%)	0.525
• Abnormal low (< 2.2 mmol/L)	2 (1.2%)	0 (0%)	2 (1.8%)	
• Abnormal high (> 2.6 mmol/L)	4 (2.3%)	2 (3.1%)	2 (1.8%)	
Magnesium				
• Normal (0.6 - 1 mmol/L)	171 (98.8%)	63 (98.4%)	108 (99.1%)	0.604
• Abnormal low (< 0.6 mmol/L)	1 (0.6%)	1 (1.6%)	0 (0%)	
• Abnormal high (> 1 mmol/L)	1 (0.6%)	0 (0%)	1 (0.9%)	
Phosphate				
• Normal (0.87 - 1.45 mmol/L)	145 (83.8%)	55 (85.9%)	90 (82.6%)	0.824
• Abnormal low (< 0.87 mmol/L)	23 (13.3%)	8 (12.5%)	15 (13.8%)	
• Abnormal high (> 1.45 mmol/L)	5 (2.9%)	1 (1.6%)	4 (3.7%)	
Uric acid				
• Normal (266 - 474 umol/L in men; 175 - 363 umol/L in women)	145 (83.8%)	53 (82.8%)	92 (84.4%)	0.804
• Abnormal low (< 266 umol/L in men; < 175 umol/L in women)	18 (10.4%)	8 (12.5%)	10 (9.2%)	
• Abnormal high (> 474 umol/L in men; > 363 umol/L in women)	10 (5.8%)	3 (4.7%)	7 (6.4%)	
Triglycerides				
• Normal (< 2.3 mmol/L)	10 (100%)	6 (100%)	4 (100%)	N/A
• Abnormal high (> 2.3 mmol/L)	0 (0%)	0 (0%)	0 (0%)	
Fasting triglycerides				
• Normal (< 2.3 mmol/L)	144 (88.3%)	52 (89.7%)	92 (87.6%)	0.802
• Abnormal high (> 2.3 mmol/L)	19 (11.7%)	6 (10.3%)	13 (12.4%)	
Cholesterol				
• Normal (< 5 mmol/L)	4 (40%)	3 (50%)	1 (25%)	0.571
• Abnormal high (> 5 mmol/L)	6 (60%)	3 (50%)	3 (75%)	
Fasting cholesterol				
• Normal (< 5 mmol/L)	96 (58.9%)	39 (67.2%)	57 (54.3%)	0.135
• Abnormal high (> 5 mmol/L)	67 (41.1%)	19 (32.8%)	48 (45.7%)	
HDL cholesterol				
• Normal (0.9 - 1.5 mmol/L in men; 1.2 - 1.7 mmol/L in women)	103 (59.5%)	38 (59.4%)	65 (59.6%)	0.539
• Abnormal low (< 0.9 mmol/L in men; < 1.2 mmol/L in women)	16 (9.2%)	4 (6.2%)	12 (11%)	

For peer review only - http://bmjopen.bmj.com/site/about/guidelines.xhtml

• Abnormal high (> 1.5 mmol/L in men; > 1.7 mmol/L in women)	54 (31.2%)	22 (34.4%)	32 (29.4%)	
**LDL cholesterol**				
• Normal (< 3 mmol/L)	111 (65.7%)	45 (72.6%)	66 (61.7%)	0.18
• Abnormal high (> 3 mmol/L)	58 (34.3%)	17 (27.4%)	41 (38.3%)	
**Iron**				
• Normal (10.6 - 28.3 umol/L in men; 6.6 - 26 umol/L in women)	160 (92.5%)	57 (89.1%)	103 (94.5%)	0.337
• Abnormal low (< 10.6 umol/L in men; < 6.6 umol/L in women)	3 (1.7%)	2 (3.1%)	1 (0.9%)	
• Abnormal high (> 28.3 umol/L in men; > 26 umol/L in women)	10 (5.8%)	5 (7.8%)	5 (4.6%)	
**Total iron binding capacity (TIBC)**				
• Normal (41 - 77 umol/L)	167 (97.1%)	60 (93.8%)	107 (99.1%)	0.064
• Abnormal low (< 41 umol/L)	0 (0%)	0 (0%)	0 (0%)	
• Abnormal high (> 77 umol/L)	5 (2.9%)	4 (6.2%)	1 (0.9%)	
**Transferrin saturation**				
• Normal (20 - 55 %)	135 (78.5%)	50 (78.1%)	85 (78.7%)	0.283
• Abnormal low (< 20 %)	33 (19.2%)	11 (17.2%)	22 (20.4%)	
• Abnormal high (> 55 %)	4 (2.3%)	3 (4.7%)	1 (0.9%)	
**High sensitivity CRP**				
• Normal (0 - 5 mg/L)	141 (92.2%)	50 (96.2%)	91 (90.1%)	0.223
• Abnormal low (< 0 mg/L)	0 (0%)	0 (0%)	0 (0%)	
• Abnormal high (> 5 mg/L)	12 (7.8%)	2 (3.8%)	10 (9.9%)	

For peer review only - http://bmjopen.bmj.com/site/about/guidelines.xhtml

STROBE Statement—checklist of items that should be included in reports of observational studies

	Item No	Recommendation	Pages
**Title and abstract**	1	(*a*) Indicate the study's design with a commonly used term in the title or the abstract	3
		(*b*) Provide in the abstract an informative and balanced summary of what was done and what was found	3
**Introduction**			
Background/rationale	2	Explain the scientific background and rationale for the investigation being reported	4
Objectives	3	State specific objectives, including any prespecified hypotheses	4
**Methods**			
Study design	4	Present key elements of study design early in the paper	5
Setting	5	Describe the setting, locations, and relevant dates, including periods of recruitment, exposure, follow-up, and data collection	5
Participants	6	(*a*) *Cohort study*—Give the eligibility criteria, and the sources and methods of selection of participants. Describe methods of follow-up<br>*Case-control study*—Give the eligibility criteria, and the sources and methods of case ascertainment and control selection. Give the rationale for the choice of cases and controls<br>*Cross-sectional study*—Give the eligibility criteria, and the sources and methods of selection of participants	5
		(*b*) *Cohort study*—For matched studies, give matching criteria and number of exposed and unexposed<br>*Case-control study*—For matched studies, give matching criteria and the number of controls per case	3
Variables	7	Clearly define all outcomes, exposures, predictors, potential confounders, and effect modifiers. Give diagnostic criteria, if applicable	8
Data sources/ measurement	8*	For each variable of interest, give sources of data and details of methods of assessment (measurement). Describe comparability of assessment methods if there is more than one group	5-6 and supplementary
Bias	9	Describe any efforts to address potential sources of bias	10
Study size	10	Explain how the study size was arrived at	Ongoing study
Quantitative variables	11	Explain how quantitative variables were handled in the analyses. If applicable, describe which groupings were chosen and why	6
Statistical methods	12	(*a*) Describe all statistical methods, including those used to control for confounding	6
		(*b*) Describe any methods used to examine subgroups and interactions	6
		(*c*) Explain how missing data were addressed	10
		(*d*) *Cohort study*—If applicable, explain how loss to follow-up was addressed	n/a ongoing observational study

For peer review only - http://bmjopen.bmj.com/site/about/guidelines.xhtml

		Case-control study—If applicable, explain how matching of cases and controls was addressed<br>*Cross-sectional study*—If applicable, describe analytical methods taking account of sampling strategy	
		(*e*) Describe any sensitivity analyses	n/a
**Results**			
Participants	13*	(a) Report numbers of individuals at each stage of study—eg numbers potentially eligible, examined for eligibility, confirmed eligible, included in the study, completing follow-up, and analysed	7<br>Figure 1
		(b) Give reasons for non-participation at each stage	Figure 1
		(c) Consider use of a flow diagram	Figure 1
Descriptive data	14*	(a) Give characteristics of study participants (eg demographic, clinical, social) and information on exposures and potential confounders	Table 1
		(b) Indicate number of participants with missing data for each variable of interest	Table 1
		(c) *Cohort study*—Summarise follow-up time (eg, average and total amount)	Table 1
Outcome data	15*	*Cohort study*—Report numbers of outcome events or summary measures over time	Table 1
		Case-control study—Report numbers in each exposure category, or summary measures of exposure	Table 1
		Cross-sectional study—Report numbers of outcome events or summary measures	n/a
Main results	16	(*a*) Give unadjusted estimates and, if applicable, confounder-adjusted estimates and their precision (eg, 95% confidence interval). Make clear which confounders were adjusted for and why they were included	8
		(*b*) Report category boundaries when continuous variables were categorized	Table 2
		(*c*) If relevant, consider translating estimates of relative risk into absolute risk for a meaningful time period	n/a
Other analyses	17	Report other analyses done—eg analyses of subgroups and interactions, and sensitivity analyses	n/a
**Discussion**			
Key results	18	Summarise key results with reference to study objectives	9
Limitations	19	Discuss limitations of the study, taking into account sources of potential bias or imprecision. Discuss both direction and magnitude of any potential bias	10
Interpretation	20	Give a cautious overall interpretation of results considering objectives, limitations, multiplicity of analyses, results from similar studies, and other relevant evidence	10
Generalisability	21	Discuss the generalisability (external validity) of the study results	10
**Other information**			
Funding	22	Give the source of funding and the role of the funders for the present study and, if applicable, for the original study on which the present article is based	12

For peer review only

For peer review only - http://bmjopen.bmj.com/site/about/guidelines.xhtml

*Give information separately for cases and controls in case-control studies and, if applicable, for exposed and unexposed groups in cohort and cross-sectional studies.

Note: An Explanation and Elaboration article discusses each checklist item and gives methodological background and published examples of transparent reporting. The STROBE checklist is best used in conjunction with this article (freely available on the Web sites of PLoS Medicine at http://www.plosmedicine.org/, Annals of Internal Medicine at http://www.annals.org/, and Epidemiology at http://www.epidem.com/). Information on the STROBE Initiative is available at www.strobe-statement.org.

For peer review only
